# A Novel Pyroptosis-Related lncRNAs Signature for Predicting the Prognosis of Kidney Renal Clear Cell Carcinoma and Its Associations with Immunity

**DOI:** 10.1155/2021/9997185

**Published:** 2021-10-18

**Authors:** Xinfang Tang, Anna Zhang, Yuanyuan Feng, Ying Su, Xiaoyu Wang, Feng Jiang, Jianxin Ma

**Affiliations:** ^1^Department of Nephrology, The Affiliated Lianyungang Oriental Hospital of Kangda College of Nanjing Medical University, The Affiliated Lianyungang Oriental Hospital of Xuzhou Medical University, The Affiliated Lianyungang Oriental Hospital of Bengbu Medical College, Lianyungang 222042, China; ^2^Department of Nephrology, The First Affiliated Hospital of Nanjing Medical University, Nanjing 210029, China; ^3^Department of Oncology, The Affiliated Lianyungang Oriental Hospital of Kangda College of Nanjing Medical University, The Affiliated Lianyungang Oriental Hospital of Xuzhou Medical University, The Affiliated Lianyungang Oriental Hospital of Bengbu Medical College, Lianyungang 222042, China; ^4^Department of Personnel Division, The Affiliated Lianyungang Oriental Hospital of Kangda College of Nanjing Medical University, The Affiliated Lianyungang Oriental Hospital of Xuzhou Medical University, The Affiliated Lianyungang Oriental Hospital of Bengbu Medical College, Lianyungang 222042, China; ^5^Department of Neonatology, Obstetrics and Gynecology Hospital of Fudan University, Shanghai 200011, China

## Abstract

The most common kind of kidney cancer with poor prognosis is clear cell renal cell carcinoma (ccRCC). Pyroptosis is shown to be an inflammatory type of programmed cell death in recent years. In this research, we utilized pyroptosis-related differentially expressed lncRNAs in ccRCC to develop a predictive multi-lncRNA signature. We uncovered 14 lncRNAs with different expression patterns that were linked to ccRCC prognosis. Kaplan–Meier analysis identified a signature of high-risk lncRNAs related to poor prognosis for ccRCC. Furthermore, the AUC of the lncRNA signature was 0.771, indicating that they can be used to predict ccRCC prognosis. In predicting ccRCC prognosis, our risk analysis approach outperformed standard clinicopathological characteristics. In the low-risk group, GSEA indicated tumor-related pathways. T-cell functions such as T-cell coinhibition and T-cell costimulation were found to be expressed differently in two groups. Immune checkpoints including PD-1, LAG3, CTLA4, and BTLA were also differently expressed between the two groups. In patients with ccRCC, we created a 14-lncRNA-based predictor as a robust prognostic and predictive tool for OS.

## 1. Introduction

Renal cell carcinoma (RCC) is the most prevalent and malignant kidney cancer in adults, accounting for 3.7% of all malignancies in adults globally [1]. Worldwide, RCC is the major cause of cancer-related morbidity and death [2]. The most prevalent subtype of renal cell carcinoma is clear cell renal cell carcinoma (ccRCC), and it is critical to understand the molecular alterations linked to malignant transformation and prolonged survival [3]. For subgroups of individuals with RCC, the American Joint Committee on Cancer (AJCC) pathological grade system and tumor lymph node metastasis (TNM) stage give useful but insufficient prognostic assessments. Because clinicopathological risk variables do not predict disease recurrence, treatment response, or survival, they are of little therapeutic use. There is a growing need to introduce new prognostic and predictive biomarkers to complement and enhance current staging systems, given growing evidence that the discovery and use of molecular biomarkers can contribute to prognostic assessment and the identification of potentially high-risk RCC patients. These might be exploited as therapeutic targets in the future.

Pyroptosis, a gasdermin-mediated inflammasome-induced programmed cell death, was initially discovered in myeloid cells infected with pathogens or bacteria in 1992 [4]. Pyroptosis is expected to play a critical role in bacterial and viral infection clearance by eliminating intracellular replication habitats and increasing the host's defensive responses [5]. Pathogen clearance efficiency may be reduced as a consequence of pyroptosis dysfunction, as well as a malfunction in the activation of adaptive immune responses, resulting in tissue damage [6]. More recently, evidence has accumulated that pyroptosis may be chemically generated in cancer cells without the presence of bacteria or viruses [7]. Because it may impact all phases of carcinogenesis, pyroptosis has become a hot topic in cancer research. Progress in understanding the morphological aspects and processes of pyroptosis will improve our knowledge of cancer and open up new avenues for cancer treatment. Long noncoding RNAs (lncRNAs) are a subset of RNA molecules with a length of 200 nucleotides that influence gene expression. In addition to gene regulation, lncRNA is involved in a number of biological regulatory mechanisms, including those linked to tumor incidence, development, and metastasis. There are currently just a few research studies on pyroptosis-related lncRNAs. However, few sequence-based studies have looked at pyroptosis-related lncRNA features and their association with OS in patients with ccRCC. Using data from The Cancer Genome Atlas (TCGA), we first generated predictive multi-lncRNA signatures of differentially expressed cell nucleopause-related lncRNAs. Then, the effects of pyroptosis-related mRNA and immune response on the prognosis of ccRCC were investigated.

## 2. Materials and Methods

### 2.1. Data Collection

RNA sequence data were extracted using the TCGA-KIRC database from 537 individuals (72 normal samples and 539 tumor samples).  Table S1 shows the clinical features of the patients. We discovered 33 genes linked to pyroptosis ( Table S2). The “limma” package in *R* software was used to identify the pyroptosis-related differentially expressed genes (DEGs) between KIRC and normal tissues. A *P* value <0.05 and |log_2_FC| ≥1 were considered significantly different, including both upregulated and downregulated. Then, the lncRNAs correlated with the pyroptosis-related DEGs in which correlation coefficient |*R*^2^|>0.5 and *P* < 0.01 were selected as significant pyroptosis-related lncRNAs. Age, gender, grade, stage, survival status, TMN, and survival duration were among the clinical-pathological data obtained from ccRCC patients. FDR < 0.05 and |log_2_FC|≥1 were used to determine if there was a noticeable difference in expression of pyroptosis-related lncRNAs. The biological pathways related to the DEGs were then evaluated using Gene Ontology (GO). Function analysis of biological processes (BP), molecular functions (MF), and cellular components (CC) was performed by using *R* software.

### 2.2. The Development of a Predictive Signature for Pyroptosis-Related lncRNAs

To create the pyroptosis-related lncRNAs signature, we used LASSO-penalized Cox regression, univariate Cox regression, and multivariate Cox regression to establish a calculation formula as follows:

(1) $$\begin{array}{c} \text{risk score}=\sum_{i=1}^{n}\exp\;i*\beta i. \end{array}$$

Here, *β* means the coefficient value and exp means the lncRNA expression level. Each ccRCC patient's associated risk score was also assessed. Based on the median score, the patients were categorized into two groups: low-risk group and high-risk group.

### 2.3. Construction of the Predictive Nomogram

In the KEGG database, gene set enrichment analysis (GSEA) was utilized to create lncRNA signatures, which were then searched in the TCGA-KIRC database. *P* < 0.05 was chosen as the statistical significance level, and the false discovery rate (FDR) was adjusted at *q* <0.25. The prognostic factors for predicting ccRCC patients' 1-, 3-, and 5-year OS were combined into a nomogram.

### 2.4. Immunity and Gene Expression Analyses

Simultaneously, based on pyroptosis-related lncRNA signature, the CIBERSORT [8, 9], ESTIMATE [10], MCP counter [11], single-sample gene set enrichment analysis (ssGSEA) [12], and TIMER algorithms [13] were compared to assess cellular components or cellular immune responses between high- and low-risk groups. Using a heat map, the disparities in the immunological response were discovered. Furthermore, ssGSEA was conducted to assess immune cell subpopulations between the two groups as well as measuring their capacity to defend tumor infiltration. Potential immune checkpoint has been found in the literature previously.

### 2.5. Cell Culture and Transfection

ccRCC cell lines (786-O, HEK293 T, Caki-1, ACHN) and normal kidney cell lines (HK-2) were acquired from the cell bank of Chinese Academy of Sciences (Shanghai). RPMI 1640 (Gibco, Gaithersburg, MD., USA) was cultured with 10% fetal bovine serum (HyClone, Logan, USA) and 1% penicillin/streptomycin (Gibco) in an incubator at 37°C and 5% CO_2_. Transfections were performed applying OPTI-MEM (Invitrogen) and Lipofectamine 3000 according to manufacturer's instructions. si-FOXD2-AS1 and siNC were bought from Tsingke (Nanjing, China) and introduced into cells at a concentration of 50 nM. The transfected cells were harvested at 24 h after transfection. All primers and the sequence of siRNA are listed at Table S3.

### 2.6. Cell Proliferation, Invasion, and Migration Assays

The Cell Counting Kit-8 (CCK-8) and colony formation assays were applied to explore the ability of proliferation of cancer cells in different groups. In CCK-8 experiment, a total of 2,500 cancer cells were added into each well of 96-well plate. 10 *μ*l of CCK-8 solution (Dojindo Laboratories, Kumamoto, Japan) was added into 96 wells, and then the absorbance of each well was analyzed at 450 nm after incubation at 37°C for 2 h. For colony formation experiment, 1,000 cells of different groups were added into each well of a six-well plate. The culture medium was changed every 72 h. Crystal violet and 4% paraformaldehyde were applied to stain and fix the cells when the appearance of colonies could be recognized. The wound healing and transwell assays were applied to explore the ability of cellular migration and invasion.

### 2.7. Statistical Analysis

Bioconductor packages in R software, version 3.6.2, were used to analyze the data. The Wilcoxon test and the unpaired Student's *t*-test were used to assess normally and nonnormally distributed data, respectively. Based on FDR, the Benjamini–Hochberg approach was utilized to determine the variably expressed lncRNAs. Using ssGSEA, ccRCC DEG was standardized and compared with the genome using “GSVA” (R package). Operating characteristic curves (ROC) and decision curve analysis (DCA) [14] were used to evaluate the sensitivity and specificity of the generated diagnostic factors for ccRCC compared to other clinicopathological factors. Logistic regression analysis and heat map were used to investigate the relationship between pyroptosis-associated lncRNAs and clinicopathological features. Kaplan–Meier survival analysis was used to evaluate the survival of patients with ccRCC based on the characteristics of lncRNAs associated with coking death. *P* < 0.05 was used to assess the statistical significance of each study.

## 3. Results

### 3.1. Pyroptosis-Related Gene Enrichment Analysis

**Figure 1:**
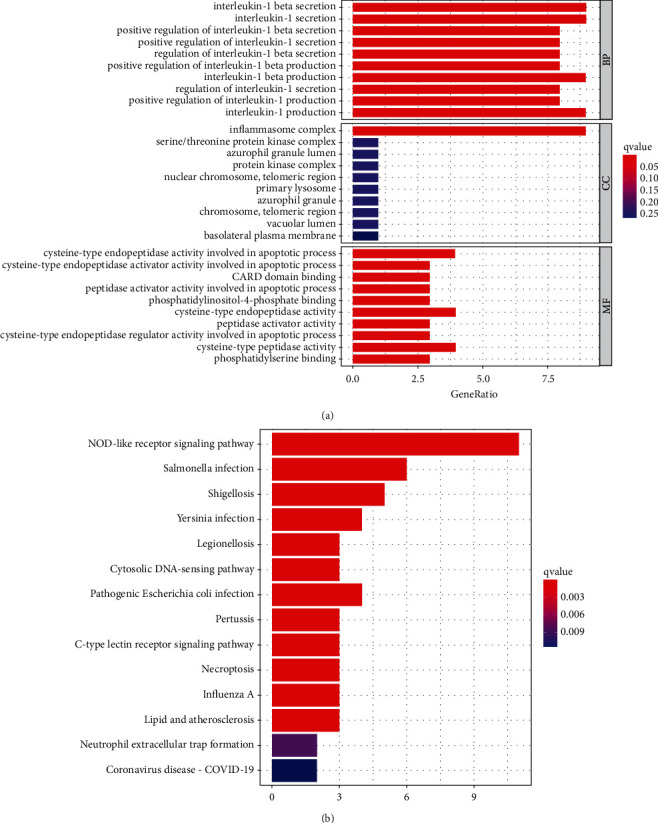
GO and KEGG analyses for pyroptosis-related differentially expressed genes. (a) GO and (b) KEGG. The predictive signature of pyroptosis-based lncRNAs.

We found 15 DEGs associated with pyroptosis (1 downregulated and 14 upregulated; Table S4). BP participated in interleukin-1 beta secretion, interleukin-1 secretion, interleukin-1 beta production, interleukin-1 production, positive regulation of interleukin-1 beta secretion. CC was mainly upregulated in inflammasome complex, serine/threonine protein kinase complex, azurophil granule lumen, protein kinase complex, nuclear chromosome, and telomeric region. MF was mainly upregulated in cysteine-type indopeptidase activity involved in the apoptotic process, cysteine-type endopeptidase activity, and cysteine-type peptidase activity. The overexpressed genes were mostly involved in the NOD-like receptor signaling pathway, according to KEGG analysis (Figure 1).

**Figure 2:**
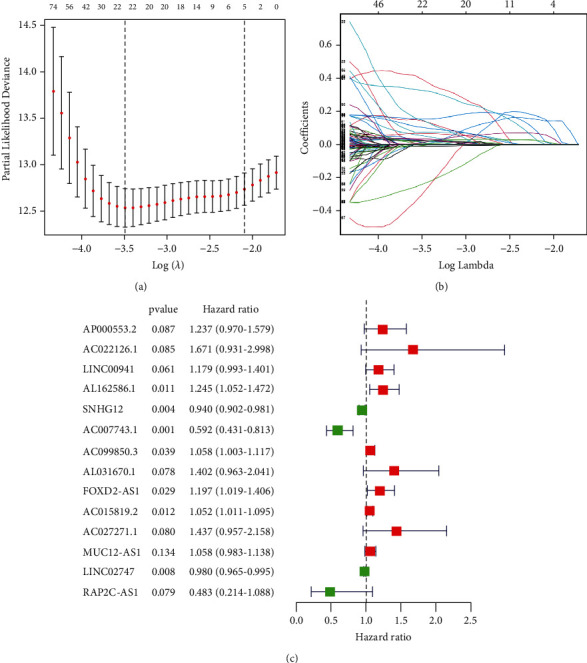
Construction of risk signature by LASSO and Cox regression analysis. (a) Cross-validation in the LASSO regression. (b) LASSO regression of the OS-related genes. (c) Multivariate Cox regression analysis revealed that the forest plot of pyroptosis-associated lncRNAs is substantially associated with OS in ccRCC patients.

We identified 1042 lncRNAs associated with pyroptosis (Table S5). Univariate Cox analysis found 299 potential pyroptosis-related lncRNAs (Table S6), and variables were chosen using LASSO regression and multivariable logistic regression analysis. 14 different lncRNAs (AP000533.2, AC022126.1, LINC00941, AL162586.1, SNHG12, AC007743.1, AC099850.3, AL031670.1, FOXD2-AS1, AC015819.2, AC027271.1, MUC12-AS1, LINC02747, and RAP2C-AS1) were revealed to be independent prognostic predictors of ccRCC (Figure 2). As a result, we computed risk ratings and developed a predictive signature for the lncRNAs.

### 3.2. Results of Survival Analysis and Multivariate Analysis

**Figure 3:**
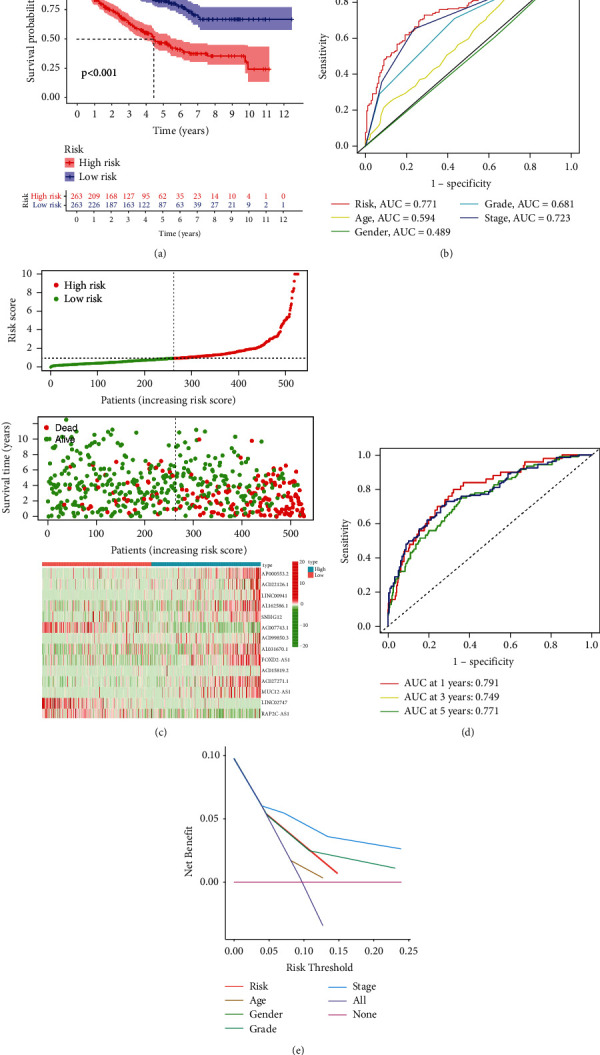
TCGA-based signature of pyroptosis-related lncRNAs. (a). K–M curves for the OS of patients in two groups. (b) ROC curves revealed the predictive efficiency of the risk score. (c) Distribution of patients based on the risk score. (d) AUC for predicting the survival rate of ccRCC patients after 1, 3, and 5 years. (e) DCA of the risk variables.

**Figure 4:**
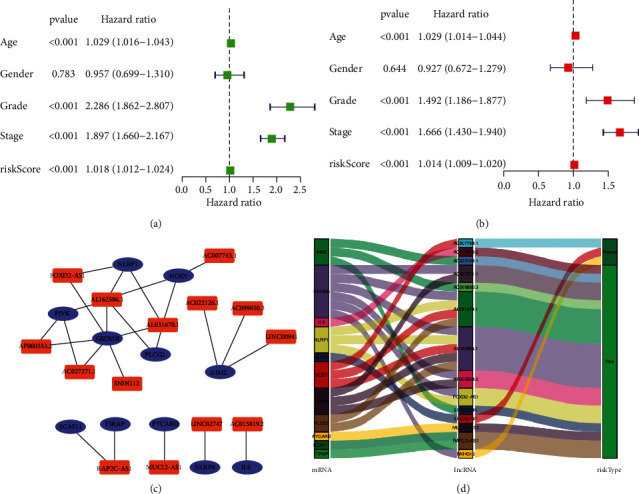
Univariate and multivariate study for the expression of pyroptosis-related lncRNAs. (a) Univariate analysis. (b) Multivariate analysis. (c) The link between mRNA expression and the lncRNA signature. (d) Sankey diagram of the ccRCC lncRNA network.

**Figure 5:**
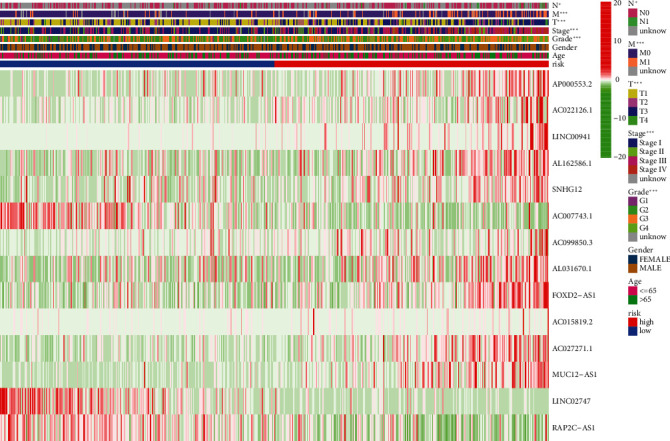
Heat map depicting the predictive signature and clinicopathological manifestations of pyroptosis-related lncRNAs (green: low expression; red: high expression).

**Figure 6:**
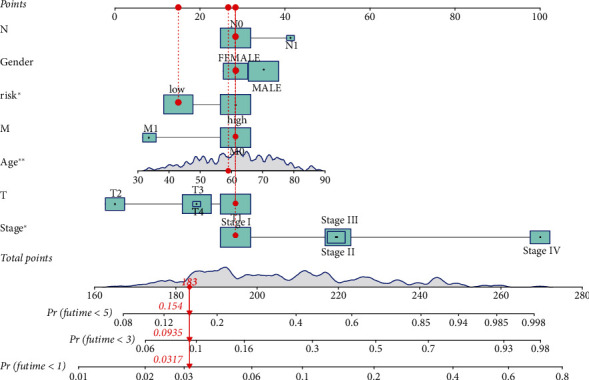
A predictive nomogram in conjunction with both clinicopathological factors and pyroptosis-related lncRNAs.

Low survival rate (*P* < 0.001, Figure 3(a)) was related to the expression of high-risk lncRNA signatures, according to Kaplan–Meier analyses. Meanwhile, the lncRNA signature had an AUC value of 0.771, outperforming conventional clinicopathological characteristics in predicting ccRCC prognosis (Figures 3(b) and 3(e)). When we utilized the patient's risk survival status plot, we observed that the patient's risk score was adversely associated with the survival of patients with ccRCC. According to our heatmap, 11 lncRNAs were favorably connected with our risk model, whereas 3 lncRNAs were negatively connected with our risk model (Figure 3(c)). The AUC predictive value of the lncRNAs signature was 0.791, 0.749, and 0.771 for 1-, 3-, and 5-year survival rates, respectively (Figure 3(d)). Univariate and multivariate Cox analysis demonstrated that the signature of lncRNAs (HR: 1.014, 95% CI: 1.008–1.020), as well as age, grade, and stage, was independent predictors of OS in ccRCC patients (Figures 4(a) and 4(b)).  Figure 4(c) depicts the link between lncRNA and mRNA. A Sankey diagram of the ccRCC lncRNA network is also shown in Figure 4(d). The heat map for the connection between the predictive signature of pyroptosis-related lncRNAs and clinicopathological symptoms was also examined (Figure 5). The hybrid nomogram (Figure 6) including clinicopathological features and the new pyroptosis-related lncRNAs prognostic signature was shown to be stable and accurate, suggesting that it might be utilized in the treatment of ccRCC patients.

### 3.3. GSEA Analyses

**Figure 7:**
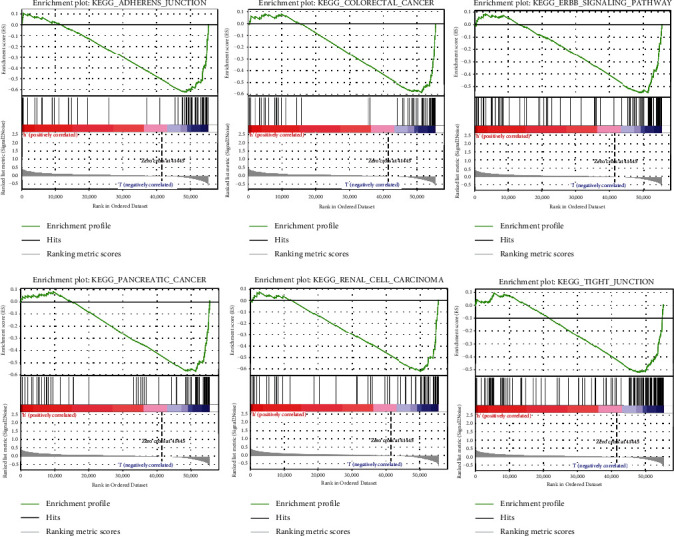
GSEA between low- and high-risk groups.

A majority of the novel pyroptosis-related lncRNAs that were shown to have a prognostic signature affected tumor-related pathways including colorectal cancer, renal cell carcinoma, pancreatic cancer, adherens junction, ERBB signaling pathway, and TIGHT junction, according to GSEA (Figure 7).

### 3.4. Gene Expression and Immune Function

**Figure 8:**
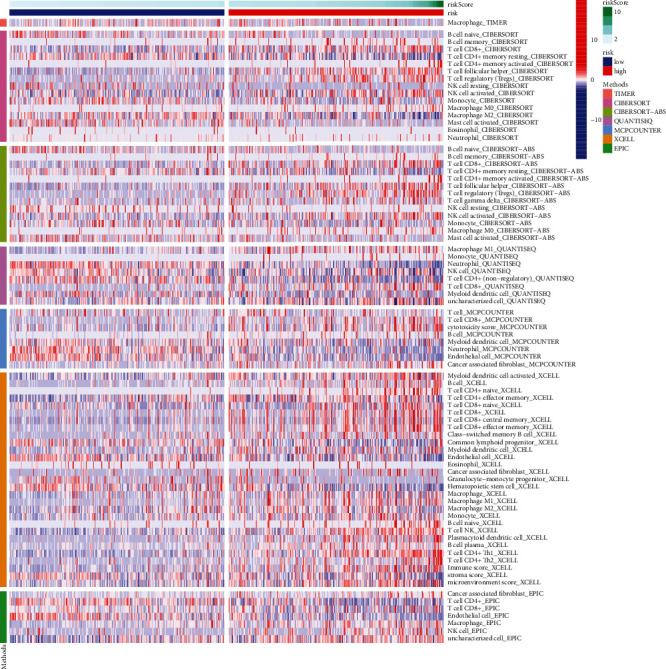
A heat map for immunological responses based on 5 analytic tools between high- and low-risk groups.

**Figure 9:**
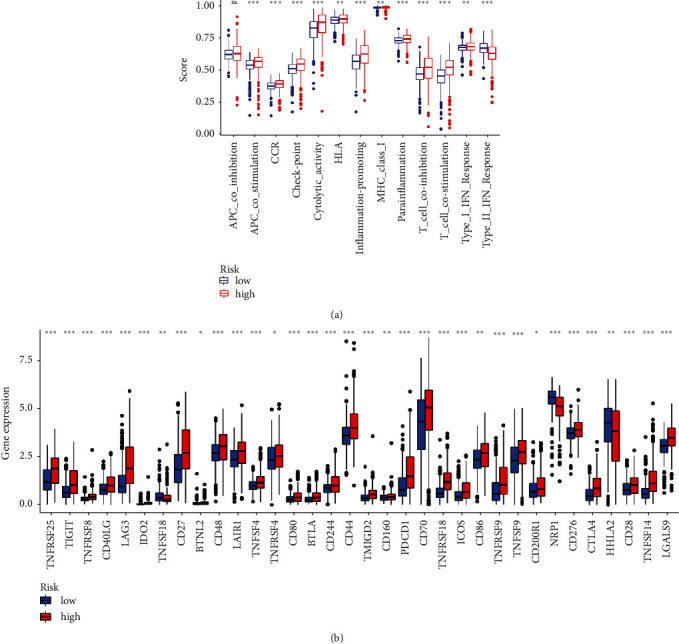
(a) Immune cell subpopulations and associated functions using ssGSEA. (b) Expression of immune checkpoints in high-risk and low-risk individuals.

In Figure 8, we can see the heat map of immunological responses that is based on the aforementioned five analytic tools: CIBERSORT, MCP counter, ESTIMATE, ssGSEA, and TIMER. The presence of multiple immune cell subpopulations was found to correlate with immune functions and relevant activities as determined by the ssGSEA analysis of the TCGA-KIRC dataset. ssGSEA has revealed that, as a result of APC costimulation, CCR, checkpoint, cytolytic activity, HLA, inflammation promoting, MHC class I, parainflammation, T-cell coinhibition, T-cell costimulation, type I IFN response, and type II IFN response were observed to vary considerably between low- and high-risk groups (Figure 9). As the relevance of checkpoint inhibitor-based immunotherapies is the topic of our study, we extended our research to investigate the different expression levels of the immunological checkpoints between the two groups. We detected a large variation in the expression of the majority of immunological checkpoints, such as PDCD1 (PD-1), LAG3, and CTLA4.

### 3.5. Knockdown of FOXD2-AS1 Inhibited KIRC Cell Proliferation and Migration

**Figure 10:**
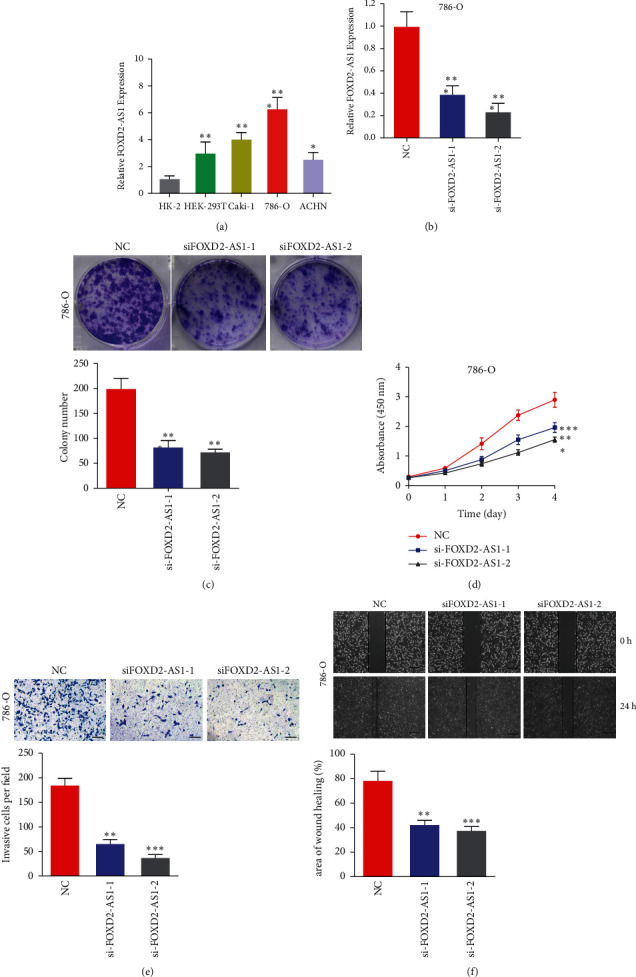
Decreased expression of FOXD2-AS1 inhibits proliferation, invasion, and migration of 786-O cells in vitro. (a) Relative expression of FOXD2-AS1 in five cell lines. (b) qRT-PCR detects the relative silencing levels of FOXD2-AS1 in the 786-O cell line. (c) Images of the colony formation assay after the knockdown of FOXD2-AS1 in the 786-O cell line. (d) CCK-8 assay was applied to detect the efficiency of FOXD2-AS1 knockdown on the proliferation of 786-O cell line. (e) Images of the transwell assay results after the knockdown of FOXD2-AS1 in the 786-O cell line. (f) Representational images of the wound healing assay.

According to the result of correlation analysis between lncRNA and mRNA, we noticed that FOXD2-AS1 was associated with the key pyroptosis-related genes, GSDMB and NLRP1. Through literature review, we found that FOXD2-AS1 plays a role in the development of a variety of tumors [15–17]. However, there is no study related to FOXD2-AS1 in ccRCC. So, we selected FOXD2-AS1 to verify our signature in the next study. We measured the FOXD2-AS1 mRNA levels in ccRCC cell lines (786-O, HEK293 T, Caki-1, ACHN) and normal kidney cell lines (HK-2), and the FOXD2-AS1 mRNA levels were higher in ccRCC cells, where 786-O presented the highest level (Figure 10(a)).  Figure 10(b) indicates the good knockdown efficiency of si-FOXD2-AS1 transfection. According to colony formation experiments, FOXD2-AS1 silencing markedly suppressed ccRCC cell proliferation (Figure 10(c)). Confirming to colony formation experiments, CCK-8 assay revealed that knockdown of FOXD2-AS1 suppressed the proliferation of 786-O cells (Figure 10(d)). We also found that downregulation of FOXD2-AS1 significantly blocked the migration of 786-O cells (Figure 10(e)). The wound healing assay indicated that FOXD2-AS1 knockdown predominantly suppressed metastatic function in 786-O cells (Figure 10(f)).

## 4. Discussion

More recently, scientists have discovered that programmed cell death, referred to as pyroptosis, serves both as a mechanism in tumor formation and a therapeutic component in antitumor strategies [18]. While certain cells in our body are triggered to divide and proliferate due to the production of a high number of inflammatory cytokines during pyroptosis, normal cells are also activated by these inflammatory cytokines, causing them to proliferate and change into tumor cells [7, 19]. On the other hand, promoting tumor cell pyroptosis as a new therapeutic target might be a novel use of tumor pyroptosis. Through a unique technique, we were able to identify a unique pyroptosis-related predictive lncRNA signature based on the TCGA dataset. We first studied the factors involved in the tumor microenvironment. The outcomes of this research revealed a set of possible indicators and targets in the pyroptosis-related signaling pathways.

Using bioinformatics, we were able to determine 14 pyroptosis-related DEGs. KEGG analyzed the gene data further and found that the primary genes in the NOD-like receptor signaling pathway were also present. In a separate study, Liu et al. discovered that the NOD-like receptor family, NLRP3, contributes to the inflammation, pyroptosis, and mucin formation caused by rhinovirus infection in human airway epithelium [20]. A recent research discovered that NOD-like receptor (NLRC5) plays a critical role in ischemia retinopathy, causing microglial pyroptosis, in the formation of retinal ganglion cell death [21]. Overall, in this investigation, 14 lncRNAs with very varied expression patterns were shown to be independent prognostic factors for ccRCC. In a recent research, LINC00941 was shown to enhance pancreatic cancer growth by competing with miR-335-5p to inhibit the ROCK1-mediated LIMK1/cofilin-1 signaling pathway [22]. In gastric cancer, the expression of LINC00941 is increased, which provides a bad prognosis as well as encourages proliferation and metastasis [23]. A separate research looked at SNHG12 lncRNA in hepatocellular carcinoma. Results show that SNHG12 increases tumorigenesis and metastasis by targeting miR-199a/b-5p [24]. In a study published in 2015, Zhou et al. found that lncRNA SNHG12 promotes tumorigenesis and metastasis in osteosarcoma via upregulating Notch2 by directly sequestering miR-195-5p [25]. We verified some of the biological functions of FOXD2-AS1 through in vitro experiments and found that knocking down the expression of FOXD2-AS1 could reduce the proliferation and migration of ccRCC cells. There are no FOXD2-AS1-related studies in ccRCC. Tan et al. found that high expression of FOXD2-AS1 could promote the progression of non-small cell lung cancer through the Wnt/*β*-catenin signaling pathway [15], while Yang et al. found that FOXD2-AS1 could promote the progression of colon cancer by regulating the EMT and Notch signaling pathways [16], and Wang et al. also found that FOXD2-AS1 promoted the progression and proliferation of glioma cells through the FOXD2-AS1/miR-31/CDK1 pathway [26]. Our study found that FOXD2-AS1 is also closely related to GSDMB and NLRP1 by Pearson correlation, which are key genes related to pyroptosis, and whether FOXD2-AS1 can be used as a target to inhibit pyroptosis remains to be further investigated. Here, the varied expression levels of the pyroptosis-associated lncRNAs were clustered into two groups of high- and low-risk in order to study their possible involvement in the development of ccRCC. Pyroptosis may be added to immune checkpoint inhibitors (ICIs) PD-L1 to boost anticancer efficacy [27]. ICI is somewhat unexplored when it comes to pyroptosis (the process by which cells engulfed in pyroptosis undergo apoptosis). It seems that, as the data supporting the role of miRNA and lncRNA in pyroptosis control grows, pyroptosis control becomes more dependent on miRNA and lncRNA. miRNA-214 reduces cellular proliferation and migration in glioma cells by inhibiting the expression of caspase 1, a protein that is implicated in pyroptosis [28]. Micro-RNA-30d controls pyroptosis in diabetic cardiomyopathy by targeting foxo3a, which is the molecule necessary for pyroptosis to take place [29]. lncRNAs are responsible for pathological processes that are associated with different illnesses, including cardiovascular disorders, through regulation of pyroptosis signaling pathway-related proteins by miRNAs, which is especially relevant in this disease class. Importantly, numerous lncRNAs, including MALAT1 and KCNQ1, and by serving as miRNA biosynthesis factors, Neat1 has been demonstrated to have a vital function in inducing pyroptosis. A similar technique of control is also seen in several other disorders such as cancer, renal disease, and autoimmune illnesses. Tolerogenic dendritic cells are critical to a successful T-cell response, and this sets up an environment for tolerance to develop [30]. By undertaking miR-3076-3p, Neat1 promotes NLRP3 inflammasome production, resulting in a tolerogenic phenotype, which acts as a sponge for the miR-3076-3p sponge. In addition, it has been postulated that there may be a biological mechanism involved in calcium oxalate-induced kidney stones. In renal tubular epithelial cells, lncRNA00339 promotes pyroptosis through the miR223p/NLRP3 axis [31]. In this way, the findings from these research studies demonstrate that lncRNAs are found to be upregulated in disorders related to pyroptosis, and this has crucial implications for the development of novel pharmacological therapies.

This novel kind of programmed cell death which occurs in response to inflammation has also been reported in several cell lines, and it is thought to be a prominent pathophysiological mechanism in many disorders [32, 33]. Yet, there are a number of important aspects that remain unaddressed, for example, the link between pyroptosis, immunogenicity, and other cell deaths. Thus, this research examined biomarkers for pyroptosis that may be used to predict the prognosis of ccRCC, which helps guide the therapy options for the illness. Still, our signature profiles require additional confirmation by trying them on diverse populations. The general reliability of our results cannot be guaranteed since our findings were not validated using clinical samples. Thus, according to the clinical data we have, our results should be taken with a grain of salt. While more validation is clearly needed, the model shown in this work generally serves as a useful predictive tool.

## 5. Conclusion

Specific pyroptosis-associated lncRNAs signature may help to predict the prognosis of ccRCC.

## Data Availability

The datasets analyzed in this study are available in The Cancer Genome Atlas (TCGA). TCGA belongs to the public dataset. Users can download relevant data for free for research and publish relevant articles.

## Abbreviations

ccRCC: Clear cell renal cell carcinoma
miRNA: Micro-RNA
OS: Overall survival
DEGs: Differentially expressed genes
TCGA: The Cancer Genome Atlas
GO: Gene Ontology
BP: Biological processes
MF: Molecular function
CC: Cellular components
KEGG: Kyoto Encyclopedia of Genes and Genomes
GSEA: Gene set enrichment analyses
FDR: False discovery rate
ssGSEA: Single-sample gene set enrichment analysis (ssGSEA).

## Supplementary Materials

Supplementary Materials
*Supplementary File Table S1*. Patients' clinical features from the TCGA dataset. *Supplementary File Table S2*. 33 pyroptosis-related genes. *Supplementary File Table S3*. The sequences of primers and siRNAs used in this study. *Supplementary File Table S4.* 14 pyroptosis-related DEGs from TCGA-KIRC. *Supplementary File Table S5*. 1042 pyroptosis-related lncRNAs. *Supplementary File Table S6*. 299 significant pyroptosis-related lncRNAs after univariate Cox analysis. *Supplementary File Figure S1.* 14 pyroptosis-related DEGs from TCGA-KIRC.
