# Supplementary material for: A Novel Pyroptosis-Related lncRNAs Signature for Predicting the Prognosis of Kidney Renal Clear Cell Carcinoma and Its Associations with Immunity
Source: J Oncol. 2021 Oct 18;2021:9997185. doi: 10.1155/2021/9997185 (PMC8577956; doi:10.1155/2021/9997185)
Supplement: Supplementary Materials — Supplementary File Table S1. Patients' clinical features from the TCGA dataset. Supplementary File Table S2. 33 pyroptosis-related genes. Supplementary File Table S3. The sequences of primers and siRNAs used in this study. Supplementary File Table S4. 14 pyroptosis-related DEGs from TCGA-KIRC. Supplementary File Table S5. 1042 pyroptosis-related lncRNAs. Supplementary File Table S6. 299 significant pyroptosis-related lncRNAs after univariate Cox analysis. Supplementary File Figure S1. 14 pyroptosis-related DEGs from TCGA-KIRC. [file 9997185.f1.zip › 9997185.f1/Table S1 (1).docx]

Table S1. Patients' clinical features from the TCGA dataset.

| **Variable** | **Number of samples** |
| --- | --- |
| **Gender** |  |
| Male | 346 |
| Female | 191 |
| **Age at diagnosis** |  |
| ≤65 | 352 |
| ≥65 | 185 |
| **Grade** |  |
| G1 | 14 |
| G2 | 230 |
| G3 | 207 |
| G4 | 78 |
| NA | 8 |
| **Stage** |  |
| Stage I | 269 |
| Stage II | 57 |
| Stage III | 125 |
| Stage IV | 83 |
| NA | 3 |
| **T** |  |
| T1 | 275 |
| T2 | 69 |
| T3 | 182 |
| T4 | 11 |
| **M** |  |
| M0 | 426 |
| M1 | 79 |
| NA | 32 |
| **N** |  |
| N0 | 240 |
| N1 | 17 |
| NA | 280 |
