# Supplementary material for: A Novel Pyroptosis-Related lncRNAs Signature for Predicting the Prognosis of Kidney Renal Clear Cell Carcinoma and Its Associations with Immunity
Source: J Oncol. 2021 Oct 18;2021:9997185. doi: 10.1155/2021/9997185 (PMC8577956; doi:10.1155/2021/9997185)
Supplement: Supplementary Materials — Supplementary File Table S1. Patients' clinical features from the TCGA dataset. Supplementary File Table S2. 33 pyroptosis-related genes. Supplementary File Table S3. The sequences of primers and siRNAs used in this study. Supplementary File Table S4. 14 pyroptosis-related DEGs from TCGA-KIRC. Supplementary File Table S5. 1042 pyroptosis-related lncRNAs. Supplementary File Table S6. 299 significant pyroptosis-related lncRNAs after univariate Cox analysis. Supplementary File Figure S1. 14 pyroptosis-related DEGs from TCGA-KIRC. [file 9997185.f1.zip › 9997185.f1/Table S2 (1).docx]

**Table S2:** 33 pyroptosis-related genes

AIM2

CASP1

CASP3

CASP4

CASP5

CASP6

CASP8

CASP9

ELANE

GPX4

GSDMA

GSDMB

GSDMC

GSDMD

GSDME

IL18

IL1B

IL6

NLRC4

NLRP1

NLRP2

NLRP3

NLRP6

NLRP7

NOD1

NOD2

PJVK

PLCG1

PRKACA

PYCARD

SCAF11

TIRAP

TNF
