# Supplementary material for: A Novel Pyroptosis-Related lncRNAs Signature for Predicting the Prognosis of Kidney Renal Clear Cell Carcinoma and Its Associations with Immunity
Source: J Oncol. 2021 Oct 18;2021:9997185. doi: 10.1155/2021/9997185 (PMC8577956; doi:10.1155/2021/9997185)
Supplement: Supplementary Materials — Supplementary File Table S1. Patients' clinical features from the TCGA dataset. Supplementary File Table S2. 33 pyroptosis-related genes. Supplementary File Table S3. The sequences of primers and siRNAs used in this study. Supplementary File Table S4. 14 pyroptosis-related DEGs from TCGA-KIRC. Supplementary File Table S5. 1042 pyroptosis-related lncRNAs. Supplementary File Table S6. 299 significant pyroptosis-related lncRNAs after univariate Cox analysis. Supplementary File Figure S1. 14 pyroptosis-related DEGs from TCGA-KIRC. [file 9997185.f1.zip › 9997185.f1/Table S4 (1).docx]

**Table S4:** 14 pyroptosis-related DEGs from TCGA-KIRC

| gene | conMean | treatMean | logFC | pValue | fdr |
| --- | --- | --- | --- | --- | --- |
| AIM2 | 0.159185 | 1.324488 | 3.056658 | 3.08E-28 | 1.32E-27 |
| CASP1 | 2.518214 | 8.464013 | 1.748941 | 2.10E-33 | 3.14E-32 |
| CASP4 | 3.514194 | 8.346382 | 1.247957 | 3.45E-34 | 1.04E-32 |
| CASP5 | 0.038712 | 0.301187 | 2.959821 | 3.19E-32 | 3.19E-31 |
| GSDMA | 0.174059 | 0.478997 | 1.460443 | 1.34E-09 | 1.83E-09 |
| GSDMB | 0.363561 | 1.345898 | 1.8883 | 6.04E-18 | 1.07E-17 |
| GSDMC | 0.027824 | 0.122988 | 2.144092 | 5.10E-12 | 8.49E-12 |
| NLRC4 | 0.380052 | 1.354478 | 1.833468 | 1.00E-31 | 7.53E-31 |
| NLRP1 | 1.135399 | 2.67427 | 1.235946 | 1.91E-20 | 4.41E-20 |
| NLRP2 | 1.264828 | 0.395417 | -1.67749 | 7.00E-21 | 1.91E-20 |
| NLRP3 | 0.569212 | 1.490087 | 1.388359 | 2.17E-20 | 4.65E-20 |
| NLRP6 | 0.6149 | 1.70463 | 1.471036 | 4.05E-10 | 5.79E-10 |
| NLRP7 | 0.012359 | 0.057764 | 2.224669 | 7.58E-12 | 1.14E-11 |
| NOD2 | 0.16621 | 0.927549 | 2.480416 | 2.52E-31 | 1.51E-30 |
| PYCARD | 2.459118 | 9.994205 | 2.022951 | 1.14E-30 | 5.70E-30 |
