# Supplementary material for: A Novel Pyroptosis-Related lncRNAs Signature for Predicting the Prognosis of Kidney Renal Clear Cell Carcinoma and Its Associations with Immunity
Source: J Oncol. 2021 Oct 18;2021:9997185. doi: 10.1155/2021/9997185 (PMC8577956; doi:10.1155/2021/9997185)
Supplement: Supplementary Materials — Supplementary File Table S1. Patients' clinical features from the TCGA dataset. Supplementary File Table S2. 33 pyroptosis-related genes. Supplementary File Table S3. The sequences of primers and siRNAs used in this study. Supplementary File Table S4. 14 pyroptosis-related DEGs from TCGA-KIRC. Supplementary File Table S5. 1042 pyroptosis-related lncRNAs. Supplementary File Table S6. 299 significant pyroptosis-related lncRNAs after univariate Cox analysis. Supplementary File Figure S1. 14 pyroptosis-related DEGs from TCGA-KIRC. [file 9997185.f1.zip › 9997185.f1/Table S5 (1).docx]

**Table S5:** 1042 pyroptosis-related lncRNAs.

| ferrGene | lncRNA | cor | pvalue | Regulation |
| --- | --- | --- | --- | --- |
| SCAF11 | AC007406.4 | 0.66691471 | 1.26E-70 | postive |
| GSDMB | AL139287.1 | 0.65512567 | 2.17E-67 | postive |
| NLRP1 | AL139287.1 | 0.67162659 | 5.84E-72 | postive |
| NOD1 | AL139287.1 | 0.52753728 | 6.05E-40 | postive |
| PLCG1 | AL139287.1 | 0.57645095 | 4.73E-49 | postive |
| SCAF11 | AC007376.2 | 0.54097785 | 2.67E-42 | postive |
| GSDMB | AL022328.2 | 0.70009327 | 1.39E-80 | postive |
| NLRP1 | AL022328.2 | 0.67047306 | 1.25E-71 | postive |
| PJVK | AL022328.2 | 0.51561857 | 6.07E-38 | postive |
| PLCG1 | AL022328.2 | 0.54877699 | 1.03E-43 | postive |
| SCAF11 | AC004884.2 | 0.51415424 | 1.06E-37 | postive |
| GSDMB | AC008875.1 | 0.68917973 | 3.69E-77 | postive |
| NLRP1 | AC008875.1 | 0.52333208 | 3.14E-39 | postive |
| PJVK | AC008875.1 | 0.55864688 | 1.46E-45 | postive |
| GSDMB | AC002553.1 | 0.65204372 | 1.44E-66 | postive |
| NLRP1 | AC002553.1 | 0.52851713 | 4.11E-40 | postive |
| PJVK | AC002553.1 | 0.52213269 | 5.00E-39 | postive |
| GSDMB | AC025287.3 | 0.54497715 | 5.08E-43 | postive |
| NLRP1 | AC025287.3 | 0.52007475 | 1.11E-38 | postive |
| SCAF11 | AC009318.3 | 0.51530625 | 6.84E-38 | postive |
| IL18 | MMEL1-AS1 | 0.53635458 | 1.78E-41 | postive |
| GSDMB | LINC01772 | 0.63050113 | 4.45E-61 | postive |
| NLRP1 | LINC01772 | 0.60303682 | 1.11E-54 | postive |
| NOD1 | LINC01772 | 0.53636438 | 1.77E-41 | postive |
| PJVK | LINC01772 | 0.50423481 | 4.19E-36 | postive |
| PLCG1 | LINC01772 | 0.51098597 | 3.47E-37 | postive |
| SCAF11 | AP001432.1 | 0.52466895 | 1.87E-39 | postive |
| GSDMB | AL355488.1 | 0.69667528 | 1.71E-79 | postive |
| NLRP1 | AL355488.1 | 0.51046223 | 4.22E-37 | postive |
| PJVK | AL355488.1 | 0.68071581 | 1.32E-74 | postive |
| GSDMB | MHENCR | 0.73326118 | 5.13E-92 | postive |
| PJVK | MHENCR | 0.65895663 | 2.00E-68 | postive |
| GSDMB | LINC01389 | 0.58327097 | 1.91E-50 | postive |
| PJVK | LINC01389 | 0.50370528 | 5.09E-36 | postive |
| GSDMB | AC079684.1 | 0.6210454 | 8.38E-59 | postive |
| PJVK | AC079684.1 | 0.55718816 | 2.77E-45 | postive |
| GSDMB | AC132192.2 | 0.63550671 | 2.58E-62 | postive |
| PJVK | AC132192.2 | 0.64501396 | 9.98E-65 | postive |
| GSDMB | AC005393.1 | 0.62783165 | 1.99E-60 | postive |
| PJVK | AC005393.1 | 0.55181709 | 2.82E-44 | postive |
| SCAF11 | AC026470.2 | 0.52476746 | 1.80E-39 | postive |
| NLRP1 | AC093110.1 | 0.54649011 | 2.69E-43 | postive |
| NOD1 | AC093110.1 | 0.53120989 | 1.41E-40 | postive |
| PLCG1 | AC093110.1 | 0.51868422 | 1.89E-38 | postive |
| GSDMB | AL158834.2 | 0.66041267 | 8.02E-69 | postive |
| NLRP1 | AL158834.2 | 0.52880398 | 3.67E-40 | postive |
| PJVK | AL158834.2 | 0.54820371 | 1.31E-43 | postive |
| SCAF11 | AC009716.1 | 0.51505753 | 7.51E-38 | postive |
| GSDMB | LINC00926 | 0.73610429 | 4.46E-93 | postive |
| NLRP1 | LINC00926 | 0.69680054 | 1.56E-79 | postive |
| PLCG1 | LINC00926 | 0.50334243 | 5.81E-36 | postive |
| GSDMB | AC022150.2 | 0.59981843 | 5.71E-54 | postive |
| NLRP1 | AC022150.2 | 0.62524338 | 8.37E-60 | postive |
| NOD1 | AC022150.2 | 0.52186588 | 5.55E-39 | postive |
| PLCG1 | AC022150.2 | 0.61752405 | 5.63E-58 | postive |
| NOD1 | AL139011.1 | 0.50229163 | 8.51E-36 | postive |
| GSDMB | AC109460.1 | 0.5360065 | 2.04E-41 | postive |
| GSDMB | AC138028.4 | 0.63955725 | 2.48E-63 | postive |
| NLRP1 | AC138028.4 | 0.70049508 | 1.03E-80 | postive |
| NOD1 | AC138028.4 | 0.59060532 | 5.54E-52 | postive |
| PLCG1 | AC138028.4 | 0.61866644 | 3.04E-58 | postive |
| IL18 | AP000787.1 | 0.53036073 | 1.98E-40 | postive |
| SCAF11 | AP001033.2 | 0.51947461 | 1.39E-38 | postive |
| IL18 | AC016866.1 | 0.51019628 | 4.66E-37 | postive |
| SCAF11 | AP000786.1 | 0.57733882 | 3.13E-49 | postive |
| PJVK | AC020765.2 | 0.53879137 | 6.57E-42 | postive |
| GSDMB | AC245884.8 | 0.73231135 | 1.15E-91 | postive |
| NLRP1 | AC245884.8 | 0.50416998 | 4.29E-36 | postive |
| PJVK | AC245884.8 | 0.65241366 | 1.15E-66 | postive |
| GSDMB | AC011472.1 | 0.67190323 | 4.87E-72 | postive |
| NLRP1 | AC011472.1 | 0.70504578 | 3.45E-82 | postive |
| NOD1 | AC011472.1 | 0.53472486 | 3.43E-41 | postive |
| PLCG1 | AC011472.1 | 0.6461004 | 5.23E-65 | postive |
| IL18 | AC138305.1 | 0.63813932 | 5.65E-63 | postive |
| PJVK | AC005962.1 | 0.54035432 | 3.46E-42 | postive |
| SCAF11 | AL731566.2 | 0.50578704 | 2.38E-36 | postive |
| SCAF11 | RBM26-AS1 | 0.52016665 | 1.07E-38 | postive |
| GSDMB | CCDC18-AS1 | 0.78899287 | 1.09E-115 | postive |
| NLRP1 | CCDC18-AS1 | 0.63351419 | 8.07E-62 | postive |
| PJVK | CCDC18-AS1 | 0.56787459 | 2.42E-47 | postive |
| GSDMB | AL021707.4 | 0.54584564 | 3.53E-43 | postive |
| GSDMB | AL022322.1 | 0.68640072 | 2.60E-76 | postive |
| NLRP1 | AL022322.1 | 0.5978282 | 1.55E-53 | postive |
| PJVK | AL022322.1 | 0.55639441 | 3.91E-45 | postive |
| PLCG1 | AL022322.1 | 0.51411835 | 1.07E-37 | postive |
| GSDMB | COL4A2-AS1 | 0.53074465 | 1.70E-40 | postive |
| NLRP1 | COL4A2-AS1 | 0.66361985 | 1.05E-69 | postive |
| NOD1 | COL4A2-AS1 | 0.61803235 | 4.28E-58 | postive |
| PLCG1 | COL4A2-AS1 | 0.61798088 | 4.40E-58 | postive |
| PJVK | AC121761.2 | 0.54187646 | 1.85E-42 | postive |
| NOD1 | AC012531.1 | 0.52266102 | 4.08E-39 | postive |
| GSDMB | XPC-AS1 | 0.54742461 | 1.82E-43 | postive |
| NLRP1 | XPC-AS1 | 0.52695282 | 7.62E-40 | postive |
| NOD1 | XPC-AS1 | 0.52815581 | 4.74E-40 | postive |
| SCAF11 | AC068790.7 | 0.51224322 | 2.17E-37 | postive |
| GSDMB | AL606760.2 | 0.61047731 | 2.37E-56 | postive |
| SCAF11 | AC093423.2 | 0.50098631 | 1.36E-35 | postive |
| GSDMB | LINC01176 | 0.70245659 | 2.41E-81 | postive |
| NLRP1 | LINC01176 | 0.56747976 | 2.89E-47 | postive |
| PJVK | LINC01176 | 0.56811072 | 2.17E-47 | postive |
| SCAF11 | AC026124.2 | 0.50111959 | 1.30E-35 | postive |
| GSDMB | AC116667.1 | 0.64389266 | 1.94E-64 | postive |
| NLRP1 | AC116667.1 | 0.63639709 | 1.55E-62 | postive |
| PJVK | AC116667.1 | 0.55847975 | 1.58E-45 | postive |
| NOD1 | AC004918.3 | 0.56367657 | 1.59E-46 | postive |
| SCAF11 | AC004918.3 | 0.5734844 | 1.87E-48 | postive |
| GSDMB | GABPB1-AS1 | 0.54260156 | 1.37E-42 | postive |
| GSDMB | AL353622.1 | 0.61135892 | 1.49E-56 | postive |
| PJVK | AL353622.1 | 0.53993623 | 4.11E-42 | postive |
| NOD1 | SLC6A1-AS1 | 0.534501 | 3.76E-41 | postive |
| NLRP6 | LINC02754 | 0.51381995 | 1.20E-37 | postive |
| GSDMB | AC092809.4 | 0.54516384 | 4.70E-43 | postive |
| GSDMB | AC005519.1 | 0.6214319 | 6.79E-59 | postive |
| NLRP1 | AC005519.1 | 0.59429603 | 9.02E-53 | postive |
| NOD1 | AC005519.1 | 0.5247287 | 1.82E-39 | postive |
| PLCG1 | AC005519.1 | 0.56523909 | 7.91E-47 | postive |
| GSDMB | AL031717.1 | 0.57488647 | 9.78E-49 | postive |
| NLRP1 | AL031717.1 | 0.50597407 | 2.22E-36 | postive |
| PJVK | FOXO6-AS1 | 0.55253916 | 2.07E-44 | postive |
| GSDMB | PAN3-AS1 | 0.54687296 | 2.29E-43 | postive |
| NLRP1 | PAN3-AS1 | 0.54350377 | 9.39E-43 | postive |
| PJVK | PAN3-AS1 | 0.6115743 | 1.33E-56 | postive |
| SCAF11 | AC010261.2 | 0.53428736 | 4.10E-41 | postive |
| GPX4 | CH17-340M24.3 | 0.50254137 | 7.77E-36 | postive |
| GSDMB | AC009133.1 | 0.6364255 | 1.52E-62 | postive |
| PJVK | AC007220.1 | 0.56061877 | 6.16E-46 | postive |
| GSDMB | AC116913.1 | 0.65677452 | 7.83E-68 | postive |
| NLRP1 | AC116913.1 | 0.56427215 | 1.22E-46 | postive |
| PJVK | AC116913.1 | 0.54889644 | 9.76E-44 | postive |
| GSDMB | AC022762.2 | 0.65469404 | 2.84E-67 | postive |
| PJVK | AC022762.2 | 0.61928262 | 2.18E-58 | postive |
| GSDMB | HPN-AS1 | 0.51426667 | 1.01E-37 | postive |
| GSDMB | AC010168.2 | 0.51548428 | 6.39E-38 | postive |
| GSDMB | AC008115.3 | 0.53598849 | 2.06E-41 | postive |
| NLRP1 | AC008115.3 | 0.52663391 | 8.64E-40 | postive |
| NOD1 | AC008115.3 | 0.52239233 | 4.53E-39 | postive |
| GSDMB | LINC00174 | 0.81085316 | 4.89E-127 | postive |
| NLRP1 | LINC00174 | 0.61848918 | 3.35E-58 | postive |
| PJVK | LINC00174 | 0.62884344 | 1.13E-60 | postive |
| GSDMB | AC009283.1 | 0.75631846 | 4.96E-101 | postive |
| NLRP1 | AC009283.1 | 0.67436392 | 9.53E-73 | postive |
| PJVK | AC009283.1 | 0.69159694 | 6.64E-78 | postive |
| PLCG1 | AC009283.1 | 0.52594831 | 1.13E-39 | postive |
| IL18 | GAS6-DT | 0.58939575 | 1.00E-51 | postive |
| GSDMB | AC010976.1 | 0.50345092 | 5.58E-36 | postive |
| GSDMB | AC006272.1 | 0.61795597 | 4.46E-58 | postive |
| NLRP1 | AC006272.1 | 0.57121107 | 5.30E-48 | postive |
| GSDMB | AL049840.5 | 0.56271873 | 2.43E-46 | postive |
| NLRP1 | AL049840.5 | 0.64306071 | 3.18E-64 | postive |
| NOD1 | AL049840.5 | 0.54424451 | 6.90E-43 | postive |
| PLCG1 | AL049840.5 | 0.52333545 | 3.14E-39 | postive |
| GSDMB | AL590617.2 | 0.5469782 | 2.19E-43 | postive |
| NLRP1 | AL590617.2 | 0.53629101 | 1.82E-41 | postive |
| PJVK | AL590617.2 | 0.53438846 | 3.93E-41 | postive |
| GSDMB | GARS1-DT | 0.62887833 | 1.11E-60 | postive |
| NLRP1 | GARS1-DT | 0.505113 | 3.04E-36 | postive |
| PJVK | GARS1-DT | 0.54930418 | 8.21E-44 | postive |
| GSDMB | NARF-IT1 | 0.65125498 | 2.34E-66 | postive |
| NLRP1 | NARF-IT1 | 0.59102314 | 4.52E-52 | postive |
| NOD1 | NARF-IT1 | 0.55628496 | 4.10E-45 | postive |
| PLCG1 | NARF-IT1 | 0.51324013 | 1.49E-37 | postive |
| SCAF11 | AC016586.1 | 0.51125866 | 3.13E-37 | postive |
| GSDMB | LINC02615 | 0.52863396 | 3.92E-40 | postive |
| PJVK | LINC02615 | 0.54391515 | 7.92E-43 | postive |
| GSDMB | AC011479.2 | 0.55914352 | 1.18E-45 | postive |
| NLRP1 | AL049871.1 | 0.51324074 | 1.49E-37 | postive |
| NOD1 | AL049871.1 | 0.55750878 | 2.41E-45 | postive |
| PLCG1 | AL049871.1 | 0.55370369 | 1.25E-44 | postive |
| SCAF11 | AL353804.1 | 0.57713882 | 3.44E-49 | postive |
| GSDMB | AC009107.2 | 0.61002084 | 3.01E-56 | postive |
| NLRP1 | AC009107.2 | 0.53467213 | 3.51E-41 | postive |
| PLCG1 | AC009107.2 | 0.50791165 | 1.09E-36 | postive |
| GSDMB | AC107081.1 | 0.68116414 | 9.70E-75 | postive |
| PJVK | AC107081.1 | 0.63012495 | 5.50E-61 | postive |
| CASP1 | AL135818.1 | 0.55096405 | 4.05E-44 | postive |
| NOD2 | AL135818.1 | 0.62923471 | 9.07E-61 | postive |
| GSDMB | AC127502.2 | 0.70814641 | 3.28E-83 | postive |
| NLRP1 | AC127502.2 | 0.56532165 | 7.62E-47 | postive |
| PJVK | AC127502.2 | 0.54296099 | 1.18E-42 | postive |
| SCAF11 | AC068790.2 | 0.55891716 | 1.30E-45 | postive |
| SCAF11 | PSMD6-AS2 | 0.58057624 | 6.85E-50 | postive |
| GSDMB | AP000919.3 | 0.55691997 | 3.11E-45 | postive |
| IL18 | PLBD1-AS1 | 0.50499171 | 3.18E-36 | postive |
| SCAF11 | AC073073.2 | 0.57551417 | 7.32E-49 | postive |
| GSDMB | AL158151.4 | 0.62260724 | 3.57E-59 | postive |
| GSDMB | AC009690.2 | 0.7477656 | 1.42E-97 | postive |
| NLRP1 | AC009690.2 | 0.56460374 | 1.05E-46 | postive |
| PJVK | AC009690.2 | 0.63662188 | 1.36E-62 | postive |
| GSDMB | AC004253.1 | 0.74161464 | 3.59E-95 | postive |
| NLRP1 | AC004253.1 | 0.59883082 | 9.39E-54 | postive |
| PJVK | AC004253.1 | 0.60838212 | 7.08E-56 | postive |
| GSDMB | AC012368.1 | 0.54987691 | 6.44E-44 | postive |
| PJVK | AC012368.1 | 0.59265739 | 2.03E-52 | postive |
| SCAF11 | AC118344.1 | 0.51639651 | 4.52E-38 | postive |
| GSDMB | AP001010.1 | 0.62536486 | 7.83E-60 | postive |
| NLRP1 | AP001010.1 | 0.53718937 | 1.26E-41 | postive |
| GSDMA | AC002091.2 | 0.55386488 | 1.17E-44 | postive |
| NLRC4 | AC002091.2 | 0.62361303 | 2.06E-59 | postive |
| NLRP3 | AC002091.2 | 0.55661163 | 3.56E-45 | postive |
| GSDMB | LINC01238 | 0.56896176 | 1.48E-47 | postive |
| GSDMB | AC005046.1 | 0.5686734 | 1.68E-47 | postive |
| PJVK | AC005046.1 | 0.51321722 | 1.50E-37 | postive |
| GSDMB | AL606534.1 | 0.52112368 | 7.39E-39 | postive |
| GSDMB | AC132938.1 | 0.54656794 | 2.61E-43 | postive |
| IL18 | AL356740.1 | 0.51059535 | 4.01E-37 | postive |
| GSDMB | RNF213-AS1 | 0.51494838 | 7.83E-38 | postive |
| SCAF11 | AC004943.1 | 0.54539247 | 4.27E-43 | postive |
| GSDMB | AC008735.1 | 0.63020611 | 5.25E-61 | postive |
| NLRP1 | AC008735.1 | 0.53929236 | 5.35E-42 | postive |
| GSDMB | AC013403.2 | 0.53145928 | 1.28E-40 | postive |
| PJVK | AC013403.2 | 0.50787829 | 1.10E-36 | postive |
| GSDMB | AC007991.4 | 0.52890968 | 3.52E-40 | postive |
| SCAF11 | AC011825.2 | 0.51604977 | 5.16E-38 | postive |
| GSDMB | AC037459.2 | 0.58614991 | 4.81E-51 | postive |
| NLRP1 | AC037459.2 | 0.63687399 | 1.17E-62 | postive |
| NOD1 | AC037459.2 | 0.57364109 | 1.74E-48 | postive |
| PLCG1 | AC037459.2 | 0.60361308 | 8.30E-55 | postive |
| GSDMB | AL158063.1 | 0.55556161 | 5.62E-45 | postive |
| PJVK | AL158063.1 | 0.59928981 | 7.45E-54 | postive |
| SCAF11 | ACAP2-IT1 | 0.59094914 | 4.68E-52 | postive |
| NLRP1 | BX255925.1 | 0.55686173 | 3.19E-45 | postive |
| PJVK | AC109322.1 | 0.52365753 | 2.77E-39 | postive |
| GSDMB | AP001107.1 | 0.5470222 | 2.15E-43 | postive |
| SCAF11 | LINC02175 | 0.52718423 | 6.96E-40 | postive |
| TIRAP | LINC02175 | 0.5031894 | 6.14E-36 | postive |
| GSDMB | AC007566.1 | 0.58627912 | 4.52E-51 | postive |
| NLRP1 | AC007566.1 | 0.53661053 | 1.60E-41 | postive |
| NOD1 | AC007566.1 | 0.51558379 | 6.15E-38 | postive |
| PJVK | AC007566.1 | 0.55859214 | 1.50E-45 | postive |
| GSDMB | AC087500.2 | 0.61649204 | 9.79E-58 | postive |
| GSDMB | AC135050.3 | 0.76573192 | 5.24E-105 | postive |
| NLRP1 | AC135050.3 | 0.53052926 | 1.85E-40 | postive |
| PJVK | AC135050.3 | 0.60074846 | 3.57E-54 | postive |
| IL18 | Z82185.1 | 0.60445326 | 5.39E-55 | postive |
| GSDMB | AL365330.1 | 0.63588188 | 2.08E-62 | postive |
| NLRP1 | AL365330.1 | 0.63648168 | 1.47E-62 | postive |
| PLCG1 | AL365330.1 | 0.53195157 | 1.05E-40 | postive |
| GSDMB | RNF139-AS1 | 0.76018629 | 1.21E-102 | postive |
| NLRP1 | RNF139-AS1 | 0.54927698 | 8.31E-44 | postive |
| PJVK | RNF139-AS1 | 0.63793274 | 6.37E-63 | postive |
| GSDMB | AC048341.2 | 0.72225962 | 4.86E-88 | postive |
| NLRP1 | AC048341.2 | 0.51408384 | 1.09E-37 | postive |
| PJVK | AC048341.2 | 0.66848806 | 4.55E-71 | postive |
| GSDMB | AC112484.1 | 0.53385528 | 4.88E-41 | postive |
| GSDMB | AC008969.1 | 0.51191476 | 2.45E-37 | postive |
| NLRP1 | AC008969.1 | 0.54900277 | 9.33E-44 | postive |
| NOD1 | AC008969.1 | 0.51006288 | 4.89E-37 | postive |
| GSDMB | FAM13A-AS1 | 0.63652357 | 1.44E-62 | postive |
| NLRP1 | FAM13A-AS1 | 0.52505901 | 1.60E-39 | postive |
| PJVK | FAM13A-AS1 | 0.56022147 | 7.34E-46 | postive |
| GSDMB | SNHG4 | 0.57470617 | 1.06E-48 | postive |
| GSDMB | THBS3-AS1 | 0.75080356 | 8.74E-99 | postive |
| NLRP1 | THBS3-AS1 | 0.58224017 | 3.12E-50 | postive |
| PJVK | THBS3-AS1 | 0.53480537 | 3.33E-41 | postive |
| GSDMB | AC010201.2 | 0.56838454 | 1.92E-47 | postive |
| PJVK | AC010201.2 | 0.53411428 | 4.39E-41 | postive |
| GSDMB | AL512306.2 | 0.55232478 | 2.27E-44 | postive |
| GSDMB | ARRDC1-AS1 | 0.55952388 | 9.97E-46 | postive |
| GSDMB | AC015802.5 | 0.61069131 | 2.12E-56 | postive |
| PJVK | AC015802.5 | 0.63020157 | 5.27E-61 | postive |
| GSDMB | AL136295.2 | 0.68787875 | 9.23E-77 | postive |
| NLRP1 | AL136295.2 | 0.51427244 | 1.01E-37 | postive |
| PJVK | AL136295.2 | 0.56462535 | 1.04E-46 | postive |
| GSDMB | NDUFA6-DT | 0.53188798 | 1.07E-40 | postive |
| SCAF11 | AC016590.2 | 0.57156398 | 4.51E-48 | postive |
| SCAF11 | AC005740.3 | 0.50850467 | 8.73E-37 | postive |
| PYCARD | LINC00623 | 0.51318865 | 1.52E-37 | postive |
| SCAF11 | AC016747.3 | 0.52488588 | 1.71E-39 | postive |
| IL18 | AC120498.4 | 0.51029974 | 4.48E-37 | postive |
| GSDMB | AL139286.1 | 0.54254888 | 1.40E-42 | postive |
| PJVK | AL139286.1 | 0.51662699 | 4.14E-38 | postive |
| GSDMB | AL161729.4 | 0.53891284 | 6.25E-42 | postive |
| PJVK | AL161729.4 | 0.54422445 | 6.96E-43 | postive |
| GSDMB | LINC01089 | 0.75705184 | 2.47E-101 | postive |
| NLRP1 | LINC01089 | 0.58373714 | 1.53E-50 | postive |
| PJVK | LINC01089 | 0.65167262 | 1.81E-66 | postive |
| GSDMB | AC009065.4 | 0.67688243 | 1.77E-73 | postive |
| PJVK | AC009065.4 | 0.65001634 | 4.96E-66 | postive |
| GSDMB | AP003352.1 | 0.70063714 | 9.31E-81 | postive |
| NLRP1 | AP003352.1 | 0.5139079 | 1.16E-37 | postive |
| PJVK | AP003352.1 | 0.61309847 | 5.96E-57 | postive |
| GSDMB | AL356481.3 | 0.66022369 | 9.04E-69 | postive |
| NLRP1 | AL356481.3 | 0.62455653 | 1.22E-59 | postive |
| PLCG1 | AL356481.3 | 0.55912891 | 1.19E-45 | postive |
| GSDMB | AC013468.1 | 0.56539011 | 7.39E-47 | postive |
| PJVK | AC013468.1 | 0.60159719 | 2.32E-54 | postive |
| SCAF11 | AC036214.2 | 0.50291382 | 6.79E-36 | postive |
| NLRP1 | AC135178.2 | 0.63846989 | 4.67E-63 | postive |
| NOD1 | AC135178.2 | 0.56764638 | 2.68E-47 | postive |
| PLCG1 | AC135178.2 | 0.56657721 | 4.34E-47 | postive |
| SCAF11 | AC006059.1 | 0.54947677 | 7.63E-44 | postive |
| SCAF11 | WARS2-AS1 | 0.50037359 | 1.70E-35 | postive |
| IL18 | IQCH-AS1 | 0.62816904 | 1.65E-60 | postive |
| GSDMB | ELF3-AS1 | 0.61593805 | 1.32E-57 | postive |
| GSDMB | AC126118.1 | 0.65156201 | 1.94E-66 | postive |
| PJVK | AC126118.1 | 0.60726595 | 1.26E-55 | postive |
| GSDMB | LMNTD2-AS1 | 0.60976891 | 3.44E-56 | postive |
| PJVK | LMNTD2-AS1 | 0.56922231 | 1.31E-47 | postive |
| GSDMB | AC012073.1 | 0.63951717 | 2.54E-63 | postive |
| GSDMB | AC022400.1 | 0.54134121 | 2.30E-42 | postive |
| SCAF11 | AL139407.1 | 0.57054342 | 7.20E-48 | postive |
| GSDMB | AC144548.1 | 0.58203596 | 3.43E-50 | postive |
| TIRAP | AL133355.1 | 0.56925719 | 1.29E-47 | postive |
| SCAF11 | AC006064.2 | 0.53037346 | 1.97E-40 | postive |
| GSDMB | OGFR-AS1 | 0.63845821 | 4.70E-63 | postive |
| IL18 | AC009779.2 | 0.56154795 | 4.09E-46 | postive |
| GSDMB | MRPS9-AS1 | 0.62079925 | 9.58E-59 | postive |
| NLRP1 | MRPS9-AS1 | 0.51940375 | 1.43E-38 | postive |
| PJVK | MRPS9-AS1 | 0.51853578 | 2.00E-38 | postive |
| GSDMB | AC138207.4 | 0.52328932 | 3.19E-39 | postive |
| NLRP1 | AC138207.4 | 0.51008601 | 4.85E-37 | postive |
| NOD1 | AC138207.4 | 0.50957036 | 5.88E-37 | postive |
| GSDMB | DPP9-AS1 | 0.75145707 | 4.77E-99 | postive |
| NLRP1 | DPP9-AS1 | 0.56924654 | 1.30E-47 | postive |
| PJVK | DPP9-AS1 | 0.54619711 | 3.05E-43 | postive |
| GSDMB | AL358472.2 | 0.53647285 | 1.69E-41 | postive |
| NLRP1 | AL358472.2 | 0.55606002 | 4.52E-45 | postive |
| PJVK | AL358472.2 | 0.56903106 | 1.43E-47 | postive |
| GSDMB | AC022400.4 | 0.61038161 | 2.49E-56 | postive |
| NLRP1 | AC022400.4 | 0.50453981 | 3.75E-36 | postive |
| GSDMB | TTC28-AS1 | 0.57488168 | 9.81E-49 | postive |
| NLRP1 | TTC28-AS1 | 0.52458729 | 1.93E-39 | postive |
| PJVK | TTC28-AS1 | 0.5222475 | 4.79E-39 | postive |
| GSDMB | AC087623.1 | 0.60113074 | 2.94E-54 | postive |
| NOD1 | LINC00989 | 0.50514804 | 3.00E-36 | postive |
| SCAF11 | PAXIP1-AS2 | 0.67539543 | 4.79E-73 | postive |
| TIRAP | PAXIP1-AS2 | 0.58172382 | 3.98E-50 | postive |
| GSDMB | SUGT1-DT | 0.6295935 | 7.41E-61 | postive |
| PJVK | SUGT1-DT | 0.6845779 | 9.24E-76 | postive |
| GSDMB | AC092375.2 | 0.5397538 | 4.43E-42 | postive |
| NLRP1 | AC092375.2 | 0.56274116 | 2.41E-46 | postive |
| SCAF11 | AL390195.2 | 0.54830424 | 1.25E-43 | postive |
| GSDMB | AL008729.1 | 0.59845106 | 1.14E-53 | postive |
| PJVK | AL008729.1 | 0.52049233 | 9.43E-39 | postive |
| GSDMB | AL513320.1 | 0.68039468 | 1.64E-74 | postive |
| NLRP1 | AL513320.1 | 0.53176563 | 1.13E-40 | postive |
| PJVK | AL513320.1 | 0.51961759 | 1.32E-38 | postive |
| SCAF11 | MALAT1 | 0.51237956 | 2.06E-37 | postive |
| SCAF11 | PABPC4-AS1 | 0.55998951 | 8.12E-46 | postive |
| SCAF11 | C1QTNF7-AS1 | 0.52178745 | 5.72E-39 | postive |
| SCAF11 | AC008937.3 | 0.55994841 | 8.27E-46 | postive |
| TIRAP | CENATAC-DT | 0.59255702 | 2.13E-52 | postive |
| SCAF11 | AC022558.1 | 0.50137461 | 1.19E-35 | postive |
| GSDMB | AL031705.1 | 0.69591198 | 2.97E-79 | postive |
| NLRP1 | AL031705.1 | 0.51276855 | 1.78E-37 | postive |
| PJVK | AL031705.1 | 0.53562893 | 2.38E-41 | postive |
| NLRC4 | AC073046.1 | 0.50652943 | 1.81E-36 | postive |
| NOD1 | AC073046.1 | 0.51357438 | 1.31E-37 | postive |
| SCAF11 | AC073046.1 | 0.58730694 | 2.75E-51 | postive |
| GSDMB | ZFHX2-AS1 | 0.60680316 | 1.61E-55 | postive |
| PJVK | ZFHX2-AS1 | 0.50021808 | 1.80E-35 | postive |
| GSDMB | LINC00894 | 0.57816474 | 2.13E-49 | postive |
| PJVK | LINC00894 | 0.53567689 | 2.34E-41 | postive |
| TIRAP | AC120114.1 | 0.63138708 | 2.70E-61 | postive |
| GSDMB | AC016026.1 | 0.53350986 | 5.61E-41 | postive |
| GSDMB | AC100803.4 | 0.576062 | 5.67E-49 | postive |
| NLRP1 | AC100803.4 | 0.61771677 | 5.07E-58 | postive |
| CASP4 | LINC01871 | 0.50348401 | 5.51E-36 | postive |
| GSDMB | LINC01004 | 0.60273588 | 1.30E-54 | postive |
| PJVK | LINC01004 | 0.59136831 | 3.82E-52 | postive |
| GPX4 | AC138696.2 | 0.53539465 | 2.62E-41 | postive |
| GSDMB | AL450384.2 | 0.62125978 | 7.45E-59 | postive |
| PJVK | AL450384.2 | 0.57908356 | 1.38E-49 | postive |
| GSDMB | C1RL-AS1 | 0.50666902 | 1.72E-36 | postive |
| GSDMB | AP001767.2 | 0.56079176 | 5.71E-46 | postive |
| GSDMB | AC015802.4 | 0.74065585 | 8.39E-95 | postive |
| NLRP1 | AC015802.4 | 0.50687755 | 1.59E-36 | postive |
| PJVK | AC015802.4 | 0.69306118 | 2.33E-78 | postive |
| GPX4 | AC005288.1 | -0.5736412 | 1.74E-48 | negative |
| SCAF11 | AC005288.1 | 0.60189438 | 1.99E-54 | postive |
| TIRAP | AC005288.1 | 0.52315724 | 3.36E-39 | postive |
| GSDMB | AC105020.6 | 0.5452725 | 4.49E-43 | postive |
| NLRP1 | AC105020.6 | 0.5500638 | 5.95E-44 | postive |
| GSDMB | AC107375.1 | 0.64559265 | 7.07E-65 | postive |
| NLRP1 | AC107375.1 | 0.62393523 | 1.72E-59 | postive |
| PJVK | AC107375.1 | 0.53651287 | 1.66E-41 | postive |
| PLCG1 | AC107375.1 | 0.59414787 | 9.71E-53 | postive |
| GSDMB | AC003101.2 | 0.50594806 | 2.24E-36 | postive |
| PJVK | AC003101.2 | 0.52046818 | 9.52E-39 | postive |
| CASP1 | AL365361.1 | 0.51209998 | 2.29E-37 | postive |
| GSDMB | ALOX12-AS1 | 0.52778355 | 5.49E-40 | postive |
| GSDMB | LINC02352 | 0.63975061 | 2.22E-63 | postive |
| PJVK | LINC02352 | 0.61795574 | 4.46E-58 | postive |
| GSDMB | SLC16A1-AS1 | 0.61807468 | 4.19E-58 | postive |
| SCAF11 | AC087071.2 | 0.56148243 | 4.21E-46 | postive |
| PJVK | AL121583.1 | 0.54544909 | 4.17E-43 | postive |
| GSDMB | AP001107.4 | 0.61411065 | 3.49E-57 | postive |
| NLRP1 | AP001107.4 | 0.55064637 | 4.64E-44 | postive |
| PJVK | AP001107.4 | 0.51597995 | 5.30E-38 | postive |
| SCAF11 | AL121672.2 | 0.53481789 | 3.31E-41 | postive |
| GSDMB | LINC00685 | 0.60400536 | 6.79E-55 | postive |
| PJVK | LINC00685 | 0.54124145 | 2.40E-42 | postive |
| GSDMB | INTS6-AS1 | 0.60709838 | 1.38E-55 | postive |
| PJVK | INTS6-AS1 | 0.53924465 | 5.46E-42 | postive |
| GSDMB | KDM4A-AS1 | 0.69143948 | 7.43E-78 | postive |
| NLRP1 | KDM4A-AS1 | 0.53055081 | 1.83E-40 | postive |
| PJVK | KDM4A-AS1 | 0.5631542 | 2.00E-46 | postive |
| GSDMB | AC022167.2 | 0.71757319 | 2.10E-86 | postive |
| NLRP1 | AC022167.2 | 0.55200468 | 2.60E-44 | postive |
| PJVK | AC022167.2 | 0.53031039 | 2.02E-40 | postive |
| TIRAP | AC008764.2 | 0.53125131 | 1.39E-40 | postive |
| GSDMB | KMT2E-AS1 | 0.57633436 | 5.00E-49 | postive |
| PJVK | KMT2E-AS1 | 0.58946841 | 9.65E-52 | postive |
| GSDMB | AC010542.5 | 0.69943173 | 2.27E-80 | postive |
| NLRP1 | AC010542.5 | 0.52291788 | 3.69E-39 | postive |
| PJVK | AC010542.5 | 0.69733075 | 1.06E-79 | postive |
| SCAF11 | AC005034.5 | 0.73956041 | 2.20E-94 | postive |
| IL18 | NUP50-DT | 0.50685075 | 1.61E-36 | postive |
| GSDMB | ZNF529-AS1 | 0.57346488 | 1.89E-48 | postive |
| PJVK | ZNF529-AS1 | 0.52092516 | 7.98E-39 | postive |
| GSDMB | AC026803.1 | 0.54404923 | 7.49E-43 | postive |
| NLRP1 | AP001189.1 | 0.51389172 | 1.17E-37 | postive |
| NOD1 | AP001189.1 | 0.5336055 | 5.39E-41 | postive |
| SCAF11 | AL049840.6 | 0.56444556 | 1.13E-46 | postive |
| SCAF11 | AC098484.1 | 0.6335461 | 7.92E-62 | postive |
| TIRAP | AC098484.1 | 0.59034556 | 6.29E-52 | postive |
| GSDMB | AL117379.1 | 0.7051837 | 3.11E-82 | postive |
| NLRP1 | AL117379.1 | 0.50475996 | 3.46E-36 | postive |
| PJVK | AL117379.1 | 0.64624042 | 4.81E-65 | postive |
| PJVK | NBR2 | 0.53469042 | 3.48E-41 | postive |
| GSDMB | AC254562.3 | 0.53611812 | 1.95E-41 | postive |
| GSDMB | AP001160.1 | 0.81444385 | 4.83E-129 | postive |
| NLRP1 | AP001160.1 | 0.56916871 | 1.34E-47 | postive |
| PJVK | AP001160.1 | 0.64341876 | 2.57E-64 | postive |
| GSDMB | AL354733.3 | 0.58166404 | 4.09E-50 | postive |
| NLRP1 | AL354733.3 | 0.54960189 | 7.24E-44 | postive |
| IL18 | AC135507.1 | 0.54221399 | 1.60E-42 | postive |
| GSDMB | AL136304.1 | 0.61399355 | 3.71E-57 | postive |
| NLRP1 | AL136304.1 | 0.50911461 | 6.96E-37 | postive |
| GSDMB | AC120053.1 | 0.6165388 | 9.55E-58 | postive |
| PJVK | AC120053.1 | 0.5711551 | 5.44E-48 | postive |
| GSDMB | AC010463.3 | 0.57684194 | 3.95E-49 | postive |
| GSDMB | AL442128.2 | 0.62828528 | 1.54E-60 | postive |
| PJVK | AL442128.2 | 0.64162634 | 7.39E-64 | postive |
| GSDMB | AP000553.2 | 0.60672642 | 1.67E-55 | postive |
| PJVK | AP000553.2 | 0.61488723 | 2.31E-57 | postive |
| AIM2 | BHLHE40-AS1 | 0.51577427 | 5.73E-38 | postive |
| CASP1 | BHLHE40-AS1 | 0.59389367 | 1.10E-52 | postive |
| NOD2 | BHLHE40-AS1 | 0.67406175 | 1.17E-72 | postive |
| GSDMB | AC002398.1 | 0.58307874 | 2.09E-50 | postive |
| SCAF11 | AC021483.2 | 0.54296229 | 1.18E-42 | postive |
| NLRP3 | AL161785.1 | 0.5466272 | 2.54E-43 | postive |
| GSDMB | AL356299.2 | 0.58714831 | 2.97E-51 | postive |
| PJVK | AL356299.2 | 0.50000811 | 1.94E-35 | postive |
| AIM2 | PDE2A-AS2 | 0.50260815 | 7.58E-36 | postive |
| GSDMB | AC010331.1 | 0.58455539 | 1.03E-50 | postive |
| PJVK | AC010331.1 | 0.61275809 | 7.14E-57 | postive |
| GSDMB | AL133410.1 | 0.74439465 | 3.01E-96 | postive |
| PJVK | AL133410.1 | 0.66384854 | 9.06E-70 | postive |
| GSDMB | AC084876.1 | 0.64635383 | 4.49E-65 | postive |
| NLRP1 | AC084876.1 | 0.50518165 | 2.97E-36 | postive |
| PJVK | AC084876.1 | 0.52921229 | 3.12E-40 | postive |
| GSDMB | AL683813.1 | 0.6430743 | 3.15E-64 | postive |
| PJVK | AL683813.1 | 0.52079877 | 8.38E-39 | postive |
| SCAF11 | AC005070.3 | 0.54292643 | 1.19E-42 | postive |
| AIM2 | AC004585.1 | 0.53945601 | 5.00E-42 | postive |
| CASP1 | AC004585.1 | 0.62728846 | 2.69E-60 | postive |
| CASP4 | AC004585.1 | 0.59963371 | 6.26E-54 | postive |
| NOD2 | AC004585.1 | 0.71496955 | 1.65E-85 | postive |
| GSDMB | AC016737.1 | 0.7377504 | 1.07E-93 | postive |
| NLRP1 | AC016737.1 | 0.50954832 | 5.93E-37 | postive |
| PJVK | AC016737.1 | 0.71246576 | 1.17E-84 | postive |
| SCAF11 | AC074138.1 | 0.51803885 | 2.42E-38 | postive |
| GSDMB | AC055855.1 | 0.71303161 | 7.54E-85 | postive |
| NLRP1 | AC055855.1 | 0.60362174 | 8.26E-55 | postive |
| PJVK | AC055855.1 | 0.58955225 | 9.26E-52 | postive |
| PLCG1 | AC055855.1 | 0.51045168 | 4.23E-37 | postive |
| GSDMB | AL158196.1 | 0.56682573 | 3.88E-47 | postive |
| PJVK | AL158196.1 | 0.52960589 | 2.67E-40 | postive |
| NLRP1 | GRPEL2-AS1 | 0.61448082 | 2.86E-57 | postive |
| NOD1 | GRPEL2-AS1 | 0.61446562 | 2.89E-57 | postive |
| PLCG1 | GRPEL2-AS1 | 0.57782559 | 2.49E-49 | postive |
| GSDMB | AC024060.2 | 0.70915617 | 1.51E-83 | postive |
| NLRP1 | AC024060.2 | 0.56383484 | 1.48E-46 | postive |
| PJVK | AC024060.2 | 0.66595894 | 2.34E-70 | postive |
| GSDMB | AC132872.3 | 0.541687 | 2.00E-42 | postive |
| IL6 | AC132872.3 | 0.55648405 | 3.76E-45 | postive |
| SCAF11 | AC084871.1 | 0.51094507 | 3.52E-37 | postive |
| TIRAP | AC005332.6 | 0.53488382 | 3.22E-41 | postive |
| GSDMB | SNHG10 | 0.57862177 | 1.72E-49 | postive |
| PJVK | SNHG10 | 0.58958743 | 9.11E-52 | postive |
| SCAF11 | AL353804.2 | 0.51216405 | 2.23E-37 | postive |
| GSDMB | AL021707.8 | 0.71271299 | 9.67E-85 | postive |
| NLRP1 | AL021707.8 | 0.57428258 | 1.29E-48 | postive |
| GSDMB | AL096701.3 | 0.6940445 | 1.15E-78 | postive |
| NLRP1 | AL096701.3 | 0.52004187 | 1.12E-38 | postive |
| PJVK | AL096701.3 | 0.60065573 | 3.74E-54 | postive |
| GSDMB | AC011498.6 | 0.59181575 | 3.06E-52 | postive |
| SCAF11 | AC011477.2 | 0.60249175 | 1.47E-54 | postive |
| GSDMB | U47924.3 | 0.70625314 | 1.39E-82 | postive |
| PJVK | U47924.3 | 0.5958349 | 4.20E-53 | postive |
| SCAF11 | AC008124.1 | 0.52262223 | 4.14E-39 | postive |
| GSDMB | AL358472.3 | 0.54793654 | 1.47E-43 | postive |
| PJVK | AL358472.3 | 0.61994691 | 1.52E-58 | postive |
| GSDMB | AL031600.1 | 0.63197948 | 1.93E-61 | postive |
| NLRP1 | AL031600.1 | 0.52745409 | 6.25E-40 | postive |
| AIM2 | AC022126.1 | 0.51337732 | 1.42E-37 | postive |
| GSDMB | Z98884.2 | 0.56625164 | 5.02E-47 | postive |
| NLRP1 | Z98884.2 | 0.51769646 | 2.76E-38 | postive |
| SCAF11 | AC010261.1 | 0.51560324 | 6.11E-38 | postive |
| GSDMB | AC011468.1 | 0.64684793 | 3.34E-65 | postive |
| PJVK | AC011468.1 | 0.53562295 | 2.39E-41 | postive |
| GSDMB | AC138932.4 | 0.56101812 | 5.16E-46 | postive |
| GSDMB | MORF4L2-AS1 | 0.50630609 | 1.96E-36 | postive |
| PJVK | SUV39H2-DT | 0.50410697 | 4.39E-36 | postive |
| GSDMB | SBNO1-AS1 | 0.69248853 | 3.51E-78 | postive |
| PJVK | SBNO1-AS1 | 0.6650084 | 4.31E-70 | postive |
| GSDMB | AC020915.1 | 0.57287156 | 2.48E-48 | postive |
| SCAF11 | AC096586.2 | 0.52688234 | 7.83E-40 | postive |
| SCAF11 | AC138150.1 | 0.51338686 | 1.41E-37 | postive |
| SCAF11 | TNFRSF10A-AS1 | 0.54055098 | 3.19E-42 | postive |
| SCAF11 | OPA1-AS1 | 0.51582233 | 5.62E-38 | postive |
| PJVK | AC012313.6 | 0.50116424 | 1.28E-35 | postive |
| GSDMB | AL021707.2 | 0.56586864 | 5.96E-47 | postive |
| PJVK | AL021707.2 | 0.55188477 | 2.74E-44 | postive |
| IL18 | LINC00265 | 0.56927241 | 1.28E-47 | postive |
| AIM2 | AC091057.1 | 0.52523122 | 1.50E-39 | postive |
| GSDMB | AC091057.1 | 0.65503143 | 2.31E-67 | postive |
| GSDMB | LAMC1-AS1 | 0.50957352 | 5.87E-37 | postive |
| NLRP1 | LAMC1-AS1 | 0.50069421 | 1.52E-35 | postive |
| NOD1 | LAMC1-AS1 | 0.53319673 | 6.36E-41 | postive |
| GSDMB | AL731563.3 | 0.52109657 | 7.47E-39 | postive |
| SCAF11 | ALG13-AS1 | 0.51218656 | 2.21E-37 | postive |
| SCAF11 | ZKSCAN7-AS1 | 0.50036769 | 1.71E-35 | postive |
| GSDMB | ASMTL-AS1 | 0.66897178 | 3.32E-71 | postive |
| PJVK | ASMTL-AS1 | 0.53538429 | 2.63E-41 | postive |
| GSDMB | RAD51-AS1 | 0.66730447 | 9.82E-71 | postive |
| NLRP1 | RAD51-AS1 | 0.57021299 | 8.36E-48 | postive |
| PJVK | RAD51-AS1 | 0.60307204 | 1.09E-54 | postive |
| GSDMB | CAPN10-DT | 0.70852848 | 2.45E-83 | postive |
| PJVK | CAPN10-DT | 0.60718568 | 1.32E-55 | postive |
| GSDMB | AC025171.5 | 0.53438103 | 3.95E-41 | postive |
| NLRP1 | AC025171.5 | 0.50705612 | 1.49E-36 | postive |
| GSDMB | AC034236.2 | 0.65399006 | 4.38E-67 | postive |
| NLRP1 | AC034236.2 | 0.50193038 | 9.70E-36 | postive |
| PJVK | AC034236.2 | 0.61459882 | 2.69E-57 | postive |
| SCAF11 | AC092755.1 | 0.51068577 | 3.88E-37 | postive |
| GSDMB | AC009318.1 | 0.50359221 | 5.30E-36 | postive |
| GSDMB | DBH-AS1 | 0.63960474 | 2.41E-63 | postive |
| SCAF11 | KLF7-IT1 | 0.52358922 | 2.84E-39 | postive |
| GSDMB | AL161452.1 | 0.67559167 | 4.20E-73 | postive |
| NLRP1 | AL161452.1 | 0.57209764 | 3.54E-48 | postive |
| IL18 | LINC01187 | 0.59178788 | 3.11E-52 | postive |
| GSDMB | AC244197.2 | 0.63565702 | 2.37E-62 | postive |
| GSDMB | AC018690.1 | 0.62007759 | 1.42E-58 | postive |
| PJVK | AC018690.1 | 0.5749226 | 9.62E-49 | postive |
| AIM2 | LINC00941 | 0.5884896 | 1.55E-51 | postive |
| GSDMB | AL162586.1 | 0.70760078 | 4.97E-83 | postive |
| NLRP1 | AL162586.1 | 0.69005051 | 1.99E-77 | postive |
| NOD1 | AL162586.1 | 0.54321455 | 1.06E-42 | postive |
| PJVK | AL162586.1 | 0.50482148 | 3.38E-36 | postive |
| PLCG1 | AL162586.1 | 0.60657188 | 1.81E-55 | postive |
| GSDMB | ARHGAP27P1-BPTFP1-KPNA2P3 | 0.78688093 | 1.16E-114 | postive |
| NLRP1 | ARHGAP27P1-BPTFP1-KPNA2P3 | 0.8166478 | 2.70E-130 | postive |
| NOD1 | ARHGAP27P1-BPTFP1-KPNA2P3 | 0.576198 | 5.33E-49 | postive |
| PJVK | ARHGAP27P1-BPTFP1-KPNA2P3 | 0.61253936 | 8.02E-57 | postive |
| PLCG1 | ARHGAP27P1-BPTFP1-KPNA2P3 | 0.65428956 | 3.64E-67 | postive |
| GSDMB | RASGRP3-AS1 | 0.6641046 | 7.70E-70 | postive |
| PJVK | RASGRP3-AS1 | 0.60424078 | 6.02E-55 | postive |
| GSDMB | YEATS2-AS1 | 0.7271852 | 8.52E-90 | postive |
| NLRP1 | YEATS2-AS1 | 0.63086482 | 3.62E-61 | postive |
| NOD1 | YEATS2-AS1 | 0.51034221 | 4.41E-37 | postive |
| PLCG1 | YEATS2-AS1 | 0.54865789 | 1.08E-43 | postive |
| GSDMB | SNHG21 | 0.63721253 | 9.66E-63 | postive |
| NLRP1 | SNHG21 | 0.52717173 | 6.99E-40 | postive |
| PJVK | SNHG21 | 0.52494307 | 1.68E-39 | postive |
| GSDMB | RUSC1-AS1 | 0.77206672 | 8.65E-108 | postive |
| NLRP1 | RUSC1-AS1 | 0.64076527 | 1.22E-63 | postive |
| PJVK | RUSC1-AS1 | 0.60067063 | 3.71E-54 | postive |
| PLCG1 | RUSC1-AS1 | 0.52465864 | 1.87E-39 | postive |
| GSDMB | OSGEPL1-AS1 | 0.58921312 | 1.09E-51 | postive |
| PJVK | OSGEPL1-AS1 | 0.57731208 | 3.17E-49 | postive |
| GSDMB | AC005332.5 | 0.67318044 | 2.09E-72 | postive |
| NLRP1 | AC005332.5 | 0.50787862 | 1.10E-36 | postive |
| PJVK | AC005332.5 | 0.55458182 | 8.58E-45 | postive |
| GSDMB | AL109659.2 | 0.60317808 | 1.04E-54 | postive |
| NLRP1 | AL109659.2 | 0.61201518 | 1.06E-56 | postive |
| NOD1 | AL109659.2 | 0.52906912 | 3.30E-40 | postive |
| PLCG1 | AL109659.2 | 0.53558719 | 2.42E-41 | postive |
| GSDMB | LINC01311 | 0.69952904 | 2.11E-80 | postive |
| NLRP1 | LINC01311 | 0.5163734 | 4.56E-38 | postive |
| PJVK | LINC01311 | 0.62733975 | 2.62E-60 | postive |
| CASP9 | AC007785.1 | 0.50106256 | 1.33E-35 | postive |
| SCAF11 | AF129075.1 | 0.50599193 | 2.20E-36 | postive |
| GSDMB | AC019186.1 | 0.51199713 | 2.38E-37 | postive |
| PJVK | AC019186.1 | 0.55897458 | 1.27E-45 | postive |
| GSDMB | AC002128.1 | 0.5868205 | 3.48E-51 | postive |
| PJVK | AC002128.1 | 0.53436455 | 3.97E-41 | postive |
| CASP4 | LINC01943 | 0.51280552 | 1.76E-37 | postive |
| GSDMB | WAKMAR2 | 0.56723207 | 3.23E-47 | postive |
| NLRP1 | WAKMAR2 | 0.57438298 | 1.23E-48 | postive |
| SCAF11 | Z68871.1 | 0.70550585 | 2.44E-82 | postive |
| GSDMB | Z97832.2 | 0.66201713 | 2.91E-69 | postive |
| NLRP1 | Z97832.2 | 0.53473577 | 3.42E-41 | postive |
| PJVK | Z97832.2 | 0.55334669 | 1.46E-44 | postive |
| GSDMB | AL133406.2 | 0.60110577 | 2.97E-54 | postive |
| NLRP1 | AL133406.2 | 0.59555879 | 4.82E-53 | postive |
| NOD1 | AL133406.2 | 0.52312162 | 3.41E-39 | postive |
| PLCG1 | AL133406.2 | 0.54124433 | 2.40E-42 | postive |
| SCAF11 | LINC01515 | 0.54272377 | 1.30E-42 | postive |
| GSDMB | AL355385.1 | 0.5462657 | 2.96E-43 | postive |
| PJVK | AL355385.1 | 0.5019461 | 9.64E-36 | postive |
| SCAF11 | DLEU2 | 0.5400199 | 3.97E-42 | postive |
| GSDMB | AC007292.1 | 0.54520772 | 4.61E-43 | postive |
| GSDMB | TMEM147-AS1 | 0.73887651 | 4.01E-94 | postive |
| NLRP1 | TMEM147-AS1 | 0.51474844 | 8.44E-38 | postive |
| PJVK | TMEM147-AS1 | 0.68067542 | 1.35E-74 | postive |
| PLCG1 | TMEM147-AS1 | 0.5209731 | 7.84E-39 | postive |
| GSDMB | AC087741.1 | 0.7708867 | 2.90E-107 | postive |
| NLRP1 | AC087741.1 | 0.59822243 | 1.27E-53 | postive |
| PJVK | AC087741.1 | 0.60429997 | 5.84E-55 | postive |
| SCAF11 | AC234775.2 | 0.74818321 | 9.73E-98 | postive |
| GSDMB | AC026356.2 | 0.56048447 | 6.53E-46 | postive |
| GSDMB | AL645940.1 | 0.69786319 | 7.17E-80 | postive |
| NLRP1 | AL645940.1 | 0.54540249 | 4.25E-43 | postive |
| PJVK | AL645940.1 | 0.59970388 | 6.05E-54 | postive |
| GSDMB | AC112722.1 | 0.52528248 | 1.47E-39 | postive |
| PJVK | AC112722.1 | 0.50838994 | 9.11E-37 | postive |
| GSDMB | AC063948.1 | 0.65136323 | 2.19E-66 | postive |
| PJVK | AC063948.1 | 0.58949017 | 9.55E-52 | postive |
| SCAF11 | LINC00216 | 0.54386107 | 8.10E-43 | postive |
| GSDMB | AC093249.6 | 0.54422584 | 6.95E-43 | postive |
| PJVK | AC093249.6 | 0.67210366 | 4.26E-72 | postive |
| GSDMB | MED8-AS1 | 0.69793152 | 6.82E-80 | postive |
| NLRP1 | MED8-AS1 | 0.57952226 | 1.13E-49 | postive |
| SCAF11 | GMDS-DT | 0.54421779 | 6.98E-43 | postive |
| GSDMB | AL592211.1 | 0.71680043 | 3.89E-86 | postive |
| NLRP1 | AL592211.1 | 0.53091271 | 1.59E-40 | postive |
| PJVK | AL592211.1 | 0.5348492 | 3.27E-41 | postive |
| GSDMB | AL683807.1 | 0.65339988 | 6.29E-67 | postive |
| NLRP1 | AL683807.1 | 0.53263328 | 7.97E-41 | postive |
| SCAF11 | AL161782.1 | 0.5172165 | 3.31E-38 | postive |
| SCAF11 | AL158163.2 | 0.51998141 | 1.15E-38 | postive |
| GSDMB | AC105020.1 | 0.58294368 | 2.23E-50 | postive |
| NLRP1 | AC105020.1 | 0.5187485 | 1.84E-38 | postive |
| GSDMB | KIF1C-AS1 | 0.795191 | 9.16E-119 | postive |
| NLRP1 | KIF1C-AS1 | 0.64162408 | 7.40E-64 | postive |
| GSDMB | AC084824.5 | 0.65776262 | 4.23E-68 | postive |
| NLRP1 | AC084824.5 | 0.52080223 | 8.37E-39 | postive |
| PJVK | AC084824.5 | 0.61004094 | 2.98E-56 | postive |
| PJVK | AC104794.3 | 0.68164352 | 6.98E-75 | postive |
| SCAF11 | AC007000.3 | 0.54925605 | 8.38E-44 | postive |
| GSDMB | AC010201.1 | 0.5840571 | 1.31E-50 | postive |
| PJVK | AC010201.1 | 0.6061724 | 2.23E-55 | postive |
| GSDMB | AC092301.1 | 0.63616988 | 1.76E-62 | postive |
| NLRP1 | AC092301.1 | 0.54621074 | 3.03E-43 | postive |
| NOD1 | TRAF3IP2-AS1 | 0.50716006 | 1.43E-36 | postive |
| GSDMB | AC073655.2 | 0.66701936 | 1.18E-70 | postive |
| PJVK | AC073655.2 | 0.58782765 | 2.14E-51 | postive |
| GSDMB | LINC00173 | 0.51568877 | 5.91E-38 | postive |
| GSDMB | AL157871.2 | 0.51529611 | 6.86E-38 | postive |
| IL18 | AL031710.1 | 0.53319533 | 6.36E-41 | postive |
| IL18 | SBF2-AS1 | 0.50825773 | 9.56E-37 | postive |
| SCAF11 | AL049869.2 | 0.55831233 | 1.70E-45 | postive |
| PJVK | LINC01431 | 0.5839648 | 1.37E-50 | postive |
| GSDMB | NRIR | 0.63897931 | 3.47E-63 | postive |
| NLRP1 | NRIR | 0.5380889 | 8.76E-42 | postive |
| GSDMB | AC012645.3 | 0.66784089 | 6.93E-71 | postive |
| NLRP1 | AC012645.3 | 0.61053584 | 2.30E-56 | postive |
| NOD2 | AC012645.3 | 0.58568494 | 6.01E-51 | postive |
| GSDMB | AL731569.1 | 0.63274425 | 1.25E-61 | postive |
| PJVK | AL731569.1 | 0.62520676 | 8.54E-60 | postive |
| SCAF11 | SLC16A12-AS1 | 0.50072901 | 1.50E-35 | postive |
| PJVK | AC116407.2 | 0.5061148 | 2.11E-36 | postive |
| GSDMB | AP002490.1 | 0.62596535 | 5.61E-60 | postive |
| NLRP1 | AP002490.1 | 0.51350139 | 1.35E-37 | postive |
| PJVK | AP002490.1 | 0.51343197 | 1.39E-37 | postive |
| GSDMB | AC009404.1 | 0.65783945 | 4.03E-68 | postive |
| PJVK | AC009404.1 | 0.60709328 | 1.38E-55 | postive |
| GSDMB | AC005104.1 | 0.69783313 | 7.33E-80 | postive |
| NLRP1 | AC005104.1 | 0.63634931 | 1.59E-62 | postive |
| NOD1 | AC005104.1 | 0.52645457 | 9.27E-40 | postive |
| PLCG1 | AC005104.1 | 0.52235494 | 4.59E-39 | postive |
| GSDMB | SNHG12 | 0.51351881 | 1.34E-37 | postive |
| SCAF11 | AC093157.2 | 0.50281555 | 7.03E-36 | postive |
| GSDMB | AC011481.1 | 0.59615076 | 3.59E-53 | postive |
| PJVK | AC011481.1 | 0.53570883 | 2.31E-41 | postive |
| GSDMB | NALT1 | 0.68722749 | 1.46E-76 | postive |
| PJVK | NALT1 | 0.68015014 | 1.94E-74 | postive |
| SCAF11 | FBXO30-DT | 0.6096238 | 3.71E-56 | postive |
| GSDMB | SLBP-DT | 0.79003477 | 3.38E-116 | postive |
| NLRP1 | SLBP-DT | 0.54142666 | 2.22E-42 | postive |
| PJVK | SLBP-DT | 0.63350425 | 8.11E-62 | postive |
| PJVK | AL035461.2 | 0.51709681 | 3.46E-38 | postive |
| SCAF11 | AC008770.3 | 0.60208014 | 1.81E-54 | postive |
| GSDMB | GHRLOS | 0.59247201 | 2.22E-52 | postive |
| NLRP1 | GHRLOS | 0.50750605 | 1.26E-36 | postive |
| NLRP1 | AC010175.1 | 0.55705214 | 2.94E-45 | postive |
| NLRP1 | AC010976.2 | 0.59114145 | 4.26E-52 | postive |
| PLCG1 | AC010976.2 | 0.52760374 | 5.90E-40 | postive |
| GSDMB | LINC01341 | 0.55740326 | 2.52E-45 | postive |
| PJVK | LINC01341 | 0.51536956 | 6.67E-38 | postive |
| AIM2 | TRG-AS1 | 0.50493927 | 3.24E-36 | postive |
| CASP1 | TRG-AS1 | 0.66068398 | 6.76E-69 | postive |
| CASP4 | TRG-AS1 | 0.56432976 | 1.19E-46 | postive |
| GSDMB | TRG-AS1 | 0.52416327 | 2.27E-39 | postive |
| NOD2 | TRG-AS1 | 0.70942058 | 1.23E-83 | postive |
| TIRAP | AL122035.1 | 0.52491841 | 1.69E-39 | postive |
| GSDMB | AC079807.1 | 0.55855533 | 1.52E-45 | postive |
| NLRP1 | AC079807.1 | 0.52753073 | 6.07E-40 | postive |
| PJVK | AC079807.1 | 0.58634854 | 4.37E-51 | postive |
| GSDMB | AC079922.2 | 0.55995293 | 8.26E-46 | postive |
| PJVK | AC079922.2 | 0.55203215 | 2.57E-44 | postive |
| CASP1 | AC002091.1 | 0.57050022 | 7.34E-48 | postive |
| NLRC4 | AC002091.1 | 0.57828363 | 2.01E-49 | postive |
| NLRP3 | AC002091.1 | 0.56231994 | 2.90E-46 | postive |
| GSDMB | AP006621.3 | 0.61062345 | 2.20E-56 | postive |
| PJVK | AP006621.3 | 0.50393104 | 4.69E-36 | postive |
| GSDMB | FBXL19-AS1 | 0.50424987 | 4.17E-36 | postive |
| GSDMB | AC233728.1 | 0.64969386 | 6.03E-66 | postive |
| NLRP1 | AC233728.1 | 0.53286084 | 7.28E-41 | postive |
| CASP1 | AC090559.1 | 0.5320035 | 1.03E-40 | postive |
| NLRC4 | AC090559.1 | 0.5836521 | 1.59E-50 | postive |
| NLRP3 | AC090559.1 | 0.66570368 | 2.76E-70 | postive |
| NOD2 | AC090559.1 | 0.57798411 | 2.32E-49 | postive |
| GSDMB | ZNF528-AS1 | 0.62324304 | 2.52E-59 | postive |
| NLRP1 | ZNF528-AS1 | 0.55982714 | 8.73E-46 | postive |
| SCAF11 | AC108449.2 | 0.68733061 | 1.36E-76 | postive |
| TIRAP | AC108449.2 | 0.52463688 | 1.89E-39 | postive |
| GSDMB | BACE1-AS | 0.59967605 | 6.13E-54 | postive |
| PJVK | BACE1-AS | 0.58292884 | 2.25E-50 | postive |
| CASP1 | AC006033.2 | 0.62077054 | 9.73E-59 | postive |
| NLRC4 | AC006033.2 | 0.61145689 | 1.42E-56 | postive |
| NOD2 | AC006033.2 | 0.56408055 | 1.33E-46 | postive |
| NOD1 | ZBTB40-IT1 | 0.51022123 | 4.61E-37 | postive |
| GSDMB | AL590560.3 | 0.56481761 | 9.55E-47 | postive |
| PJVK | AL590560.3 | 0.53027985 | 2.04E-40 | postive |
| GSDMB | AC145423.2 | 0.58664451 | 3.79E-51 | postive |
| PJVK | AC145423.2 | 0.53107275 | 1.49E-40 | postive |
| IL6 | AC002480.1 | 0.70249116 | 2.35E-81 | postive |
| GSDMB | STAG3L5P-PVRIG2P-PILRB | 0.68398865 | 1.39E-75 | postive |
| NLRP1 | STAG3L5P-PVRIG2P-PILRB | 0.55058946 | 4.76E-44 | postive |
| PJVK | STAG3L5P-PVRIG2P-PILRB | 0.57925561 | 1.28E-49 | postive |
| GSDMB | LINC01011 | 0.50274651 | 7.21E-36 | postive |
| SCAF11 | FTX | 0.54307464 | 1.12E-42 | postive |
| GSDMB | AC022973.5 | 0.50676569 | 1.66E-36 | postive |
| GSDMB | AL021392.1 | 0.50714238 | 1.44E-36 | postive |
| GSDMB | AP005329.1 | 0.6177359 | 5.02E-58 | postive |
| PJVK | AP005329.1 | 0.61057691 | 2.25E-56 | postive |
| SCAF11 | AC010168.1 | 0.52415007 | 2.28E-39 | postive |
| NLRC4 | MACORIS | 0.52278313 | 3.89E-39 | postive |
| TIRAP | LINC01801 | 0.55132158 | 3.48E-44 | postive |
| GSDMB | LINC01160 | 0.75687463 | 2.92E-101 | postive |
| NLRP1 | LINC01160 | 0.66328449 | 1.30E-69 | postive |
| PJVK | LINC01160 | 0.56124797 | 4.67E-46 | postive |
| GSDMB | AC073195.1 | 0.58232302 | 3.00E-50 | postive |
| PJVK | AC073195.1 | 0.59725791 | 2.07E-53 | postive |
| NOD1 | STARD4-AS1 | 0.51952766 | 1.37E-38 | postive |
| GSDMB | AC004148.1 | 0.75873953 | 4.90E-102 | postive |
| NLRP1 | AC004148.1 | 0.64957385 | 6.48E-66 | postive |
| PJVK | AC004148.1 | 0.6525689 | 1.05E-66 | postive |
| PLCG1 | AC004148.1 | 0.55705276 | 2.94E-45 | postive |
| GSDMB | AC009118.3 | 0.65001738 | 4.95E-66 | postive |
| NLRP1 | AC009118.3 | 0.55622837 | 4.20E-45 | postive |
| PJVK | AC009118.3 | 0.6670253 | 1.18E-70 | postive |
| GSDMB | AP000873.3 | 0.52621821 | 1.02E-39 | postive |
| SCAF11 | AC018926.3 | 0.53769897 | 1.03E-41 | postive |
| GSDMB | LINC00115 | 0.7405504 | 9.21E-95 | postive |
| NLRP1 | LINC00115 | 0.63810505 | 5.77E-63 | postive |
| PJVK | LINC00115 | 0.59116532 | 4.21E-52 | postive |
| PLCG1 | LINC00115 | 0.54022952 | 3.64E-42 | postive |
| GSDMB | TMED2-DT | 0.71783918 | 1.70E-86 | postive |
| PJVK | TMED2-DT | 0.53803709 | 8.94E-42 | postive |
| SCAF11 | AC005034.3 | 0.56800171 | 2.28E-47 | postive |
| CASP1 | AC006369.1 | 0.5087998 | 7.82E-37 | postive |
| GSDMB | FMR1-IT1 | 0.50255399 | 7.73E-36 | postive |
| GSDMB | AL008582.1 | 0.70567493 | 2.15E-82 | postive |
| PJVK | AL008582.1 | 0.59546237 | 5.06E-53 | postive |
| SCAF11 | AP001429.1 | 0.52392127 | 2.50E-39 | postive |
| GSDMB | AC018809.1 | 0.65867274 | 2.39E-68 | postive |
| PJVK | AC018809.1 | 0.63989375 | 2.04E-63 | postive |
| NLRC4 | AC008957.1 | 0.55679102 | 3.29E-45 | postive |
| GSDMB | AC022098.1 | 0.55313778 | 1.60E-44 | postive |
| NLRP1 | AC022098.1 | 0.67725498 | 1.38E-73 | postive |
| NOD1 | AC022098.1 | 0.62044291 | 1.16E-58 | postive |
| PLCG1 | AC022098.1 | 0.61567599 | 1.52E-57 | postive |
| SCAF11 | AC087286.2 | 0.57848129 | 1.83E-49 | postive |
| GSDMB | AP001029.1 | 0.6681413 | 5.71E-71 | postive |
| PJVK | AP001029.1 | 0.56440922 | 1.15E-46 | postive |
| GSDMB | AL121890.5 | 0.53691344 | 1.41E-41 | postive |
| GPX4 | TP53TG1 | 0.58092277 | 5.81E-50 | postive |
| GSDMB | AC245052.4 | 0.76358575 | 4.39E-104 | postive |
| NLRP1 | AC245052.4 | 0.65629629 | 1.05E-67 | postive |
| PJVK | AC245052.4 | 0.53147316 | 1.27E-40 | postive |
| PLCG1 | AC245052.4 | 0.5284761 | 4.18E-40 | postive |
| GSDMB | AL354892.3 | 0.60126337 | 2.75E-54 | postive |
| GSDMB | AL117209.1 | 0.65559738 | 1.62E-67 | postive |
| NLRP1 | AL117209.1 | 0.53300646 | 6.86E-41 | postive |
| PJVK | AL117209.1 | 0.52839273 | 4.32E-40 | postive |
| GSDMB | AL031846.2 | 0.53160983 | 1.20E-40 | postive |
| NLRP1 | AL031846.2 | 0.56693266 | 3.70E-47 | postive |
| NOD1 | AL031846.2 | 0.54137217 | 2.27E-42 | postive |
| GSDMB | COA6-AS1 | 0.55698547 | 3.03E-45 | postive |
| PJVK | COA6-AS1 | 0.62671167 | 3.71E-60 | postive |
| GSDMB | AC009061.2 | 0.51448255 | 9.34E-38 | postive |
| GSDMB | LINC01252 | 0.51467848 | 8.67E-38 | postive |
| GSDMB | AC074117.1 | 0.79992568 | 3.46E-121 | postive |
| NLRP1 | AC074117.1 | 0.56140367 | 4.36E-46 | postive |
| PJVK | AC074117.1 | 0.65809454 | 3.44E-68 | postive |
| PLCG1 | AC074117.1 | 0.54240925 | 1.48E-42 | postive |
| CASP1 | PSMB8-AS1 | 0.58284414 | 2.34E-50 | postive |
| NOD2 | PSMB8-AS1 | 0.5198496 | 1.21E-38 | postive |
| SCAF11 | ANKRD10-IT1 | 0.50132292 | 1.21E-35 | postive |
| GSDMB | AC005840.2 | 0.6448346 | 1.11E-64 | postive |
| GSDMB | AL353708.3 | 0.57008435 | 8.87E-48 | postive |
| PJVK | AL353708.3 | 0.56827307 | 2.02E-47 | postive |
| GSDMB | AC092119.2 | 0.71328743 | 6.17E-85 | postive |
| PJVK | AC092119.2 | 0.58985078 | 8.01E-52 | postive |
| GSDMB | ZKSCAN2-DT | 0.73502696 | 1.13E-92 | postive |
| NLRP1 | ZKSCAN2-DT | 0.54543254 | 4.20E-43 | postive |
| PJVK | ZKSCAN2-DT | 0.6162891 | 1.09E-57 | postive |
| PJVK | HDAC4-AS1 | 0.50913831 | 6.90E-37 | postive |
| GSDMB | Z69706.1 | 0.58224028 | 3.12E-50 | postive |
| NLRP1 | Z69706.1 | 0.51756547 | 2.90E-38 | postive |
| GSDMB | AC009120.2 | 0.73699801 | 2.06E-93 | postive |
| NLRP1 | AC009120.2 | 0.54203508 | 1.73E-42 | postive |
| PJVK | AC009120.2 | 0.64374116 | 2.12E-64 | postive |
| SCAF11 | AC104779.1 | 0.53145573 | 1.28E-40 | postive |
| GSDMB | ADAMTSL4-AS2 | 0.53656108 | 1.63E-41 | postive |
| NLRP1 | ADAMTSL4-AS2 | 0.5752272 | 8.36E-49 | postive |
| NOD1 | ADAMTSL4-AS2 | 0.51947253 | 1.40E-38 | postive |
| GSDMB | AC084018.1 | 0.65460242 | 3.00E-67 | postive |
| NLRP1 | AC084018.1 | 0.61549382 | 1.67E-57 | postive |
| PLCG1 | AC084018.1 | 0.54841581 | 1.20E-43 | postive |
| GSDME | LNCSRLR | 0.50413484 | 4.35E-36 | postive |
| GSDMB | AL021707.3 | 0.67381887 | 1.37E-72 | postive |
| NLRP1 | AL021707.3 | 0.65186118 | 1.61E-66 | postive |
| PLCG1 | AL021707.3 | 0.54345723 | 9.58E-43 | postive |
| GSDMB | AL031775.2 | 0.51996882 | 1.15E-38 | postive |
| GSDMB | AC015961.2 | 0.53959979 | 4.72E-42 | postive |
| GSDMB | AC008655.2 | 0.50758803 | 1.22E-36 | postive |
| GSDMB | AC015849.3 | 0.54170202 | 1.98E-42 | postive |
| TIRAP | FAM111A-DT | 0.51645913 | 4.41E-38 | postive |
| GSDMB | AL021707.6 | 0.75383597 | 5.17E-100 | postive |
| NLRP1 | AL021707.6 | 0.60013781 | 4.86E-54 | postive |
| PJVK | AL021707.6 | 0.59608643 | 3.71E-53 | postive |
| SCAF11 | MIR29B2CHG | 0.50215171 | 8.95E-36 | postive |
| NOD1 | AC027601.2 | 0.51987206 | 1.20E-38 | postive |
| PLCG1 | AC027601.2 | 0.52014016 | 1.08E-38 | postive |
| GSDMB | MINCR | 0.51845788 | 2.06E-38 | postive |
| PJVK | MINCR | 0.57254486 | 2.88E-48 | postive |
| PJVK | AC025265.1 | 0.54917416 | 8.68E-44 | postive |
| GSDMB | NCBP2-AS1 | 0.55320858 | 1.55E-44 | postive |
| CASP5 | AC008750.1 | 0.52602837 | 1.10E-39 | postive |
| GSDMB | AC008750.1 | 0.58631167 | 4.45E-51 | postive |
| NLRP1 | AC008750.1 | 0.55867965 | 1.44E-45 | postive |
| NOD2 | AC008750.1 | 0.50233068 | 8.39E-36 | postive |
| GSDMB | AC127024.6 | 0.59185959 | 3.00E-52 | postive |
| PJVK | AC127024.6 | 0.63917457 | 3.10E-63 | postive |
| GSDMB | AC024361.3 | 0.69046746 | 1.48E-77 | postive |
| NLRP1 | AC024361.3 | 0.62510252 | 9.05E-60 | postive |
| PLCG1 | AC024361.3 | 0.55038322 | 5.19E-44 | postive |
| AIM2 | LINC00426 | 0.58053524 | 6.98E-50 | postive |
| CASP1 | LINC00426 | 0.60344635 | 9.03E-55 | postive |
| CASP4 | LINC00426 | 0.57510678 | 8.84E-49 | postive |
| CASP5 | LINC00426 | 0.52000823 | 1.14E-38 | postive |
| GSDMB | LINC00426 | 0.55141808 | 3.34E-44 | postive |
| NOD2 | LINC00426 | 0.71714939 | 2.95E-86 | postive |
| GSDMB | AC092757.2 | 0.51307364 | 1.59E-37 | postive |
| SCAF11 | AP001893.1 | 0.5676644 | 2.66E-47 | postive |
| GSDMB | AC006435.2 | 0.74115684 | 5.39E-95 | postive |
| NLRP1 | AC006435.2 | 0.60138556 | 2.58E-54 | postive |
| PJVK | AC006435.2 | 0.53678239 | 1.49E-41 | postive |
| PLCG1 | AC006435.2 | 0.5105746 | 4.05E-37 | postive |
| GSDMB | AC010319.4 | 0.72730838 | 7.69E-90 | postive |
| NLRP1 | AC010319.4 | 0.57088517 | 6.16E-48 | postive |
| PJVK | AC010319.4 | 0.51661855 | 4.15E-38 | postive |
| GSDMB | ODF2-AS1 | 0.50454147 | 3.75E-36 | postive |
| GSDMB | AC095057.3 | 0.65902811 | 1.92E-68 | postive |
| PJVK | AC095057.3 | 0.73589838 | 5.33E-93 | postive |
| IL18 | MCF2L-AS1 | 0.58894616 | 1.24E-51 | postive |
| GSDMB | AL512652.1 | 0.63116648 | 3.06E-61 | postive |
| PJVK | AL512652.1 | 0.60204962 | 1.84E-54 | postive |
| NOD1 | HID1-AS1 | 0.52795547 | 5.13E-40 | postive |
| PLCG1 | HID1-AS1 | 0.52213519 | 5.00E-39 | postive |
| GSDMB | AC025171.4 | 0.66209611 | 2.77E-69 | postive |
| PJVK | AC025171.4 | 0.63161909 | 2.37E-61 | postive |
| NOD1 | AC007743.1 | 0.54253542 | 1.40E-42 | postive |
| GSDMB | LINC01355 | 0.69756773 | 8.90E-80 | postive |
| NLRP1 | LINC01355 | 0.55727473 | 2.67E-45 | postive |
| PJVK | LINC01355 | 0.66842112 | 4.76E-71 | postive |
| GSDMB | AL135999.1 | 0.78160837 | 3.73E-112 | postive |
| NLRP1 | AL135999.1 | 0.69690429 | 1.45E-79 | postive |
| PJVK | AL135999.1 | 0.59252166 | 2.17E-52 | postive |
| PLCG1 | AL135999.1 | 0.57605469 | 5.69E-49 | postive |
| GSDMB | AC093726.2 | 0.56009344 | 7.76E-46 | postive |
| PJVK | AC093726.2 | 0.52555913 | 1.32E-39 | postive |
| SCAF11 | TTN-AS1 | 0.51018077 | 4.68E-37 | postive |
| GSDMB | CTBP1-AS | 0.65438319 | 3.44E-67 | postive |
| NLRP1 | CTBP1-AS | 0.64404364 | 1.78E-64 | postive |
| NOD1 | CTBP1-AS | 0.52125162 | 7.04E-39 | postive |
| PLCG1 | CTBP1-AS | 0.53935803 | 5.21E-42 | postive |
| PJVK | AC005261.1 | 0.52607958 | 1.07E-39 | postive |
| GSDMB | AC010719.1 | 0.56451624 | 1.09E-46 | postive |
| GSDMB | LINC02019 | 0.71109265 | 3.40E-84 | postive |
| NLRP1 | LINC02019 | 0.59689435 | 2.48E-53 | postive |
| PJVK | LINC02019 | 0.53304057 | 6.77E-41 | postive |
| GSDMB | AC021078.1 | 0.53599543 | 2.05E-41 | postive |
| GSDMB | AC104463.2 | 0.58176868 | 3.90E-50 | postive |
| PJVK | AC104463.2 | 0.52469464 | 1.85E-39 | postive |
| IL18 | SLC25A5-AS1 | 0.56478955 | 9.67E-47 | postive |
| GSDMB | AP003392.1 | 0.5108814 | 3.61E-37 | postive |
| NOD1 | AP003392.1 | 0.52327145 | 3.22E-39 | postive |
| GSDMB | PICART1 | 0.52297467 | 3.61E-39 | postive |
| GSDMB | AC099791.2 | 0.5012647 | 1.23E-35 | postive |
| PJVK | AC099791.2 | 0.60730471 | 1.24E-55 | postive |
| SCAF11 | AL442125.2 | 0.51647699 | 4.38E-38 | postive |
| SCAF11 | AL049840.2 | 0.53253463 | 8.29E-41 | postive |
| NOD1 | DCUN1D2-AS | 0.53849444 | 7.42E-42 | postive |
| IL18 | CA3-AS1 | 0.59170375 | 3.24E-52 | postive |
| GSDMB | AP000442.1 | 0.63878786 | 3.88E-63 | postive |
| PJVK | AP000442.1 | 0.51788932 | 2.56E-38 | postive |
| GSDMB | AC087239.1 | 0.57387951 | 1.56E-48 | postive |
| PJVK | AC087239.1 | 0.54880028 | 1.02E-43 | postive |
| GSDMB | AC020907.4 | 0.80263076 | 1.33E-122 | postive |
| NLRP1 | AC020907.4 | 0.70959813 | 1.08E-83 | postive |
| PJVK | AC020907.4 | 0.5360626 | 2.00E-41 | postive |
| PLCG1 | AC020907.4 | 0.62989945 | 6.24E-61 | postive |
| GSDMB | AL731571.1 | 0.5298055 | 2.47E-40 | postive |
| NLRP1 | AL731571.1 | 0.60276681 | 1.28E-54 | postive |
| NOD1 | AL731571.1 | 0.56239949 | 2.80E-46 | postive |
| PLCG1 | AL731571.1 | 0.55595405 | 4.74E-45 | postive |
| GSDMB | AC007938.3 | 0.59287425 | 1.82E-52 | postive |
| PJVK | AC007938.3 | 0.56943124 | 1.19E-47 | postive |
| SCAF11 | AL391001.1 | 0.50990215 | 5.20E-37 | postive |
| SCAF11 | AP001001.1 | 0.51427629 | 1.01E-37 | postive |
| GSDMB | PSPC1-AS2 | 0.56754663 | 2.80E-47 | postive |
| PJVK | PSPC1-AS2 | 0.52574179 | 1.23E-39 | postive |
| NOD1 | AL049840.3 | 0.62523591 | 8.40E-60 | postive |
| PLCG1 | AL049840.3 | 0.50026899 | 1.77E-35 | postive |
| SCAF11 | AL049840.3 | 0.51086969 | 3.62E-37 | postive |
| SCAF11 | AC068790.4 | 0.51452183 | 9.20E-38 | postive |
| GSDMB | AC003102.1 | 0.77202908 | 8.99E-108 | postive |
| NLRP1 | AC003102.1 | 0.62894738 | 1.07E-60 | postive |
| PJVK | AC003102.1 | 0.64506719 | 9.67E-65 | postive |
| PLCG1 | AC003102.1 | 0.503497 | 5.49E-36 | postive |
| GSDMB | AC016722.2 | 0.50652211 | 1.81E-36 | postive |
| GSDMB | AP006623.1 | 0.60287787 | 1.21E-54 | postive |
| GSDMB | C1orf220 | 0.63705149 | 1.06E-62 | postive |
| GPX4 | FLJ20021 | 0.54335074 | 1.00E-42 | postive |
| SCAF11 | AC007637.1 | 0.59027463 | 6.51E-52 | postive |
| GSDMB | AP4B1-AS1 | 0.68298392 | 2.78E-75 | postive |
| NLRP1 | AP4B1-AS1 | 0.60040952 | 4.23E-54 | postive |
| PJVK | AP4B1-AS1 | 0.52468671 | 1.85E-39 | postive |
| GSDMB | SLFNL1-AS1 | 0.53948853 | 4.94E-42 | postive |
| SCAF11 | LINC00630 | 0.65782388 | 4.07E-68 | postive |
| IL18 | AC148477.4 | 0.59282511 | 1.87E-52 | postive |
| GSDMB | AL354836.1 | 0.68604344 | 3.34E-76 | postive |
| NLRP1 | AL354836.1 | 0.55737948 | 2.55E-45 | postive |
| PJVK | AL354836.1 | 0.51704858 | 3.53E-38 | postive |
| IL18 | PART1 | 0.56522615 | 7.95E-47 | postive |
| GSDMB | AL136295.6 | 0.50423711 | 4.19E-36 | postive |
| PJVK | AL136295.6 | 0.51436344 | 9.76E-38 | postive |
| GSDMB | AC093484.4 | 0.54608492 | 3.19E-43 | postive |
| NLRP1 | AL355075.2 | 0.50022739 | 1.79E-35 | postive |
| NOD1 | AL355075.2 | 0.51756896 | 2.89E-38 | postive |
| SCAF11 | OIP5-AS1 | 0.76119859 | 4.54E-103 | postive |
| GSDMB | AL049780.1 | 0.67009638 | 1.59E-71 | postive |
| NLRP1 | AL049780.1 | 0.56470206 | 1.01E-46 | postive |
| PJVK | AL049780.1 | 0.57073798 | 6.58E-48 | postive |
| GSDMB | AC025766.1 | 0.56055993 | 6.32E-46 | postive |
| GSDMB | SEMA3F-AS1 | 0.6659724 | 2.32E-70 | postive |
| NLRP1 | SEMA3F-AS1 | 0.68186088 | 6.02E-75 | postive |
| NOD1 | SEMA3F-AS1 | 0.55640401 | 3.90E-45 | postive |
| PLCG1 | SEMA3F-AS1 | 0.57502243 | 9.19E-49 | postive |
| GSDMB | AC145285.6 | 0.68711208 | 1.58E-76 | postive |
| NLRP1 | AC145285.6 | 0.52653619 | 8.98E-40 | postive |
| PJVK | AC145285.6 | 0.5828693 | 2.31E-50 | postive |
| GSDMB | AC027796.4 | 0.80959675 | 2.41E-126 | postive |
| NLRP1 | AC027796.4 | 0.60571923 | 2.81E-55 | postive |
| PJVK | AC027796.4 | 0.59457199 | 7.87E-53 | postive |
| PLCG1 | AC027796.4 | 0.53366209 | 5.27E-41 | postive |
| GSDMB | AL161935.1 | 0.52435028 | 2.11E-39 | postive |
| NOD1 | AC021739.2 | 0.50670307 | 1.70E-36 | postive |
| SCAF11 | NADK2-AS1 | 0.56120465 | 4.76E-46 | postive |
| AIM2 | AC007728.2 | 0.54401738 | 7.59E-43 | postive |
| CASP1 | AC007728.2 | 0.6656472 | 2.86E-70 | postive |
| CASP4 | AC007728.2 | 0.56424015 | 1.24E-46 | postive |
| CASP5 | AC007728.2 | 0.5436952 | 8.67E-43 | postive |
| NOD2 | AC007728.2 | 0.78021024 | 1.68E-111 | postive |
| CASP1 | AC243829.4 | 0.57855153 | 1.77E-49 | postive |
| NOD2 | AC243829.4 | 0.63708602 | 1.04E-62 | postive |
| CASP1 | NCK1-DT | 0.58394428 | 1.38E-50 | postive |
| NLRC4 | NCK1-DT | 0.562315 | 2.91E-46 | postive |
| NOD2 | NCK1-DT | 0.51152362 | 2.84E-37 | postive |
| TIRAP | UGDH-AS1 | 0.61669863 | 8.77E-58 | postive |
| GSDMB | AC062037.2 | 0.56929197 | 1.27E-47 | postive |
| GSDMB | AC005387.2 | 0.78267005 | 1.18E-112 | postive |
| NLRP1 | AC005387.2 | 0.63009326 | 5.60E-61 | postive |
| PJVK | AC005387.2 | 0.56649012 | 4.51E-47 | postive |
| PLCG1 | AC005387.2 | 0.52710388 | 7.18E-40 | postive |
| AIM2 | AC099850.3 | 0.55802642 | 1.92E-45 | postive |
| SCAF11 | AC012557.1 | 0.5462077 | 3.03E-43 | postive |
| SCAF11 | AL121672.1 | 0.53335407 | 5.97E-41 | postive |
| GSDMB | ZNNT1 | 0.58769302 | 2.28E-51 | postive |
| GSDMB | AC004687.1 | 0.64629185 | 4.66E-65 | postive |
| NLRP1 | AC004687.1 | 0.56128391 | 4.59E-46 | postive |
| SCAF11 | ITPRIP-AS1 | 0.51399485 | 1.12E-37 | postive |
| SCAF11 | AC090198.1 | 0.6178495 | 4.73E-58 | postive |
| NLRP1 | AC079921.2 | 0.51842396 | 2.09E-38 | postive |
| NOD1 | AC079921.2 | 0.60561553 | 2.97E-55 | postive |
| PLCG1 | AC079921.2 | 0.55250324 | 2.10E-44 | postive |
| SCAF11 | AC090739.1 | 0.50383212 | 4.86E-36 | postive |
| GSDMB | AC018653.3 | 0.65693165 | 7.10E-68 | postive |
| IL18 | AC011523.1 | 0.5925726 | 2.11E-52 | postive |
| GSDMB | AC018926.2 | 0.55024092 | 5.52E-44 | postive |
| SCAF11 | AP000866.1 | 0.52744613 | 6.27E-40 | postive |
| TIRAP | AP000866.1 | 0.50696623 | 1.54E-36 | postive |
| IL18 | AC007342.4 | 0.64091698 | 1.12E-63 | postive |
| IL18 | AL049870.3 | 0.52635043 | 9.66E-40 | postive |
| GSDMB | THUMPD3-AS1 | 0.61143359 | 1.44E-56 | postive |
| PJVK | THUMPD3-AS1 | 0.57168054 | 4.28E-48 | postive |
| TIRAP | CTBP1-DT | 0.55146471 | 3.27E-44 | postive |
| GSDMB | AC127024.5 | 0.59767349 | 1.68E-53 | postive |
| PJVK | AC127024.5 | 0.55806102 | 1.89E-45 | postive |
| GSDMB | AP000254.2 | 0.548289 | 1.26E-43 | postive |
| PJVK | AP000254.2 | 0.5359607 | 2.08E-41 | postive |
| GSDMB | AL139349.1 | 0.59857817 | 1.07E-53 | postive |
| SCAF11 | AC011939.2 | 0.526146 | 1.05E-39 | postive |
| PJVK | AC025162.2 | 0.52086967 | 8.15E-39 | postive |
| SCAF11 | MIRLET7A1HG | 0.53823075 | 8.26E-42 | postive |
| GSDMB | AC017083.1 | 0.69545393 | 4.15E-79 | postive |
| NLRP1 | AC017083.1 | 0.51313531 | 1.55E-37 | postive |
| PJVK | AC017083.1 | 0.60515916 | 3.75E-55 | postive |
| GSDMB | AC004908.1 | 0.52027157 | 1.03E-38 | postive |
| CASP1 | TSPOAP1-AS1 | 0.51154663 | 2.81E-37 | postive |
| NOD2 | TSPOAP1-AS1 | 0.66890531 | 3.47E-71 | postive |
| GSDMB | AC108673.3 | 0.56195535 | 3.41E-46 | postive |
| PJVK | AC108673.3 | 0.52116437 | 7.28E-39 | postive |
| GSDMB | PXN-AS1 | 0.61372806 | 4.27E-57 | postive |
| GSDMB | AC109460.2 | 0.67315416 | 2.13E-72 | postive |
| NLRP1 | AC109460.2 | 0.61099686 | 1.81E-56 | postive |
| NOD1 | AC109460.2 | 0.54731239 | 1.91E-43 | postive |
| PLCG1 | AC109460.2 | 0.53815769 | 8.51E-42 | postive |
| SCAF11 | AL035071.2 | 0.54759564 | 1.69E-43 | postive |
| SCAF11 | AC007878.1 | 0.54616805 | 3.09E-43 | postive |
| GSDMB | LINC01138 | 0.66533926 | 3.49E-70 | postive |
| PJVK | LINC01138 | 0.51251237 | 1.96E-37 | postive |
| SCAF11 | AL137003.1 | 0.55884647 | 1.34E-45 | postive |
| GSDMB | AC114956.1 | 0.50846461 | 8.86E-37 | postive |
| GSDMB | TPT1-AS1 | 0.60653341 | 1.85E-55 | postive |
| NLRP1 | TPT1-AS1 | 0.51156653 | 2.79E-37 | postive |
| PJVK | TPT1-AS1 | 0.55412358 | 1.05E-44 | postive |
| CASP1 | USP30-AS1 | 0.63532833 | 2.86E-62 | postive |
| CASP4 | USP30-AS1 | 0.5782019 | 2.09E-49 | postive |
| NOD2 | USP30-AS1 | 0.67522717 | 5.36E-73 | postive |
| PYCARD | USP30-AS1 | 0.59249931 | 2.19E-52 | postive |
| SCAF11 | AC007319.1 | 0.5125628 | 1.92E-37 | postive |
| GSDMB | RUFY1-AS1 | 0.54850008 | 1.15E-43 | postive |
| NLRP1 | RUFY1-AS1 | 0.50988767 | 5.22E-37 | postive |
| NOD1 | RUFY1-AS1 | 0.53029127 | 2.03E-40 | postive |
| GSDMB | ARHGEF2-AS2 | 0.73635473 | 3.60E-93 | postive |
| PJVK | ARHGEF2-AS2 | 0.60397427 | 6.90E-55 | postive |
| GSDMB | AP001628.1 | 0.55304957 | 1.66E-44 | postive |
| GSDMB | AP001793.1 | 0.50149717 | 1.13E-35 | postive |
| NLRP1 | AP001793.1 | 0.5287773 | 3.71E-40 | postive |
| NOD1 | AP001793.1 | 0.52778088 | 5.50E-40 | postive |
| PJVK | AL022341.1 | 0.5023973 | 8.19E-36 | postive |
| GSDMB | HCG25 | 0.54839781 | 1.21E-43 | postive |
| SCAF11 | ARAP1-AS2 | 0.54584021 | 3.54E-43 | postive |
| NOD1 | PAXBP1-AS1 | 0.53747018 | 1.13E-41 | postive |
| SCAF11 | PAXBP1-AS1 | 0.5232067 | 3.30E-39 | postive |
| SCAF11 | EBLN3P | 0.62295341 | 2.95E-59 | postive |
| SCAF11 | OTUD6B-AS1 | 0.54996468 | 6.20E-44 | postive |
| NLRP1 | AC124045.1 | 0.55649466 | 3.75E-45 | postive |
| GSDMB | AC010538.1 | 0.57180024 | 4.05E-48 | postive |
| SCAF11 | SNHG14 | 0.55598202 | 4.68E-45 | postive |
| GSDMB | AC097641.2 | 0.64633457 | 4.54E-65 | postive |
| PJVK | AC097641.2 | 0.57198983 | 3.71E-48 | postive |
| CASP1 | TFAP2E-AS1 | 0.56958582 | 1.11E-47 | postive |
| CASP4 | TFAP2E-AS1 | 0.55069048 | 4.56E-44 | postive |
| GSDMB | TFAP2E-AS1 | 0.58700198 | 3.19E-51 | postive |
| NLRP1 | TFAP2E-AS1 | 0.50385742 | 4.81E-36 | postive |
| NOD2 | TFAP2E-AS1 | 0.63563944 | 2.39E-62 | postive |
| SCAF11 | AC011815.1 | 0.53360846 | 5.39E-41 | postive |
| IL18 | AC013472.1 | 0.52704157 | 7.36E-40 | postive |
| SCAF11 | SUCLG2-AS1 | 0.51218659 | 2.21E-37 | postive |
| IL18 | AC103563.7 | 0.68583134 | 3.87E-76 | postive |
| SCAF11 | AC107027.3 | 0.59149584 | 3.58E-52 | postive |
| SCAF11 | AC108693.2 | 0.53975614 | 4.42E-42 | postive |
| GSDMB | TRAPPC12-AS1 | 0.54838578 | 1.21E-43 | postive |
| GSDMB | AC012615.6 | 0.743196 | 8.80E-96 | postive |
| NLRP1 | AC012615.6 | 0.65412058 | 4.04E-67 | postive |
| PJVK | AC012615.6 | 0.50499861 | 3.17E-36 | postive |
| PLCG1 | AC012615.6 | 0.54255085 | 1.40E-42 | postive |
| GSDMB | MIR3936HG | 0.56059838 | 6.21E-46 | postive |
| GPX4 | YTHDF3-AS1 | 0.52420704 | 2.23E-39 | postive |
| GSDMD | YTHDF3-AS1 | 0.54491042 | 5.23E-43 | postive |
| NLRP1 | AC018695.4 | 0.50160938 | 1.09E-35 | postive |
| NOD1 | AC018695.4 | 0.50902856 | 7.19E-37 | postive |
| GSDMB | AC008735.4 | 0.64056426 | 1.38E-63 | postive |
| NLRP1 | AC008735.4 | 0.5890862 | 1.16E-51 | postive |
| PJVK | AC008735.4 | 0.51637144 | 4.56E-38 | postive |
| CASP9 | LINC01606 | 0.51310414 | 1.57E-37 | postive |
| GSDMB | AL731567.1 | 0.65529526 | 1.96E-67 | postive |
| NOD2 | AL731567.1 | 0.54192933 | 1.81E-42 | postive |
| PJVK | AC020910.5 | 0.57687371 | 3.89E-49 | postive |
| NLRP1 | AC011466.1 | 0.52436344 | 2.10E-39 | postive |
| NOD1 | AC011466.1 | 0.5075748 | 1.23E-36 | postive |
| GSDMB | AC009120.3 | 0.54445353 | 6.32E-43 | postive |
| SCAF11 | WASHC5-AS1 | 0.51213781 | 2.26E-37 | postive |
| SCAF11 | AC068790.3 | 0.51437882 | 9.71E-38 | postive |
| SCAF11 | AC027514.2 | 0.57242653 | 3.04E-48 | postive |
| GSDMB | PKD1P6-NPIPP1 | 0.66258194 | 2.03E-69 | postive |
| NLRP1 | PKD1P6-NPIPP1 | 0.55666895 | 3.47E-45 | postive |
| PJVK | PKD1P6-NPIPP1 | 0.57638995 | 4.87E-49 | postive |
| PLCG1 | PKD1P6-NPIPP1 | 0.50897847 | 7.32E-37 | postive |
| PJVK | STK24-AS1 | 0.59279194 | 1.90E-52 | postive |
| GSDMB | LINC00539 | 0.57462674 | 1.10E-48 | postive |
| NOD2 | LINC00539 | 0.61370396 | 4.33E-57 | postive |
| SCAF11 | AC068790.6 | 0.55212085 | 2.47E-44 | postive |
| GSDMB | AC008870.2 | 0.74906907 | 4.32E-98 | postive |
| NLRP1 | AC008870.2 | 0.56729202 | 3.14E-47 | postive |
| PJVK | AC008870.2 | 0.57845725 | 1.86E-49 | postive |
| NOD1 | AP000941.1 | 0.53540098 | 2.61E-41 | postive |
| GSDMB | AC103706.1 | 0.55722521 | 2.73E-45 | postive |
| GSDMB | AL513218.1 | 0.7689883 | 2.00E-106 | postive |
| NLRP1 | AL513218.1 | 0.60504149 | 3.99E-55 | postive |
| PJVK | AL513218.1 | 0.59799094 | 1.43E-53 | postive |
| IL18 | HOXB-AS3 | 0.61913517 | 2.36E-58 | postive |
| GSDMB | AC139530.1 | 0.5558323 | 4.99E-45 | postive |
| PJVK | AC139530.1 | 0.5856364 | 6.15E-51 | postive |
| GSDMB | AC084117.1 | 0.61582915 | 1.40E-57 | postive |
| PJVK | AC084117.1 | 0.57438277 | 1.24E-48 | postive |
| GSDMB | AC048341.1 | 0.68074035 | 1.30E-74 | postive |
| NLRP1 | AC048341.1 | 0.55841431 | 1.62E-45 | postive |
| PJVK | AC048341.1 | 0.57713194 | 3.45E-49 | postive |
| IL18 | AC010776.3 | 0.50851114 | 8.71E-37 | postive |
| IL6 | AC092376.2 | 0.50606137 | 2.15E-36 | postive |
| GSDMB | AC106782.5 | 0.59914628 | 8.01E-54 | postive |
| SCAF11 | AC103739.1 | 0.50078372 | 1.47E-35 | postive |
| SCAF11 | AC012404.1 | 0.51943222 | 1.42E-38 | postive |
| CASP1 | LINC02285 | 0.64899185 | 9.21E-66 | postive |
| NLRC4 | LINC02285 | 0.61116485 | 1.65E-56 | postive |
| NLRP3 | LINC02285 | 0.55391983 | 1.14E-44 | postive |
| NOD2 | LINC02285 | 0.50171491 | 1.05E-35 | postive |
| GSDMB | ZNF460-AS1 | 0.61660893 | 9.20E-58 | postive |
| PJVK | ZNF460-AS1 | 0.63130854 | 2.82E-61 | postive |
| SCAF11 | AL161725.1 | 0.58010612 | 8.55E-50 | postive |
| GSDMB | AL031670.1 | 0.57187939 | 3.91E-48 | postive |
| NLRP1 | AL031670.1 | 0.57622373 | 5.26E-49 | postive |
| NOD1 | AL031670.1 | 0.56618241 | 5.18E-47 | postive |
| PLCG1 | AL031670.1 | 0.50918758 | 6.78E-37 | postive |
| GSDMB | AC078906.1 | 0.54001532 | 3.98E-42 | postive |
| PJVK | AC078906.1 | 0.5042617 | 4.15E-36 | postive |
| GSDMB | AC015813.1 | 0.68571557 | 4.19E-76 | postive |
| NLRP1 | AC015813.1 | 0.51758018 | 2.88E-38 | postive |
| PJVK | AC015813.1 | 0.67431212 | 9.87E-73 | postive |
| NLRP1 | AL034550.1 | 0.5397667 | 4.40E-42 | postive |
| NOD1 | AL034550.1 | 0.61038408 | 2.49E-56 | postive |
| PLCG1 | AL034550.1 | 0.53814416 | 8.56E-42 | postive |
| GSDMB | AC007066.2 | 0.54652404 | 2.66E-43 | postive |
| NLRP1 | AC007066.2 | 0.53158283 | 1.21E-40 | postive |
| PJVK | AC007066.2 | 0.5011077 | 1.31E-35 | postive |
| GSDMB | AC245060.5 | 0.52570624 | 1.24E-39 | postive |
| GSDMB | AC104532.2 | 0.57126778 | 5.17E-48 | postive |
| NLRP1 | AC104532.2 | 0.55872719 | 1.41E-45 | postive |
| GSDMB | MCCC1-AS1 | 0.67684638 | 1.81E-73 | postive |
| NLRP1 | MCCC1-AS1 | 0.50098995 | 1.36E-35 | postive |
| PJVK | MCCC1-AS1 | 0.53069631 | 1.73E-40 | postive |
| SCAF11 | AC130895.1 | 0.52252943 | 4.29E-39 | postive |
| NLRP1 | AL049840.4 | 0.55402578 | 1.09E-44 | postive |
| NOD1 | AL049840.4 | 0.6139214 | 3.86E-57 | postive |
| PLCG1 | AL049840.4 | 0.5391824 | 5.60E-42 | postive |
| GSDMB | AL022238.2 | 0.61058186 | 2.25E-56 | postive |
| NLRP1 | AL022238.2 | 0.58826654 | 1.73E-51 | postive |
| PLCG1 | AL022238.2 | 0.52677748 | 8.16E-40 | postive |
| GSDMB | U91328.3 | 0.53730302 | 1.21E-41 | postive |
| PJVK | U91328.3 | 0.54484151 | 5.38E-43 | postive |
| SCAF11 | NPTN-IT1 | 0.56349011 | 1.73E-46 | postive |
| GSDMB | AC127521.1 | 0.5555498 | 5.64E-45 | postive |
| GSDMB | AL158212.2 | 0.66800846 | 6.22E-71 | postive |
| NLRP1 | AL158212.2 | 0.56116522 | 4.84E-46 | postive |
| PJVK | AL158212.2 | 0.55049905 | 4.94E-44 | postive |
| SCAF11 | AC242426.2 | 0.58985143 | 8.01E-52 | postive |
| NLRP1 | AC005740.4 | 0.62048072 | 1.14E-58 | postive |
| NOD1 | AC005740.4 | 0.64721153 | 2.69E-65 | postive |
| PLCG1 | AC005740.4 | 0.59199626 | 2.80E-52 | postive |
| GSDMB | ZNF32-AS1 | 0.66982845 | 1.90E-71 | postive |
| PJVK | ZNF32-AS1 | 0.70664843 | 1.03E-82 | postive |
| GSDMB | U62317.1 | 0.74057304 | 9.03E-95 | postive |
| NLRP1 | U62317.1 | 0.57549821 | 7.37E-49 | postive |
| GSDMB | AL390728.5 | 0.64498238 | 1.02E-64 | postive |
| NLRP1 | AL390728.5 | 0.52706677 | 7.29E-40 | postive |
| PJVK | AL390728.5 | 0.63333287 | 8.94E-62 | postive |
| SCAF11 | GNG12-AS1 | 0.57634484 | 4.97E-49 | postive |
| PJVK | AC026412.3 | 0.59438851 | 8.62E-53 | postive |
| SCAF11 | RAB33B-AS1 | 0.54097096 | 2.68E-42 | postive |
| IL6 | MIAT | 0.63834058 | 5.03E-63 | postive |
| GSDMB | INE1 | 0.66052447 | 7.48E-69 | postive |
| NLRP1 | INE1 | 0.57466834 | 1.08E-48 | postive |
| GSDMB | AC005840.4 | 0.62816908 | 1.65E-60 | postive |
| PJVK | AC005840.4 | 0.51357184 | 1.32E-37 | postive |
| NLRP3 | AC243960.3 | 0.54302868 | 1.14E-42 | postive |
| GSDMB | AL513477.2 | 0.50493227 | 3.25E-36 | postive |
| GSDMB | AC010326.3 | 0.7530893 | 1.04E-99 | postive |
| NLRP1 | AC010326.3 | 0.54230319 | 1.55E-42 | postive |
| PJVK | AC010326.3 | 0.55761051 | 2.30E-45 | postive |
| GSDMB | AC079174.2 | 0.63328713 | 9.18E-62 | postive |
| PJVK | AC079174.2 | 0.67819357 | 7.31E-74 | postive |
| SCAF11 | AL139022.2 | 0.5113866 | 2.99E-37 | postive |
| GSDMB | AC026333.4 | 0.55160976 | 3.08E-44 | postive |
| PJVK | AC026333.4 | 0.57830842 | 1.99E-49 | postive |
| NLRP1 | AL135960.1 | 0.54184086 | 1.87E-42 | postive |
| NOD1 | AL135960.1 | 0.58060313 | 6.76E-50 | postive |
| PLCG1 | AL135960.1 | 0.53418832 | 4.27E-41 | postive |
| AIM2 | LINC02084 | 0.50669204 | 1.70E-36 | postive |
| GSDMB | LINC02084 | 0.50515977 | 2.99E-36 | postive |
| NOD2 | LINC02084 | 0.58944131 | 9.78E-52 | postive |
| GSDMB | GEMIN7-AS1 | 0.64145907 | 8.15E-64 | postive |
| PJVK | GEMIN7-AS1 | 0.62118819 | 7.75E-59 | postive |
| GSDMB | AL132989.1 | 0.53246025 | 8.55E-41 | postive |
| NLRP1 | AL132989.1 | 0.53745117 | 1.14E-41 | postive |
| NOD1 | AL132989.1 | 0.56632672 | 4.85E-47 | postive |
| PJVK | AC009159.3 | 0.60413693 | 6.34E-55 | postive |
| SCAF11 | SP2-AS1 | 0.50493861 | 3.24E-36 | postive |
| GSDMB | AC009318.4 | 0.54286188 | 1.23E-42 | postive |
| PJVK | AC009318.4 | 0.59210083 | 2.66E-52 | postive |
| PYCARD | AC147067.1 | 0.52434349 | 2.12E-39 | postive |
| SCAF11 | AL355916.2 | 0.57982987 | 9.74E-50 | postive |
| IL18 | LINC02608 | 0.56708936 | 3.44E-47 | postive |
| PJVK | AC010997.4 | 0.51822494 | 2.25E-38 | postive |
| NLRC4 | AC138207.5 | 0.52805295 | 4.94E-40 | postive |
| NOD2 | AC138207.5 | 0.52240424 | 4.50E-39 | postive |
| SCAF11 | ARRDC3-AS1 | 0.53656677 | 1.63E-41 | postive |
| SCAF11 | AC114763.1 | 0.5275473 | 6.03E-40 | postive |
| SCAF11 | SOS1-IT1 | 0.59612669 | 3.63E-53 | postive |
| GSDMB | AC129510.1 | 0.74099816 | 6.20E-95 | postive |
| NLRP1 | AC129510.1 | 0.59448837 | 8.20E-53 | postive |
| PJVK | AC129510.1 | 0.57198764 | 3.72E-48 | postive |
| GSDMB | AC025165.4 | 0.53496515 | 3.12E-41 | postive |
| NOD1 | AL355803.1 | 0.60294992 | 1.16E-54 | postive |
| PLCG1 | AL355803.1 | 0.58077893 | 6.22E-50 | postive |
| IL18 | AC103563.2 | 0.56080665 | 5.67E-46 | postive |
| SCAF11 | AC090948.2 | 0.58151559 | 4.39E-50 | postive |
| GSDMB | AC026471.4 | 0.71335183 | 5.87E-85 | postive |
| PJVK | AC026471.4 | 0.64802844 | 1.65E-65 | postive |
| GSDMB | FOXD2-AS1 | 0.50093851 | 1.39E-35 | postive |
| NLRP1 | FOXD2-AS1 | 0.50803883 | 1.04E-36 | postive |
| GSDMB | SNHG3 | 0.61389424 | 3.91E-57 | postive |
| GSDMB | AC074212.1 | 0.63615551 | 1.78E-62 | postive |
| GSDMB | AC106795.2 | 0.61623715 | 1.12E-57 | postive |
| IL6 | AC015819.2 | 0.50916883 | 6.82E-37 | postive |
| GSDMB | VPS9D1-AS1 | 0.68820336 | 7.35E-77 | postive |
| SCAF11 | AL109614.1 | 0.5727176 | 2.66E-48 | postive |
| GSDMB | AL031186.1 | 0.68023514 | 1.83E-74 | postive |
| NLRP1 | AL031186.1 | 0.56736309 | 3.04E-47 | postive |
| GSDMB | AC007686.3 | 0.57194917 | 3.78E-48 | postive |
| SCAF11 | AP001486.2 | 0.56909751 | 1.39E-47 | postive |
| GSDMB | AC010761.1 | 0.69526394 | 4.76E-79 | postive |
| PJVK | AC010761.1 | 0.57931247 | 1.24E-49 | postive |
| GSDMB | C2orf49-DT | 0.71063535 | 4.84E-84 | postive |
| NLRP1 | C2orf49-DT | 0.53872383 | 6.75E-42 | postive |
| PJVK | C2orf49-DT | 0.65570059 | 1.52E-67 | postive |
| GSDMB | AC145285.2 | 0.6044546 | 5.39E-55 | postive |
| PJVK | AC145285.2 | 0.53078124 | 1.67E-40 | postive |
| GSDMB | LENG8-AS1 | 0.75478817 | 2.11E-100 | postive |
| NLRP1 | LENG8-AS1 | 0.59644734 | 3.10E-53 | postive |
| PJVK | LENG8-AS1 | 0.54818913 | 1.32E-43 | postive |
| PLCG1 | LENG8-AS1 | 0.52353704 | 2.90E-39 | postive |
| SCAF11 | APOA1-AS | 0.50947064 | 6.10E-37 | postive |
| GSDMB | AL161669.3 | 0.53719121 | 1.26E-41 | postive |
| PJVK | AL161669.3 | 0.52120567 | 7.16E-39 | postive |
| GSDMB | SSBP3-AS1 | 0.54278141 | 1.27E-42 | postive |
| NLRP1 | SSBP3-AS1 | 0.52576038 | 1.22E-39 | postive |
| NOD1 | SSBP3-AS1 | 0.52872103 | 3.79E-40 | postive |
| GSDMB | AC027020.2 | 0.55577202 | 5.13E-45 | postive |
| SCAF11 | AC012181.1 | 0.51463934 | 8.80E-38 | postive |
| GSDMB | AC008105.2 | 0.7804453 | 1.30E-111 | postive |
| NLRP1 | AC008105.2 | 0.61211374 | 1.00E-56 | postive |
| NOD2 | AC008105.2 | 0.52250716 | 4.33E-39 | postive |
| TIRAP | AC018521.6 | 0.52479027 | 1.78E-39 | postive |
| GSDMB | AC114730.3 | 0.73383818 | 3.13E-92 | postive |
| NLRP1 | AC114730.3 | 0.65548557 | 1.74E-67 | postive |
| PJVK | AC114730.3 | 0.50756373 | 1.24E-36 | postive |
| PLCG1 | AC114730.3 | 0.55212232 | 2.47E-44 | postive |
| SCAF11 | AL731568.1 | 0.52240124 | 4.51E-39 | postive |
| GSDMB | IBA57-DT | 0.6384463 | 4.73E-63 | postive |
| NLRP1 | IBA57-DT | 0.53687864 | 1.43E-41 | postive |
| PJVK | IBA57-DT | 0.53322239 | 6.29E-41 | postive |
| GSDMB | AC010809.2 | 0.71566121 | 9.58E-86 | postive |
| NLRP1 | AC010809.2 | 0.55781733 | 2.10E-45 | postive |
| PJVK | AC010809.2 | 0.64149228 | 8.00E-64 | postive |
| GSDMB | AL596223.2 | 0.51254692 | 1.93E-37 | postive |
| GSDMB | AC015660.3 | 0.56006965 | 7.84E-46 | postive |
| GSDMB | LINC00653 | 0.67893574 | 4.42E-74 | postive |
| PJVK | LINC00653 | 0.62214137 | 4.61E-59 | postive |
| IL18 | ATP6V0E2-AS1 | 0.50854559 | 8.60E-37 | postive |
| GSDMB | AP001453.2 | 0.6015503 | 2.37E-54 | postive |
| PJVK | AP001453.2 | 0.53711912 | 1.30E-41 | postive |
| IL18 | AL035661.1 | 0.59019426 | 6.77E-52 | postive |
| GSDMB | AL390066.1 | 0.66503691 | 4.24E-70 | postive |
| NLRP1 | AL390066.1 | 0.52129312 | 6.92E-39 | postive |
| GSDMB | OBSCN-AS1 | 0.60808996 | 8.25E-56 | postive |
| PJVK | OBSCN-AS1 | 0.52865715 | 3.89E-40 | postive |
| GSDMB | DNAJC9-AS1 | 0.56851097 | 1.81E-47 | postive |
| PJVK | DNAJC9-AS1 | 0.59918913 | 7.84E-54 | postive |
| GSDMB | AC018638.7 | 0.54117285 | 2.47E-42 | postive |
| PJVK | AC018638.7 | 0.51402839 | 1.11E-37 | postive |
| GSDMB | ZNF213-AS1 | 0.72694216 | 1.04E-89 | postive |
| NLRP1 | ZNF213-AS1 | 0.52604216 | 1.09E-39 | postive |
| PJVK | ZNF213-AS1 | 0.61969957 | 1.74E-58 | postive |
| GSDMB | HM13-IT1 | 0.77465869 | 5.92E-109 | postive |
| NLRP1 | HM13-IT1 | 0.59037473 | 6.20E-52 | postive |
| PJVK | HM13-IT1 | 0.52815971 | 4.73E-40 | postive |
| SCAF11 | AC018410.1 | 0.50434548 | 4.03E-36 | postive |
| GSDMB | AC104564.3 | 0.72696871 | 1.02E-89 | postive |
| NLRP1 | AC104564.3 | 0.58758788 | 2.40E-51 | postive |
| PJVK | AC104564.3 | 0.58206163 | 3.39E-50 | postive |
| SCAF11 | AC010834.3 | 0.64776685 | 1.93E-65 | postive |
| GSDMB | SH3BP5-AS1 | 0.71781164 | 1.74E-86 | postive |
| NLRP1 | SH3BP5-AS1 | 0.7281158 | 3.93E-90 | postive |
| NOD1 | SH3BP5-AS1 | 0.54329695 | 1.02E-42 | postive |
| PJVK | SH3BP5-AS1 | 0.58505592 | 8.13E-51 | postive |
| PLCG1 | SH3BP5-AS1 | 0.58319381 | 1.98E-50 | postive |
| SCAF11 | AP003059.1 | 0.57014139 | 8.64E-48 | postive |
| CASP1 | AC079015.1 | 0.54743324 | 1.81E-43 | postive |
| NOD2 | AC079015.1 | 0.58395966 | 1.37E-50 | postive |
| SCAF11 | KANSL1L-AS1 | 0.51020084 | 4.65E-37 | postive |
| SCAF11 | AC105389.2 | 0.60827954 | 7.47E-56 | postive |
| GSDMB | AC005306.1 | 0.75290446 | 1.24E-99 | postive |
| NLRP1 | AC005306.1 | 0.68277475 | 3.21E-75 | postive |
| PJVK | AC005306.1 | 0.52006069 | 1.11E-38 | postive |
| PLCG1 | AC005306.1 | 0.51491966 | 7.91E-38 | postive |
| GSDMB | MAP3K14-AS1 | 0.65186462 | 1.61E-66 | postive |
| CASP1 | PTPRN2-AS1 | 0.5849423 | 8.59E-51 | postive |
| CASP4 | PTPRN2-AS1 | 0.52680986 | 8.06E-40 | postive |
| CASP5 | PTPRN2-AS1 | 0.53008473 | 2.21E-40 | postive |
| NLRC4 | PTPRN2-AS1 | 0.64319415 | 2.94E-64 | postive |
| NLRP3 | PTPRN2-AS1 | 0.57723811 | 3.28E-49 | postive |
| NOD2 | PTPRN2-AS1 | 0.64694245 | 3.16E-65 | postive |
| GSDMB | AC005674.1 | 0.60224034 | 1.67E-54 | postive |
| NLRP1 | AC005674.1 | 0.62583796 | 6.02E-60 | postive |
| PJVK | AC005674.1 | 0.5108148 | 3.70E-37 | postive |
| SCAF11 | Z83843.1 | 0.54464912 | 5.83E-43 | postive |
| SCAF11 | AC092279.1 | 0.5027266 | 7.26E-36 | postive |
| PJVK | AC009812.1 | 0.55090416 | 4.16E-44 | postive |
| PJVK | AL138960.1 | 0.56063581 | 6.11E-46 | postive |
| GSDMB | LINC02804 | 0.56581723 | 6.10E-47 | postive |
| SCAF11 | AC024270.3 | 0.54429941 | 6.74E-43 | postive |
| GSDMB | AL031709.1 | 0.58445097 | 1.09E-50 | postive |
| NLRP1 | AL031709.1 | 0.53431127 | 4.06E-41 | postive |
| PLCG1 | AL031709.1 | 0.50394917 | 4.65E-36 | postive |
| SCAF11 | SEPTIN7-DT | 0.53620597 | 1.89E-41 | postive |
| TIRAP | SEPTIN7-DT | 0.51857207 | 1.97E-38 | postive |
| GSDMB | MCM3AP-AS1 | 0.55427898 | 9.78E-45 | postive |
| NOD1 | MCM3AP-AS1 | 0.50408335 | 4.43E-36 | postive |
| GSDMB | AL031714.1 | 0.77143863 | 1.65E-107 | postive |
| NLRP1 | AL031714.1 | 0.68936546 | 3.24E-77 | postive |
| PJVK | AL031714.1 | 0.52050825 | 9.37E-39 | postive |
| PLCG1 | AL031714.1 | 0.61914674 | 2.35E-58 | postive |
| GSDMB | HNRNPD-DT | 0.51706753 | 3.50E-38 | postive |
| PJVK | HNRNPD-DT | 0.6029013 | 1.19E-54 | postive |
| GSDMB | AC087500.1 | 0.63412569 | 5.69E-62 | postive |
| NLRP1 | AC087500.1 | 0.51957922 | 1.34E-38 | postive |
| GSDMB | CD27-AS1 | 0.60236301 | 1.57E-54 | postive |
| PJVK | CD27-AS1 | 0.51054206 | 4.09E-37 | postive |
| GSDMB | AC023908.3 | 0.6591456 | 1.78E-68 | postive |
| PJVK | AC023908.3 | 0.59853799 | 1.09E-53 | postive |
| GSDMB | CEP250-AS1 | 0.58249383 | 2.76E-50 | postive |
| NLRP1 | CEP250-AS1 | 0.51469889 | 8.60E-38 | postive |
| NOD2 | CEP250-AS1 | 0.50268187 | 7.38E-36 | postive |
| GSDMB | AL450998.2 | 0.5478752 | 1.50E-43 | postive |
| GSDMB | AC008735.2 | 0.76483323 | 1.28E-104 | postive |
| NLRP1 | AC008735.2 | 0.67427179 | 1.01E-72 | postive |
| PLCG1 | AC008735.2 | 0.58860949 | 1.46E-51 | postive |
| GSDMB | MIR155HG | 0.58512703 | 7.86E-51 | postive |
| NOD2 | MIR155HG | 0.53659747 | 1.61E-41 | postive |
| GSDMB | AC068620.2 | 0.61244498 | 8.42E-57 | postive |
| PJVK | AC068620.2 | 0.73579301 | 5.84E-93 | postive |
| SCAF11 | KIAA1671-AS1 | 0.50744648 | 1.29E-36 | postive |
| GSDMB | AC027271.1 | 0.52468818 | 1.85E-39 | postive |
| PJVK | AC027271.1 | 0.61737713 | 6.09E-58 | postive |
| IL18 | NCOA7-AS1 | 0.51440315 | 9.62E-38 | postive |
| GSDMB | AP005899.1 | 0.50029176 | 1.75E-35 | postive |
| GSDMB | RRN3P2 | 0.60454967 | 5.13E-55 | postive |
| NLRP1 | RRN3P2 | 0.52838478 | 4.33E-40 | postive |
| NOD1 | AL021368.2 | 0.51881129 | 1.80E-38 | postive |
| SCAF11 | AL021368.2 | 0.54602334 | 3.28E-43 | postive |
| IL18 | AC005281.1 | 0.55710548 | 2.87E-45 | postive |
| GSDMB | AL133215.1 | 0.56365005 | 1.61E-46 | postive |
| GSDMB | PDXDC2P-NPIPB14P | 0.73158572 | 2.13E-91 | postive |
| NLRP1 | PDXDC2P-NPIPB14P | 0.59756683 | 1.77E-53 | postive |
| PJVK | PDXDC2P-NPIPB14P | 0.6270085 | 3.14E-60 | postive |
| GSDMB | AC007497.1 | 0.50693906 | 1.56E-36 | postive |
| PJVK | AC007497.1 | 0.55474573 | 7.99E-45 | postive |
| IL18 | LINC02343 | 0.59856425 | 1.07E-53 | postive |
| GSDMB | AC006064.3 | 0.55246715 | 2.13E-44 | postive |
| GSDMB | AL096865.1 | 0.66608366 | 2.16E-70 | postive |
| NLRP1 | AL096865.1 | 0.58677275 | 3.56E-51 | postive |
| NOD1 | AC092614.1 | 0.54831225 | 1.25E-43 | postive |
| GSDMB | AC020658.5 | 0.53516497 | 2.88E-41 | postive |
| NOD2 | AC020658.5 | 0.50548903 | 2.65E-36 | postive |
| GSDMB | MZF1-AS1 | 0.55017374 | 5.68E-44 | postive |
| GSDMB | AC012645.4 | 0.79887949 | 1.20E-120 | postive |
| NLRP1 | AC012645.4 | 0.64317792 | 2.96E-64 | postive |
| PJVK | AC012645.4 | 0.57145744 | 4.74E-48 | postive |
| PLCG1 | AC012645.4 | 0.515764 | 5.75E-38 | postive |
| CASP1 | LINC01094 | 0.53565205 | 2.36E-41 | postive |
| CASP5 | LINC01094 | 0.63221756 | 1.69E-61 | postive |
| NLRC4 | LINC01094 | 0.69921507 | 2.66E-80 | postive |
| NLRP3 | LINC01094 | 0.5778766 | 2.43E-49 | postive |
| GSDMB | AC132872.1 | 0.66241542 | 2.26E-69 | postive |
| NLRP1 | AC132872.1 | 0.60786266 | 9.28E-56 | postive |
| PJVK | AC132872.1 | 0.61072577 | 2.08E-56 | postive |
| PLCG1 | AC132872.1 | 0.57285705 | 2.49E-48 | postive |
| GSDMB | AL512791.1 | 0.51043899 | 4.25E-37 | postive |
| NLRP1 | AL512791.1 | 0.58127767 | 4.92E-50 | postive |
| NOD1 | AL512791.1 | 0.56377044 | 1.52E-46 | postive |
| PLCG1 | AL512791.1 | 0.54289589 | 1.21E-42 | postive |
| GSDMB | AP006621.2 | 0.64493925 | 1.04E-64 | postive |
| NLRP1 | AP006621.2 | 0.52003033 | 1.13E-38 | postive |
| GSDMB | AC018648.1 | 0.57821688 | 2.08E-49 | postive |
| PJVK | AC018648.1 | 0.57522323 | 8.37E-49 | postive |
| GSDMB | AC084018.2 | 0.54167512 | 2.01E-42 | postive |
| NLRP1 | AC084018.2 | 0.52374382 | 2.68E-39 | postive |
| GSDMB | MYG1-AS1 | 0.70563981 | 2.20E-82 | postive |
| PJVK | MYG1-AS1 | 0.53740837 | 1.16E-41 | postive |
| GPX4 | AC133552.5 | 0.55337606 | 1.44E-44 | postive |
| PYCARD | LINC01637 | 0.50696377 | 1.54E-36 | postive |
| NLRP1 | AL160006.1 | 0.55166275 | 3.01E-44 | postive |
| NOD1 | AL160006.1 | 0.52652009 | 9.03E-40 | postive |
| PLCG1 | AL160006.1 | 0.52693677 | 7.67E-40 | postive |
| IL18 | LINC01230 | 0.56271248 | 2.44E-46 | postive |
| GSDMB | LINC00861 | 0.62623762 | 4.83E-60 | postive |
| NLRP1 | LINC00861 | 0.59557467 | 4.78E-53 | postive |
| NOD2 | LINC00861 | 0.51671963 | 4.00E-38 | postive |
| PJVK | AC145423.1 | 0.56611658 | 5.33E-47 | postive |
| SCAF11 | AC010186.3 | 0.55054305 | 4.85E-44 | postive |
| GSDMB | AL022328.1 | 0.5942551 | 9.21E-53 | postive |
| NLRP1 | AL022328.1 | 0.59435566 | 8.76E-53 | postive |
| PLCG1 | AL022328.1 | 0.51542958 | 6.52E-38 | postive |
| GSDMB | LINC00342 | 0.81030065 | 9.87E-127 | postive |
| NLRP1 | LINC00342 | 0.65935984 | 1.56E-68 | postive |
| PJVK | LINC00342 | 0.67079643 | 1.01E-71 | postive |
| PLCG1 | LINC00342 | 0.54208139 | 1.70E-42 | postive |
| GSDMB | LINC02062 | 0.57611435 | 5.54E-49 | postive |
| PJVK | LINC02062 | 0.56099354 | 5.22E-46 | postive |
| GSDMB | HOTAIRM1 | 0.65174394 | 1.73E-66 | postive |
| PJVK | HOTAIRM1 | 0.61443887 | 2.93E-57 | postive |
| GSDMB | AL035071.1 | 0.51300625 | 1.63E-37 | postive |
| PJVK | AL035071.1 | 0.52129963 | 6.91E-39 | postive |
| SCAF11 | AP000350.6 | 0.52394195 | 2.48E-39 | postive |
| GSDMB | AP003419.3 | 0.55659037 | 3.59E-45 | postive |
| PJVK | AP003419.3 | 0.63406624 | 5.89E-62 | postive |
| SCAF11 | AC011477.1 | 0.53688457 | 1.43E-41 | postive |
| GSDMB | AL512770.1 | 0.72014918 | 2.68E-87 | postive |
| NLRP1 | AL512770.1 | 0.6081848 | 7.85E-56 | postive |
| PJVK | AL512770.1 | 0.50800401 | 1.05E-36 | postive |
| GSDMB | AC131009.3 | 0.51660492 | 4.18E-38 | postive |
| GSDMB | AC005253.1 | 0.69814434 | 5.84E-80 | postive |
| NLRP1 | AC005253.1 | 0.61018392 | 2.77E-56 | postive |
| NOD1 | AC005253.1 | 0.52629565 | 9.87E-40 | postive |
| PJVK | AC005253.1 | 0.50612156 | 2.10E-36 | postive |
| PLCG1 | AC005253.1 | 0.5509426 | 4.09E-44 | postive |
| GSDMB | AC232271.1 | 0.76186235 | 2.38E-103 | postive |
| NLRP1 | AC232271.1 | 0.55918871 | 1.16E-45 | postive |
| PJVK | AC232271.1 | 0.61129825 | 1.54E-56 | postive |
| GSDMB | NFYC-AS1 | 0.57457566 | 1.13E-48 | postive |
| GSDMB | AC010973.1 | 0.55056984 | 4.80E-44 | postive |
| GSDMB | AC025034.1 | 0.50521906 | 2.93E-36 | postive |
| PJVK | AC025034.1 | 0.50943943 | 6.17E-37 | postive |
| AIM2 | LINC02528 | 0.614458 | 2.90E-57 | postive |
| CASP1 | LINC02528 | 0.65831916 | 2.99E-68 | postive |
| CASP4 | LINC02528 | 0.56566589 | 6.53E-47 | postive |
| CASP5 | LINC02528 | 0.59675703 | 2.65E-53 | postive |
| NLRC4 | LINC02528 | 0.54636761 | 2.84E-43 | postive |
| NOD2 | LINC02528 | 0.74161784 | 3.58E-95 | postive |
| GSDMB | AC116914.2 | 0.6846154 | 9.01E-76 | postive |
| NLRP1 | AC116914.2 | 0.5062344 | 2.02E-36 | postive |
| PJVK | AC116914.2 | 0.52923995 | 3.09E-40 | postive |
| GSDMB | GUSBP11 | 0.62415902 | 1.52E-59 | postive |
| NLRP1 | GUSBP11 | 0.52709346 | 7.21E-40 | postive |
| PJVK | GUSBP11 | 0.52119058 | 7.20E-39 | postive |
| PYCARD | MUC12-AS1 | 0.50192271 | 9.72E-36 | postive |
| GSDMB | AC011462.4 | 0.78669732 | 1.42E-114 | postive |
| NLRP1 | AC011462.4 | 0.53410632 | 4.41E-41 | postive |
| PJVK | AC011462.4 | 0.62844704 | 1.41E-60 | postive |
| GSDMB | AL590666.1 | 0.65412102 | 4.04E-67 | postive |
| NLRP1 | AL590666.1 | 0.63018776 | 5.31E-61 | postive |
| NOD1 | AL157392.3 | 0.56168337 | 3.85E-46 | postive |
| TIRAP | AL731577.2 | 0.51427126 | 1.01E-37 | postive |
| GSDMB | AC024075.2 | 0.59484906 | 6.86E-53 | postive |
| PJVK | AC024075.2 | 0.59096029 | 4.66E-52 | postive |
| GSDMB | AC091185.1 | 0.55501536 | 7.11E-45 | postive |
| NLRP1 | AC091185.1 | 0.50313193 | 6.27E-36 | postive |
| NOD1 | AC091185.1 | 0.51497739 | 7.74E-38 | postive |
| GSDMB | AL442125.1 | 0.53398614 | 4.63E-41 | postive |
| GSDMB | AL031282.2 | 0.58724317 | 2.84E-51 | postive |
| NLRP1 | AL031282.2 | 0.56480507 | 9.60E-47 | postive |
| PLCG1 | AL031282.2 | 0.5002432 | 1.78E-35 | postive |
| SCAF11 | NORAD | 0.59504889 | 6.21E-53 | postive |
| GSDMB | AP000238.1 | 0.60550078 | 3.15E-55 | postive |
| PJVK | AP000238.1 | 0.53704464 | 1.34E-41 | postive |
| SCAF11 | AC005034.2 | 0.52092062 | 8.00E-39 | postive |
| GSDMB | AC010883.1 | 0.67889597 | 4.54E-74 | postive |
| PJVK | AC010883.1 | 0.60488602 | 4.32E-55 | postive |
| NOD1 | AL391845.2 | 0.51407746 | 1.09E-37 | postive |
| PLCG1 | AL391845.2 | 0.51353528 | 1.33E-37 | postive |
| GSDMB | LINC01786 | 0.6282788 | 1.55E-60 | postive |
| PJVK | LINC01786 | 0.52394241 | 2.48E-39 | postive |
| GSDMB | PVT1 | 0.61323326 | 5.55E-57 | postive |
| PJVK | PVT1 | 0.55303369 | 1.67E-44 | postive |
| GSDMB | DICER1-AS1 | 0.62126705 | 7.42E-59 | postive |
| PJVK | DICER1-AS1 | 0.62752866 | 2.35E-60 | postive |
| GSDMB | ITGB2-AS1 | 0.67192059 | 4.81E-72 | postive |
| NLRP1 | ITGB2-AS1 | 0.57741804 | 3.02E-49 | postive |
| NOD2 | ITGB2-AS1 | 0.55042435 | 5.10E-44 | postive |
| PJVK | AL121655.1 | 0.52131298 | 6.87E-39 | postive |
| SCAF11 | AC018752.1 | 0.66253722 | 2.09E-69 | postive |
| IL18 | AC243964.3 | 0.53615311 | 1.93E-41 | postive |
| GSDMB | AC073842.2 | 0.6844911 | 9.82E-76 | postive |
| NLRP1 | AC073842.2 | 0.53731311 | 1.20E-41 | postive |
| PJVK | AC073842.2 | 0.51329154 | 1.46E-37 | postive |
| GSDMB | AC005387.1 | 0.72926914 | 1.50E-90 | postive |
| NLRP1 | AC005387.1 | 0.61159668 | 1.32E-56 | postive |
| PJVK | AC005387.1 | 0.54451621 | 6.16E-43 | postive |
| GSDMB | AC067945.2 | 0.63021424 | 5.23E-61 | postive |
| NLRP1 | AC067945.2 | 0.58999252 | 7.47E-52 | postive |
| SCAF11 | SEMA6A-AS1 | 0.50724786 | 1.39E-36 | postive |
| GSDMB | AL035252.3 | 0.51573869 | 5.80E-38 | postive |
| GSDMB | AC069281.2 | 0.75982833 | 1.72E-102 | postive |
| NLRP1 | AC069281.2 | 0.64028365 | 1.62E-63 | postive |
| PJVK | AC069281.2 | 0.51176603 | 2.59E-37 | postive |
| GSDMB | AC093752.2 | 0.56803641 | 2.25E-47 | postive |
| NLRP1 | AC093752.2 | 0.53175678 | 1.13E-40 | postive |
| IL18 | AC019197.1 | 0.60217369 | 1.73E-54 | postive |
| IL18 | BAALC-AS2 | 0.6156599 | 1.53E-57 | postive |
| GSDMB | AC092118.2 | 0.6575196 | 4.92E-68 | postive |
| NLRP1 | AC092118.2 | 0.55127459 | 3.55E-44 | postive |
| PJVK | AC092118.2 | 0.51063568 | 3.95E-37 | postive |
| GSDMB | AP001458.1 | 0.55382574 | 1.19E-44 | postive |
| NLRP1 | AP001458.1 | 0.57451509 | 1.16E-48 | postive |
| NOD1 | AP001458.1 | 0.51075658 | 3.78E-37 | postive |
| GSDMB | AC005261.3 | 0.52372986 | 2.69E-39 | postive |
| IL18 | SCAMP1-AS1 | 0.50235569 | 8.31E-36 | postive |
| SCAF11 | AC090579.1 | 0.50158938 | 1.10E-35 | postive |
| GSDMB | AC005264.1 | 0.53941248 | 5.09E-42 | postive |
| NLRP1 | AC005264.1 | 0.71364065 | 4.68E-85 | postive |
| NOD1 | AC005264.1 | 0.58061424 | 6.73E-50 | postive |
| PLCG1 | AC005264.1 | 0.61886032 | 2.74E-58 | postive |
| GSDMB | AC108134.3 | 0.63914662 | 3.15E-63 | postive |
| NLRP1 | AC108134.3 | 0.68099786 | 1.09E-74 | postive |
| NOD1 | AC108134.3 | 0.51876451 | 1.83E-38 | postive |
| PLCG1 | AC108134.3 | 0.55416829 | 1.03E-44 | postive |
| PYCARD | RFX5-AS1 | 0.51656517 | 4.24E-38 | postive |
| GSDMB | AC084125.2 | 0.54779938 | 1.55E-43 | postive |
| CASP1 | AC243960.1 | 0.54213987 | 1.65E-42 | postive |
| CASP4 | AC243960.1 | 0.53329809 | 6.10E-41 | postive |
| GSDMB | AC243960.1 | 0.60421001 | 6.11E-55 | postive |
| NLRP1 | AC243960.1 | 0.52401615 | 2.41E-39 | postive |
| NOD2 | AC243960.1 | 0.61821729 | 3.88E-58 | postive |
| SCAF11 | AC087854.1 | 0.57715391 | 3.41E-49 | postive |
| GSDMB | AP001160.4 | 0.56378108 | 1.52E-46 | postive |
| PJVK | AP001160.4 | 0.51486536 | 8.08E-38 | postive |
| PJVK | SNHG11 | 0.50148052 | 1.14E-35 | postive |
| GSDMB | AC105105.1 | 0.52638809 | 9.51E-40 | postive |
| GSDMB | AL139123.1 | 0.73149613 | 2.30E-91 | postive |
| NLRP1 | AL139123.1 | 0.52697588 | 7.55E-40 | postive |
| PJVK | AL139123.1 | 0.70843141 | 2.64E-83 | postive |
| SCAF11 | SEC62-AS1 | 0.52247249 | 4.39E-39 | postive |
| TIRAP | AC005670.3 | 0.55806122 | 1.89E-45 | postive |
| SCAF11 | AF117829.1 | 0.5252935 | 1.46E-39 | postive |
| GSDMB | AC004076.2 | 0.54523179 | 4.57E-43 | postive |
| GSDMB | AC010998.2 | 0.50894135 | 7.42E-37 | postive |
| PJVK | AC007383.1 | 0.5672156 | 3.25E-47 | postive |
| GSDMB | MMP25-AS1 | 0.78050381 | 1.22E-111 | postive |
| NLRP1 | MMP25-AS1 | 0.60987582 | 3.25E-56 | postive |
| SCAF11 | AC116158.1 | 0.52861121 | 3.96E-40 | postive |
| GSDMB | AL359504.1 | 0.60362191 | 8.26E-55 | postive |
| PJVK | AL359504.1 | 0.61219873 | 9.59E-57 | postive |
| SCAF11 | AC107021.1 | 0.51637872 | 4.55E-38 | postive |
| GSDMB | AP002807.1 | 0.80114478 | 8.03E-122 | postive |
| NLRP1 | AP002807.1 | 0.69432112 | 9.41E-79 | postive |
| PLCG1 | AP002807.1 | 0.5241093 | 2.32E-39 | postive |
| GSDMB | AC103691.1 | 0.65639263 | 9.92E-68 | postive |
| NLRP1 | AC103691.1 | 0.50981199 | 5.37E-37 | postive |
| PJVK | AC103691.1 | 0.51175107 | 2.61E-37 | postive |
| CASP4 | AC145098.1 | 0.50100384 | 1.36E-35 | postive |
| CASP5 | AC145098.1 | 0.54698471 | 2.19E-43 | postive |
| NOD2 | AC145098.1 | 0.56190577 | 3.49E-46 | postive |
| AIM2 | PCED1B-AS1 | 0.58029243 | 7.83E-50 | postive |
| CASP1 | PCED1B-AS1 | 0.67180125 | 5.20E-72 | postive |
| CASP4 | PCED1B-AS1 | 0.65148558 | 2.03E-66 | postive |
| CASP5 | PCED1B-AS1 | 0.543283 | 1.03E-42 | postive |
| NOD2 | PCED1B-AS1 | 0.70329762 | 1.28E-81 | postive |
| PYCARD | PCED1B-AS1 | 0.56615905 | 5.23E-47 | postive |
| PJVK | ODC1-DT | 0.54779035 | 1.56E-43 | postive |
| NLRP6 | LINC02747 | 0.51230575 | 2.12E-37 | postive |
| GSDMB | LIX1L-AS1 | 0.61976024 | 1.68E-58 | postive |
| SCAF11 | AC253536.3 | 0.61509788 | 2.06E-57 | postive |
| IL18 | PRDM16-DT | 0.51126379 | 3.13E-37 | postive |
| GSDMB | AC010359.2 | 0.51234259 | 2.09E-37 | postive |
| PJVK | AC010359.2 | 0.55318805 | 1.56E-44 | postive |
| GSDMB | AC022144.1 | 0.54140547 | 2.24E-42 | postive |
| GSDMB | AL159169.2 | 0.60573707 | 2.79E-55 | postive |
| NLRP1 | AL159169.2 | 0.51835366 | 2.14E-38 | postive |
| PJVK | AL159169.2 | 0.64871048 | 1.09E-65 | postive |
| GSDMB | AC005785.1 | 0.74179454 | 3.06E-95 | postive |
| NLRP1 | AC005785.1 | 0.53651513 | 1.66E-41 | postive |
| PJVK | AC005785.1 | 0.65764157 | 4.56E-68 | postive |
| GSDMB | DLGAP1-AS2 | 0.65344944 | 6.11E-67 | postive |
| GSDMB | CACNA1C-AS2 | 0.56098347 | 5.24E-46 | postive |
| GSDMB | AC027601.1 | 0.80309861 | 7.56E-123 | postive |
| NLRP1 | AC027601.1 | 0.69653835 | 1.89E-79 | postive |
| PJVK | AC027601.1 | 0.57584023 | 6.29E-49 | postive |
| PLCG1 | AC027601.1 | 0.57424098 | 1.32E-48 | postive |
| GSDMB | AC124319.1 | 0.58659388 | 3.88E-51 | postive |
| GSDMB | HLA-F-AS1 | 0.59015711 | 6.90E-52 | postive |
| AIM2 | AL590764.1 | 0.5296223 | 2.65E-40 | postive |
| CASP1 | AL590764.1 | 0.68468442 | 8.58E-76 | postive |
| CASP4 | AL590764.1 | 0.59850377 | 1.11E-53 | postive |
| CASP5 | AL590764.1 | 0.51119041 | 3.21E-37 | postive |
| NLRC4 | AL590764.1 | 0.55300873 | 1.69E-44 | postive |
| NOD2 | AL590764.1 | 0.66799571 | 6.27E-71 | postive |
| PYCARD | AL590764.1 | 0.54759098 | 1.70E-43 | postive |
| PJVK | AC005837.4 | 0.50880964 | 7.80E-37 | postive |
| SCAF11 | AC053513.2 | 0.5961421 | 3.61E-53 | postive |
| GSDMB | AC020558.2 | 0.58551239 | 6.53E-51 | postive |
| NLRP1 | AC020558.2 | 0.50781529 | 1.13E-36 | postive |
| GSDMB | LINC02604 | 0.77227512 | 6.98E-108 | postive |
| NLRP1 | LINC02604 | 0.56197578 | 3.38E-46 | postive |
| PJVK | LINC02604 | 0.61181107 | 1.18E-56 | postive |
| GSDMB | AC106028.3 | 0.60041517 | 4.22E-54 | postive |
| PJVK | AC106028.3 | 0.51952766 | 1.37E-38 | postive |
| GSDMB | AC011461.1 | 0.55635205 | 3.98E-45 | postive |
| PJVK | AC011461.1 | 0.50127985 | 1.23E-35 | postive |
| NOD1 | AC067852.3 | 0.51037288 | 4.36E-37 | postive |
| SCAF11 | AC124312.2 | 0.66716036 | 1.08E-70 | postive |
| GSDMB | AGBL5-IT1 | 0.57106684 | 5.67E-48 | postive |
| PJVK | AGBL5-IT1 | 0.54616165 | 3.09E-43 | postive |
| PJVK | PARD3-AS1 | 0.50057234 | 1.58E-35 | postive |
| GSDMB | Z84485.1 | 0.7142984 | 2.80E-85 | postive |
| PJVK | Z84485.1 | 0.60563707 | 2.93E-55 | postive |
| SCAF11 | AC124312.3 | 0.55665417 | 3.49E-45 | postive |
| IL18 | AL049555.1 | 0.5353943 | 2.62E-41 | postive |
| GSDMB | AL451050.2 | 0.6755283 | 4.38E-73 | postive |
| PJVK | AL451050.2 | 0.57429733 | 1.28E-48 | postive |
| GSDMB | FLJ31104 | 0.56004635 | 7.92E-46 | postive |
| PJVK | FLJ31104 | 0.533048 | 6.75E-41 | postive |
| SCAF11 | AC107068.1 | 0.54489903 | 5.25E-43 | postive |
| SCAF11 | AC008906.1 | 0.54145642 | 2.20E-42 | postive |
| GSDMB | AL391684.1 | 0.62507951 | 9.16E-60 | postive |
| PJVK | AL391684.1 | 0.61540759 | 1.75E-57 | postive |
| GSDMB | AC092123.1 | 0.59198834 | 2.81E-52 | postive |
| PJVK | AC092123.1 | 0.53075792 | 1.69E-40 | postive |
| NOD2 | AC011899.2 | 0.53151123 | 1.25E-40 | postive |
| PYCARD | AC011899.2 | 0.51395898 | 1.14E-37 | postive |
| GSDMB | AC092171.4 | 0.59320059 | 1.55E-52 | postive |
| PJVK | AC092171.4 | 0.64090746 | 1.13E-63 | postive |
| SCAF11 | SDCBP2-AS1 | 0.54444171 | 6.35E-43 | postive |
| SCAF11 | AC004112.1 | 0.70835133 | 2.80E-83 | postive |
| NLRP6 | ADORA2A-AS1 | 0.51439997 | 9.63E-38 | postive |
| IL6 | LINC01679 | 0.63365835 | 7.43E-62 | postive |
| PJVK | LGR4-AS1 | 0.51107497 | 3.36E-37 | postive |
| GSDMB | AL031658.1 | 0.510152 | 4.73E-37 | postive |
| NLRP1 | AL031658.1 | 0.63143395 | 2.63E-61 | postive |
| NOD1 | AL031658.1 | 0.55354244 | 1.34E-44 | postive |
| PLCG1 | AL031658.1 | 0.6276166 | 2.24E-60 | postive |
| GSDMB | AL360181.2 | 0.66364523 | 1.03E-69 | postive |
| NLRP1 | AL360181.2 | 0.58915931 | 1.12E-51 | postive |
| PLCG1 | AL360181.2 | 0.56750947 | 2.85E-47 | postive |
| GSDMB | AL353801.3 | 0.50051261 | 1.62E-35 | postive |
| PJVK | AL353801.3 | 0.53243095 | 8.65E-41 | postive |
| GSDMB | AL136295.7 | 0.69231793 | 3.97E-78 | postive |
| NLRP1 | AL136295.7 | 0.59529676 | 5.49E-53 | postive |
| PJVK | AL136295.7 | 0.62071361 | 1.00E-58 | postive |
| SCAF11 | HCG18 | 0.54901656 | 9.28E-44 | postive |
| AIM2 | LASTR | 0.51133259 | 3.05E-37 | postive |
| GSDMB | AC010245.2 | 0.64073136 | 1.25E-63 | postive |
| NLRP1 | AC010245.2 | 0.5430122 | 1.15E-42 | postive |
| PJVK | AC010245.2 | 0.57733719 | 3.13E-49 | postive |
| GSDMB | DGUOK-AS1 | 0.58912157 | 1.14E-51 | postive |
| PJVK | DGUOK-AS1 | 0.5923758 | 2.33E-52 | postive |
| IL18 | AC091151.1 | 0.54210619 | 1.68E-42 | postive |
| GSDMB | AC080038.1 | 0.51146331 | 2.90E-37 | postive |
| GSDMB | PRANCR | 0.61490826 | 2.28E-57 | postive |
| PJVK | PRANCR | 0.60009156 | 4.97E-54 | postive |
| IL18 | NCKAP5-AS2 | 0.58704078 | 3.13E-51 | postive |
| GSDMB | AC004034.1 | 0.71060895 | 4.94E-84 | postive |
| NLRP1 | AC004034.1 | 0.5296762 | 2.60E-40 | postive |
| PJVK | AC004034.1 | 0.56145907 | 4.25E-46 | postive |
| PJVK | AC107464.2 | 0.55393701 | 1.13E-44 | postive |
| GSDMB | MATN1-AS1 | 0.56220894 | 3.05E-46 | postive |
| PJVK | MATN1-AS1 | 0.59683515 | 2.55E-53 | postive |
| GSDMB | AL358072.1 | 0.58146893 | 4.49E-50 | postive |
| PJVK | AL358072.1 | 0.60784444 | 9.37E-56 | postive |
| GSDMB | IGBP1-AS1 | 0.63518066 | 3.11E-62 | postive |
| NLRP1 | IGBP1-AS1 | 0.52241829 | 4.48E-39 | postive |
| PJVK | IGBP1-AS1 | 0.51357898 | 1.31E-37 | postive |
| NOD1 | AC009486.1 | 0.54937463 | 7.97E-44 | postive |
| TIRAP | AC009486.1 | 0.50500148 | 3.17E-36 | postive |
| GSDMB | AL162274.2 | 0.58818825 | 1.80E-51 | postive |
| NLRP1 | AL162274.2 | 0.52984297 | 2.43E-40 | postive |
| PJVK | AL162274.2 | 0.5345377 | 3.70E-41 | postive |
| PJVK | AC004908.3 | 0.51613942 | 4.98E-38 | postive |
| GSDMB | AC073575.2 | 0.70409656 | 7.05E-82 | postive |
| NLRP1 | AC073575.2 | 0.56195786 | 3.41E-46 | postive |
| PJVK | AC073575.2 | 0.52068165 | 8.77E-39 | postive |
| NLRC4 | AL133371.2 | 0.6776112 | 1.08E-73 | postive |
| NLRP3 | AL133371.2 | 0.59297803 | 1.73E-52 | postive |
| GSDMB | MIR762HG | 0.6261993 | 4.93E-60 | postive |
| PJVK | MIR762HG | 0.63051789 | 4.41E-61 | postive |
| GSDMB | AL359921.1 | 0.62332518 | 2.41E-59 | postive |
| NLRP1 | AL359921.1 | 0.5843002 | 1.17E-50 | postive |
| SCAF11 | AC012181.2 | 0.53476936 | 3.37E-41 | postive |
| GSDMB | AC090527.3 | 0.54163537 | 2.04E-42 | postive |
| PJVK | AC090527.3 | 0.55197517 | 2.63E-44 | postive |
| GSDMB | AC110285.2 | 0.544468 | 6.29E-43 | postive |
| NLRP1 | AC110285.2 | 0.51434244 | 9.84E-38 | postive |
| GSDMB | HCG27 | 0.60546903 | 3.20E-55 | postive |
| NLRP1 | HCG27 | 0.51745516 | 3.02E-38 | postive |
| SCAF11 | NUTM2B-AS1 | 0.53085393 | 1.62E-40 | postive |
| GSDMB | AC010973.2 | 0.72849623 | 2.86E-90 | postive |
| NLRP1 | AC010973.2 | 0.5323091 | 9.08E-41 | postive |
| PJVK | AC010973.2 | 0.61276393 | 7.12E-57 | postive |
| GSDMB | ZNF32-AS2 | 0.68007781 | 2.04E-74 | postive |
| NLRP1 | ZNF32-AS2 | 0.6229709 | 2.92E-59 | postive |
| NOD1 | ZNF32-AS2 | 0.52293008 | 3.67E-39 | postive |
| PJVK | ZNF32-AS2 | 0.53093835 | 1.57E-40 | postive |
| PLCG1 | ZNF32-AS2 | 0.5560457 | 4.55E-45 | postive |
| GSDMB | AC027801.1 | 0.57784807 | 2.47E-49 | postive |
| GSDMB | AC113139.1 | 0.52111503 | 7.42E-39 | postive |
| IL18 | LINC01802 | 0.66513121 | 3.99E-70 | postive |
| SCAF11 | AL050343.2 | 0.54380994 | 8.27E-43 | postive |
| SCAF11 | AC079921.1 | 0.52978434 | 2.49E-40 | postive |
| SCAF11 | AC131971.1 | 0.53609633 | 1.97E-41 | postive |
| GSDMB | AC040977.1 | 0.50165806 | 1.07E-35 | postive |
| GSDMB | UBE2Q1-AS1 | 0.67236979 | 3.58E-72 | postive |
| NLRP1 | UBE2Q1-AS1 | 0.60195264 | 1.94E-54 | postive |
| PLCG1 | UBE2Q1-AS1 | 0.50257863 | 7.66E-36 | postive |
| NOD1 | AC108010.1 | 0.58171312 | 4.00E-50 | postive |
| SCAF11 | AC108010.1 | 0.58149874 | 4.43E-50 | postive |
| TIRAP | AC108010.1 | 0.51540325 | 6.59E-38 | postive |
| GSDMB | AL928654.2 | 0.67083714 | 9.81E-72 | postive |
| NLRP1 | AL928654.2 | 0.62723619 | 2.77E-60 | postive |
| NOD1 | AL928654.2 | 0.50641101 | 1.89E-36 | postive |
| PJVK | AL928654.2 | 0.53840044 | 7.71E-42 | postive |
| PLCG1 | AL928654.2 | 0.58571342 | 5.93E-51 | postive |
| AIM2 | AC090152.1 | 0.55429254 | 9.72E-45 | postive |
| GSDMB | AC073957.3 | 0.53462824 | 3.57E-41 | postive |
| GSDMB | AP000692.1 | 0.59972194 | 5.99E-54 | postive |
| NLRP1 | AP000692.1 | 0.56450692 | 1.10E-46 | postive |
| SCAF11 | RAP2C-AS1 | 0.71985779 | 3.39E-87 | postive |
| TIRAP | RAP2C-AS1 | 0.51263343 | 1.87E-37 | postive |
| PJVK | AC004069.1 | 0.51711055 | 3.45E-38 | postive |
| SCAF11 | TH2LCRR | 0.51168426 | 2.67E-37 | postive |
| SCAF11 | BCL2L1-AS1 | 0.52794812 | 5.15E-40 | postive |
| IL18 | AP000757.1 | 0.5020036 | 9.44E-36 | postive |
| GSDMB | AC005899.6 | 0.651785 | 1.69E-66 | postive |
| NLRP1 | AC005899.6 | 0.52017741 | 1.06E-38 | postive |
| PJVK | AC005899.6 | 0.55992509 | 8.36E-46 | postive |
| SCAF11 | AC025917.1 | 0.53386319 | 4.86E-41 | postive |
| GSDMB | AC012615.1 | 0.66599089 | 2.29E-70 | postive |
| NLRP1 | AC012615.1 | 0.56399276 | 1.38E-46 | postive |
| PJVK | AC012615.1 | 0.65301403 | 7.98E-67 | postive |
| GSDMB | SNHG20 | 0.67271103 | 2.85E-72 | postive |
| NLRP1 | SNHG20 | 0.62355354 | 2.12E-59 | postive |
| NOD1 | SNHG20 | 0.50740143 | 1.31E-36 | postive |
| PJVK | SNHG20 | 0.50969863 | 5.60E-37 | postive |
| PLCG1 | SNHG20 | 0.59496862 | 6.46E-53 | postive |
| GSDMB | LINC01410 | 0.53961704 | 4.68E-42 | postive |
| GSDMB | ERVK13-1 | 0.59509952 | 6.06E-53 | postive |
| PJVK | ERVK13-1 | 0.54945538 | 7.70E-44 | postive |
| GSDMB | AL021707.1 | 0.63683628 | 1.20E-62 | postive |
| NLRP1 | AL021707.1 | 0.53421218 | 4.22E-41 | postive |
| PJVK | AL021707.1 | 0.52483841 | 1.75E-39 | postive |
| GSDMB | ADIRF-AS1 | 0.50950128 | 6.03E-37 | postive |
| SCAF11 | ADAMTS9-AS2 | 0.54204065 | 1.72E-42 | postive |
| PJVK | AC026471.2 | 0.59430589 | 8.98E-53 | postive |
| GSDMB | ZNRD2-AS1 | 0.57446828 | 1.19E-48 | postive |
| NLRP1 | ZNRD2-AS1 | 0.50740383 | 1.31E-36 | postive |
| GSDMB | AC090589.3 | 0.69708738 | 1.26E-79 | postive |
| NLRP1 | AC090589.3 | 0.61191313 | 1.12E-56 | postive |
| PJVK | AC090589.3 | 0.51551696 | 6.31E-38 | postive |
| PLCG1 | AC090589.3 | 0.50484665 | 3.35E-36 | postive |
| NOD1 | AC108463.2 | 0.55793331 | 2.00E-45 | postive |
| IL18 | INSYN1-AS1 | 0.50698136 | 1.53E-36 | postive |
| GSDMB | AC022211.3 | 0.59125475 | 4.03E-52 | postive |
| GSDMB | ASB16-AS1 | 0.71959156 | 4.19E-87 | postive |
| NLRP1 | ASB16-AS1 | 0.53416068 | 4.31E-41 | postive |
| PJVK | ASB16-AS1 | 0.57228072 | 3.25E-48 | postive |
| GSDMB | ZNF436-AS1 | 0.63776177 | 7.03E-63 | postive |
| NLRP1 | ZNF436-AS1 | 0.51696794 | 3.64E-38 | postive |
| PJVK | ZNF436-AS1 | 0.61765303 | 5.25E-58 | postive |
| GSDMB | NFE2L1-DT | 0.66053489 | 7.43E-69 | postive |
| PJVK | NFE2L1-DT | 0.67532647 | 5.02E-73 | postive |
| GSDMB | AC040162.3 | 0.78028958 | 1.54E-111 | postive |
| NLRP1 | AC040162.3 | 0.62697294 | 3.21E-60 | postive |
| PJVK | AC040162.3 | 0.56700563 | 3.58E-47 | postive |
| PLCG1 | AC040162.3 | 0.52047894 | 9.48E-39 | postive |
| GSDMB | AC073389.3 | 0.64646197 | 4.21E-65 | postive |
| PJVK | AC073389.3 | 0.63143213 | 2.63E-61 | postive |
| GPX4 | PRR34-AS1 | 0.6285433 | 1.34E-60 | postive |
| SCAF11 | AL133227.1 | 0.50960903 | 5.79E-37 | postive |
| GSDMB | SCAT2 | 0.70831219 | 2.89E-83 | postive |
| PJVK | SCAT2 | 0.71359517 | 4.85E-85 | postive |
| GPX4 | GIHCG | 0.50579605 | 2.37E-36 | postive |
| GSDMB | AC006064.1 | 0.562918 | 2.23E-46 | postive |
| NLRP1 | AC006064.1 | 0.50284418 | 6.96E-36 | postive |
| GSDMB | HOXB-AS1 | 0.65161668 | 1.87E-66 | postive |
| PJVK | HOXB-AS1 | 0.6591117 | 1.82E-68 | postive |
| SCAF11 | AC024075.3 | 0.54778535 | 1.56E-43 | postive |
| GSDMB | AL138921.1 | 0.65663275 | 8.55E-68 | postive |
| NLRP1 | AL138921.1 | 0.5502233 | 5.56E-44 | postive |
| PJVK | AL138921.1 | 0.50788056 | 1.10E-36 | postive |
| IL18 | AC007342.5 | 0.55278 | 1.86E-44 | postive |
| GSDMB | AC016957.2 | 0.62884867 | 1.13E-60 | postive |
| NLRP1 | AC016957.2 | 0.56362814 | 1.62E-46 | postive |
| PJVK | AC016957.2 | 0.54176928 | 1.93E-42 | postive |
| GSDMB | AC087289.5 | 0.64697065 | 3.11E-65 | postive |
| PJVK | AC087289.5 | 0.54020688 | 3.68E-42 | postive |
| GSDMB | AF111169.3 | 0.51593206 | 5.39E-38 | postive |
| NLRP1 | AF111169.3 | 0.64206342 | 5.72E-64 | postive |
| NOD1 | AF111169.3 | 0.59539729 | 5.22E-53 | postive |
| PLCG1 | AF111169.3 | 0.52971354 | 2.56E-40 | postive |
| GSDMB | AC012409.3 | 0.50425275 | 4.17E-36 | postive |
| NLRP1 | AC012409.3 | 0.51614115 | 4.98E-38 | postive |
| GSDMB | AC013731.1 | 0.66746724 | 8.84E-71 | postive |
| PJVK | AC013731.1 | 0.68201482 | 5.41E-75 | postive |
| GSDMB | AL590096.1 | 0.59638108 | 3.20E-53 | postive |
| PJVK | AL590096.1 | 0.52405204 | 2.37E-39 | postive |
| GSDMB | MANEA-DT | 0.50205092 | 9.28E-36 | postive |
| GSDMB | AP000892.3 | 0.58295881 | 2.21E-50 | postive |
| PJVK | AP000892.3 | 0.60001375 | 5.17E-54 | postive |
| GSDMB | AL022328.3 | 0.75567546 | 9.13E-101 | postive |
| NLRP1 | AL022328.3 | 0.72476458 | 6.29E-89 | postive |
| PJVK | AL022328.3 | 0.52395171 | 2.47E-39 | postive |
| PLCG1 | AL022328.3 | 0.52294804 | 3.65E-39 | postive |
| GSDMB | AC105345.1 | 0.51812403 | 2.34E-38 | postive |
| NLRP1 | AC105345.1 | 0.56428907 | 1.21E-46 | postive |
| GSDMB | AC009065.8 | 0.55500685 | 7.14E-45 | postive |
| PJVK | AC009065.8 | 0.52926712 | 3.05E-40 | postive |
| GSDMB | AL121987.2 | 0.51045522 | 4.23E-37 | postive |
| NLRP1 | AL121987.2 | 0.61387783 | 3.95E-57 | postive |
| NOD1 | AL121987.2 | 0.56875277 | 1.62E-47 | postive |
| PLCG1 | AL121987.2 | 0.53651225 | 1.66E-41 | postive |
| GSDMB | AL359921.2 | 0.59560612 | 4.71E-53 | postive |
| PJVK | AL359921.2 | 0.53093434 | 1.57E-40 | postive |
| SCAF11 | AC004492.1 | 0.61775636 | 4.97E-58 | postive |
| GSDMD | AC008608.2 | 0.51399298 | 1.12E-37 | postive |
| GSDMB | LINC00528 | 0.69934489 | 2.42E-80 | postive |
| NLRP1 | LINC00528 | 0.69417464 | 1.05E-78 | postive |
| GSDMB | AL117336.2 | 0.58987257 | 7.92E-52 | postive |
| GSDMB | PRKCZ-AS1 | 0.5418119 | 1.90E-42 | postive |
| NLRP1 | PRKCZ-AS1 | 0.56112931 | 4.92E-46 | postive |
| PLCG1 | PRKCZ-AS1 | 0.50876658 | 7.92E-37 | postive |
| IL18 | AC010501.2 | 0.63768628 | 7.35E-63 | postive |
| GSDMB | NDUFV2-AS1 | 0.56053491 | 6.39E-46 | postive |
| PJVK | NDUFV2-AS1 | 0.54978077 | 6.71E-44 | postive |
| GSDMB | AC004846.2 | 0.59889537 | 9.09E-54 | postive |
| PJVK | AC004846.2 | 0.5226393 | 4.11E-39 | postive |
| GSDMB | LINC00921 | 0.53643309 | 1.72E-41 | postive |
| NLRP1 | LINC00921 | 0.61591183 | 1.34E-57 | postive |
| NOD1 | LINC00921 | 0.57231022 | 3.21E-48 | postive |
| PLCG1 | LINC00921 | 0.5395355 | 4.84E-42 | postive |
| GSDMB | AC100778.2 | 0.51942488 | 1.42E-38 | postive |
| GSDMB | AL354760.1 | 0.67636838 | 2.50E-73 | postive |
| PJVK | AL354760.1 | 0.63160664 | 2.38E-61 | postive |
| GSDMB | LINC00106 | 0.63464275 | 4.23E-62 | postive |
| NLRP1 | LINC00106 | 0.50088193 | 1.42E-35 | postive |
| PJVK | LINC00106 | 0.50284371 | 6.96E-36 | postive |
| GSDMB | SNHG17 | 0.55700421 | 3.00E-45 | postive |
| PJVK | SNHG17 | 0.50183603 | 1.00E-35 | postive |
| GSDMB | AC003070.1 | 0.6986523 | 4.02E-80 | postive |
| NLRP1 | AC003070.1 | 0.69253353 | 3.40E-78 | postive |
| PJVK | AC003070.1 | 0.52292575 | 3.68E-39 | postive |
| PLCG1 | AC003070.1 | 0.54960659 | 7.22E-44 | postive |
| GSDMB | AL023653.1 | 0.69035927 | 1.60E-77 | postive |
| NLRP1 | AL023653.1 | 0.53738988 | 1.16E-41 | postive |
| NOD2 | AL023653.1 | 0.51244583 | 2.01E-37 | postive |
| SCAF11 | AC012313.5 | 0.50794433 | 1.07E-36 | postive |
| GSDMB | AL122125.1 | 0.50568296 | 2.47E-36 | postive |
| GSDMB | AC008760.1 | 0.61575617 | 1.45E-57 | postive |
| NLRP1 | AC008760.1 | 0.53550197 | 2.51E-41 | postive |
| GSDMB | AL137127.1 | 0.50933527 | 6.41E-37 | postive |
| GSDMB | MELTF-AS1 | 0.58365598 | 1.59E-50 | postive |
| PJVK | VPS13B-DT | 0.5649294 | 9.08E-47 | postive |
| GSDMB | CSGALNACT2-DT | 0.67028705 | 1.41E-71 | postive |
| NLRP1 | CSGALNACT2-DT | 0.51648608 | 4.37E-38 | postive |
| PJVK | CSGALNACT2-DT | 0.60233345 | 1.59E-54 | postive |
| GSDMB | AC002059.1 | 0.61565559 | 1.53E-57 | postive |
| NLRP1 | AC002059.1 | 0.53688095 | 1.43E-41 | postive |
| PJVK | AC002059.1 | 0.51564177 | 6.02E-38 | postive |
| GSDMB | LINC00893 | 0.73391652 | 2.93E-92 | postive |
| NLRP1 | LINC00893 | 0.58776898 | 2.20E-51 | postive |
| PJVK | LINC00893 | 0.57745602 | 2.96E-49 | postive |
| GSDMB | AC005332.7 | 0.50731724 | 1.35E-36 | postive |
| GSDMB | AC090510.2 | 0.58182726 | 3.79E-50 | postive |
| NLRP1 | AC090510.2 | 0.50432703 | 4.06E-36 | postive |
| PJVK | AC090510.2 | 0.52279286 | 3.87E-39 | postive |
| NLRP1 | AL356481.1 | 0.50160703 | 1.09E-35 | postive |
| SCAF11 | AL590723.1 | 0.52356259 | 2.87E-39 | postive |
| SCAF11 | AC090181.2 | 0.52731789 | 6.60E-40 | postive |
| GSDMB | AC137932.3 | 0.61477801 | 2.45E-57 | postive |
| GSDMB | PTOV1-AS1 | 0.65510747 | 2.20E-67 | postive |
| NLRP1 | PTOV1-AS1 | 0.54761373 | 1.68E-43 | postive |
| PJVK | PTOV1-AS1 | 0.5584639 | 1.59E-45 | postive |
| SCAF11 | LINC00571 | 0.55516947 | 6.65E-45 | postive |
| PJVK | AC006449.6 | 0.57197642 | 3.74E-48 | postive |
| GSDMB | AC087289.2 | 0.77284976 | 3.86E-108 | postive |
| NLRP1 | AC087289.2 | 0.65161176 | 1.88E-66 | postive |
| PJVK | AC087289.2 | 0.65464353 | 2.93E-67 | postive |
| GSDMB | AL662797.1 | 0.73114602 | 3.09E-91 | postive |
| NLRP1 | AL662797.1 | 0.56527989 | 7.76E-47 | postive |
| GSDMB | AC005776.2 | 0.59279369 | 1.89E-52 | postive |
| GSDMB | H1-10-AS1 | 0.73309656 | 5.90E-92 | postive |
| NLRP1 | H1-10-AS1 | 0.69086583 | 1.12E-77 | postive |
| PJVK | H1-10-AS1 | 0.52246859 | 4.39E-39 | postive |
| PLCG1 | H1-10-AS1 | 0.59298974 | 1.72E-52 | postive |
| GSDMB | AC004918.1 | 0.59075285 | 5.16E-52 | postive |
| NLRP1 | AC004918.1 | 0.57234831 | 3.15E-48 | postive |
| PJVK | AC004918.1 | 0.53646197 | 1.70E-41 | postive |
| GSDMB | AC008764.8 | 0.7978446 | 4.09E-120 | postive |
| NLRP1 | AC008764.8 | 0.58859784 | 1.47E-51 | postive |
| PJVK | AC008764.8 | 0.59540127 | 5.21E-53 | postive |
| GSDMB | ZEB2-AS1 | 0.5661148 | 5.34E-47 | postive |
| NLRP1 | ZEB2-AS1 | 0.50904714 | 7.14E-37 | postive |
| PJVK | ZEB2-AS1 | 0.57125639 | 5.20E-48 | postive |
| GSDMB | AC127024.4 | 0.53961244 | 4.69E-42 | postive |
| GSDMB | MRPL20-DT | 0.68930524 | 3.38E-77 | postive |
| NLRP1 | MRPL20-DT | 0.53307462 | 6.68E-41 | postive |
| PJVK | MRPL20-DT | 0.57368812 | 1.70E-48 | postive |
| SCAF11 | AC012404.2 | 0.51270406 | 1.82E-37 | postive |
| GSDMB | PTOV1-AS2 | 0.80344429 | 4.96E-123 | postive |
| NLRP1 | PTOV1-AS2 | 0.71281839 | 8.90E-85 | postive |
| PJVK | PTOV1-AS2 | 0.5998985 | 5.48E-54 | postive |
| PLCG1 | PTOV1-AS2 | 0.59118678 | 4.17E-52 | postive |
| GSDMB | AC007038.1 | 0.6554957 | 1.73E-67 | postive |
| PJVK | AC007038.1 | 0.65152783 | 1.98E-66 | postive |
| SCAF11 | AL133243.2 | 0.57499799 | 9.29E-49 | postive |
| SCAF11 | AC024075.1 | 0.58019885 | 8.18E-50 | postive |
| TIRAP | AC024075.1 | 0.56850004 | 1.82E-47 | postive |
| IL6 | AC004264.1 | 0.5055778 | 2.57E-36 | postive |
| PJVK | AC021851.1 | 0.52256994 | 4.22E-39 | postive |
| GSDMB | DM1-AS | 0.64567125 | 6.75E-65 | postive |
| PJVK | DM1-AS | 0.50657494 | 1.78E-36 | postive |
| NLRP6 | AC007406.2 | 0.55639585 | 3.91E-45 | postive |
| SCAF11 | AP000766.1 | 0.63975116 | 2.21E-63 | postive |
| GSDMB | CHROMR | 0.51112326 | 3.30E-37 | postive |
| PJVK | CHROMR | 0.5030203 | 6.53E-36 | postive |
| PLCG1 | CHROMR | 0.51423966 | 1.02E-37 | postive |
| GSDMB | AC099778.1 | 0.55853337 | 1.54E-45 | postive |
| PJVK | AC099778.1 | 0.54430716 | 6.72E-43 | postive |
| GSDMB | CAHM | 0.55516289 | 6.67E-45 | postive |
| PJVK | CAHM | 0.61426204 | 3.22E-57 | postive |
| GSDMB | AC008610.1 | 0.6681335 | 5.74E-71 | postive |
| PJVK | AC008610.1 | 0.60890619 | 5.39E-56 | postive |
| GSDMB | AC104758.1 | 0.64687378 | 3.29E-65 | postive |
| NLRP1 | AC104758.1 | 0.53019068 | 2.12E-40 | postive |
| IL18 | AC090515.2 | 0.55716555 | 2.80E-45 | postive |
| GSDMB | FSIP2-AS1 | 0.54515374 | 4.72E-43 | postive |
| PJVK | FSIP2-AS1 | 0.56458732 | 1.06E-46 | postive |
| SCAF11 | LINC00472 | 0.52113323 | 7.37E-39 | postive |
| GSDMB | AC017104.1 | 0.63473268 | 4.02E-62 | postive |
| GSDMB | PSMA3-AS1 | 0.62154294 | 6.39E-59 | postive |
| NLRP1 | PSMA3-AS1 | 0.6086022 | 6.32E-56 | postive |
| NOD1 | PSMA3-AS1 | 0.52067207 | 8.80E-39 | postive |
| PJVK | PSMA3-AS1 | 0.52305758 | 3.50E-39 | postive |
| PLCG1 | PSMA3-AS1 | 0.50849331 | 8.76E-37 | postive |
