# Supplementary material for: A Novel Pyroptosis-Related lncRNAs Signature for Predicting the Prognosis of Kidney Renal Clear Cell Carcinoma and Its Associations with Immunity
Source: J Oncol. 2021 Oct 18;2021:9997185. doi: 10.1155/2021/9997185 (PMC8577956; doi:10.1155/2021/9997185)
Supplement: Supplementary Materials — Supplementary File Table S1. Patients' clinical features from the TCGA dataset. Supplementary File Table S2. 33 pyroptosis-related genes. Supplementary File Table S3. The sequences of primers and siRNAs used in this study. Supplementary File Table S4. 14 pyroptosis-related DEGs from TCGA-KIRC. Supplementary File Table S5. 1042 pyroptosis-related lncRNAs. Supplementary File Table S6. 299 significant pyroptosis-related lncRNAs after univariate Cox analysis. Supplementary File Figure S1. 14 pyroptosis-related DEGs from TCGA-KIRC. [file 9997185.f1.zip › 9997185.f1/Table S6 (1).docx]

**Table S6:** 299 significant pyroptosis-related lncRNAs after univariate Cox analysis

| gene | HR | HR.95L | HR.95H | pvalue |
| --- | --- | --- | --- | --- |
| AC008875.1 | 1.765798 | 1.468322 | 2.123541 | 1.53E-09 |
| AC002553.1 | 1.319352 | 1.165523 | 1.493483 | 1.18E-05 |
| AL355488.1 | 1.280497 | 1.184385 | 1.384408 | 5.27E-10 |
| LINC01389 | 1.785388 | 1.427547 | 2.232929 | 3.79E-07 |
| AC132192.2 | 1.525003 | 1.338622 | 1.737336 | 2.23E-10 |
| AC005393.1 | 1.870422 | 1.49532 | 2.339619 | 4.18E-08 |
| AL158834.2 | 3.977824 | 2.475168 | 6.392734 | 1.17E-08 |
| LINC00926 | 1.669894 | 1.400839 | 1.990626 | 1.06E-08 |
| AC109460.1 | 1.768882 | 1.303012 | 2.401315 | 0.000255 |
| AC245884.8 | 1.170086 | 1.090086 | 1.255957 | 1.38E-05 |
| AC011472.1 | 1.106703 | 1.042459 | 1.174906 | 0.000891 |
| AL022322.1 | 1.176639 | 1.078592 | 1.2836 | 0.000248 |
| AL353622.1 | 1.145379 | 1.058768 | 1.239075 | 0.000716 |
| AC092809.4 | 2.280811 | 1.679774 | 3.096904 | 1.27E-07 |
| FOXO6-AS1 | 1.857399 | 1.356102 | 2.544006 | 0.000114 |
| AC009133.1 | 1.167778 | 1.076649 | 1.266621 | 0.000183 |
| AC007220.1 | 2.233707 | 1.568105 | 3.181833 | 8.50E-06 |
| AC010168.2 | 1.381896 | 1.165057 | 1.639094 | 0.000204 |
| LINC00174 | 1.239072 | 1.14109 | 1.345467 | 3.39E-07 |
| AC006272.1 | 3.062506 | 1.949752 | 4.810325 | 1.18E-06 |
| AL049840.5 | 1.06466 | 1.025874 | 1.104913 | 0.000936 |
| GARS1-DT | 1.29581 | 1.145936 | 1.465286 | 3.59E-05 |
| NARF-IT1 | 2.167216 | 1.581752 | 2.96938 | 1.48E-06 |
| AC127502.2 | 1.219121 | 1.143088 | 1.300211 | 1.64E-09 |
| AL158151.4 | 1.827022 | 1.520694 | 2.195057 | 1.22E-10 |
| AC009690.2 | 2.175448 | 1.547547 | 3.058111 | 7.71E-06 |
| AC004253.1 | 1.49502 | 1.255262 | 1.780572 | 6.51E-06 |
| AL606534.1 | 4.614154 | 2.374712 | 8.965473 | 6.43E-06 |
| AC135050.3 | 1.128424 | 1.068356 | 1.19187 | 1.50E-05 |
| RNF139-AS1 | 5.793775 | 3.398155 | 9.878251 | 1.09E-10 |
| AC048341.2 | 1.101621 | 1.066367 | 1.13804 | 5.47E-09 |
| FAM13A-AS1 | 1.299662 | 1.143413 | 1.477263 | 6.05E-05 |
| SNHG4 | 1.94177 | 1.560275 | 2.416542 | 2.75E-09 |
| AC010201.2 | 1.304143 | 1.134716 | 1.498866 | 0.000184 |
| AP003352.1 | 1.333013 | 1.202948 | 1.477139 | 4.08E-08 |
| AC126118.1 | 1.547045 | 1.215245 | 1.969438 | 0.000396 |
| LMNTD2-AS1 | 1.052971 | 1.031458 | 1.074934 | 9.55E-07 |
| OGFR-AS1 | 2.560248 | 1.774622 | 3.693671 | 4.98E-07 |
| MRPS9-AS1 | 2.641779 | 1.773151 | 3.93593 | 1.79E-06 |
| AC138207.4 | 1.296963 | 1.122479 | 1.49857 | 0.00042 |
| AC087623.1 | 1.278413 | 1.124897 | 1.452879 | 0.000168 |
| AL513320.1 | 1.321996 | 1.18155 | 1.479136 | 1.11E-06 |
| AL031705.1 | 3.402104 | 1.926887 | 6.00674 | 2.43E-05 |
| LINC00894 | 1.876855 | 1.525196 | 2.309594 | 2.72E-09 |
| AC100803.4 | 1.453635 | 1.266924 | 1.667862 | 9.66E-08 |
| LINC01871 | 1.166789 | 1.097403 | 1.240562 | 8.17E-07 |
| LINC01004 | 1.25852 | 1.168674 | 1.355274 | 1.17E-09 |
| AP001767.2 | 1.760927 | 1.380048 | 2.246925 | 5.36E-06 |
| KDM4A-AS1 | 1.821919 | 1.416939 | 2.342647 | 2.91E-06 |
| AC022167.2 | 2.021467 | 1.459126 | 2.800532 | 2.32E-05 |
| AL117379.1 | 1.29978 | 1.194653 | 1.414157 | 1.11E-09 |
| AP001160.1 | 1.532478 | 1.322431 | 1.775889 | 1.38E-08 |
| AL442128.2 | 4.241044 | 2.358891 | 7.624961 | 1.38E-06 |
| AP000553.2 | 1.568116 | 1.340836 | 1.833922 | 1.79E-08 |
| AL133410.1 | 1.923783 | 1.537395 | 2.40728 | 1.07E-08 |
| AC084876.1 | 1.634488 | 1.413797 | 1.889629 | 3.16E-11 |
| AC004585.1 | 1.24128 | 1.107811 | 1.390829 | 0.000196 |
| AC016737.1 | 2.563801 | 1.893638 | 3.471135 | 1.13E-09 |
| AC055855.1 | 1.77976 | 1.334959 | 2.372765 | 8.53E-05 |
| AL158196.1 | 4.623575 | 2.594948 | 8.238102 | 2.04E-07 |
| AC132872.3 | 1.047692 | 1.022719 | 1.073275 | 0.000154 |
| AL021707.8 | 1.234171 | 1.118019 | 1.362389 | 3.02E-05 |
| AL096701.3 | 2.703995 | 1.844944 | 3.963041 | 3.40E-07 |
| AC011498.6 | 1.508294 | 1.220304 | 1.864249 | 0.000144 |
| U47924.3 | 1.889101 | 1.549591 | 2.302997 | 3.11E-10 |
| AL031600.1 | 1.359503 | 1.167918 | 1.582516 | 7.41E-05 |
| AC022126.1 | 3.533765 | 2.204098 | 5.665582 | 1.59E-07 |
| AC011468.1 | 1.212044 | 1.123491 | 1.307576 | 6.76E-07 |
| AC138932.4 | 1.868043 | 1.459315 | 2.391249 | 7.05E-07 |
| AC091057.1 | 3.769189 | 2.317012 | 6.131513 | 9.06E-08 |
| LAMC1-AS1 | 1.656812 | 1.266639 | 2.167173 | 0.000229 |
| ASMTL-AS1 | 1.055823 | 1.034709 | 1.077369 | 1.36E-07 |
| AC025171.5 | 2.202154 | 1.672033 | 2.900352 | 1.93E-08 |
| AL161452.1 | 2.827972 | 1.764461 | 4.532504 | 1.56E-05 |
| AC244197.2 | 1.378381 | 1.225545 | 1.550276 | 8.71E-08 |
| LINC00941 | 1.444704 | 1.303713 | 1.600943 | 2.19E-12 |
| AL162586.1 | 1.281133 | 1.164625 | 1.409296 | 3.53E-07 |
| ARHGAP27P1-BPTFP1-KPNA2P3 | 1.276064 | 1.137182 | 1.431908 | 3.37E-05 |
| RASGRP3-AS1 | 1.727075 | 1.463768 | 2.037748 | 9.51E-11 |
| YEATS2-AS1 | 2.42503 | 1.822427 | 3.226889 | 1.22E-09 |
| RUSC1-AS1 | 1.332676 | 1.18365 | 1.500465 | 2.07E-06 |
| AC005332.5 | 1.393373 | 1.228189 | 1.580774 | 2.57E-07 |
| AL109659.2 | 2.282566 | 1.407097 | 3.702734 | 0.000827 |
| LINC01311 | 2.217583 | 1.715986 | 2.865803 | 1.15E-09 |
| LINC01943 | 1.630889 | 1.388405 | 1.915721 | 2.59E-09 |
| AL133406.2 | 2.917465 | 1.74184 | 4.886558 | 4.73E-05 |
| AC087741.1 | 1.318472 | 1.188652 | 1.462472 | 1.72E-07 |
| AC026356.2 | 1.423358 | 1.252794 | 1.617143 | 5.94E-08 |
| AL645940.1 | 2.19397 | 1.677057 | 2.87021 | 9.94E-09 |
| MED8-AS1 | 2.056126 | 1.662148 | 2.543489 | 3.10E-11 |
| AL592211.1 | 2.922316 | 1.917679 | 4.453263 | 6.06E-07 |
| AL683807.1 | 1.324359 | 1.168398 | 1.501138 | 1.11E-05 |
| AC105020.1 | 1.180396 | 1.090881 | 1.277258 | 3.76E-05 |
| KIF1C-AS1 | 2.132706 | 1.685034 | 2.699314 | 2.96E-10 |
| AC084824.5 | 1.37062 | 1.242008 | 1.512551 | 3.59E-10 |
| AC010201.1 | 1.691683 | 1.40624 | 2.035065 | 2.47E-08 |
| AC092301.1 | 3.901864 | 2.135853 | 7.128087 | 9.50E-06 |
| AC073655.2 | 1.661732 | 1.36896 | 2.017117 | 2.81E-07 |
| AC012645.3 | 1.57871 | 1.225832 | 2.03317 | 0.000404 |
| AC116407.2 | 1.330684 | 1.202887 | 1.472058 | 2.93E-08 |
| AC005104.1 | 1.362134 | 1.199414 | 1.54693 | 1.92E-06 |
| SNHG12 | 1.041764 | 1.018796 | 1.065249 | 0.000322 |
| NALT1 | 1.508514 | 1.322934 | 1.720128 | 8.34E-10 |
| SLBP-DT | 1.760666 | 1.536533 | 2.017493 | 3.87E-16 |
| AC233728.1 | 1.892019 | 1.465371 | 2.442886 | 1.00E-06 |
| AL590560.3 | 1.095026 | 1.05361 | 1.13807 | 3.94E-06 |
| STAG3L5P-PVRIG2P-PILRB | 1.234139 | 1.103661 | 1.380042 | 0.000224 |
| AC022973.5 | 3.975265 | 2.180509 | 7.247268 | 6.66E-06 |
| LINC01801 | 0.443644 | 0.304682 | 0.645987 | 2.24E-05 |
| LINC01160 | 3.994826 | 2.35514 | 6.776086 | 2.79E-07 |
| AC004148.1 | 1.262737 | 1.159911 | 1.374679 | 7.33E-08 |
| LINC00115 | 1.669524 | 1.377249 | 2.023825 | 1.79E-07 |
| AC245052.4 | 2.765473 | 1.601071 | 4.776703 | 0.000264 |
| ANKRD10-IT1 | 1.044564 | 1.018193 | 1.071618 | 0.000832 |
| AC005840.2 | 1.370961 | 1.196681 | 1.570623 | 5.41E-06 |
| AC092119.2 | 1.864768 | 1.54595 | 2.249336 | 7.33E-11 |
| ZKSCAN2-DT | 1.634185 | 1.382826 | 1.931233 | 8.23E-09 |
| AC009120.2 | 1.286108 | 1.17071 | 1.412881 | 1.56E-07 |
| ADAMTSL4-AS2 | 1.265012 | 1.134034 | 1.411117 | 2.49E-05 |
| AC084018.1 | 1.13975 | 1.07774 | 1.205328 | 4.59E-06 |
| AC015961.2 | 1.600657 | 1.291238 | 1.984223 | 1.77E-05 |
| AL021707.6 | 1.122412 | 1.065666 | 1.18218 | 1.28E-05 |
| AC025265.1 | 1.125058 | 1.080676 | 1.171263 | 9.57E-09 |
| NCBP2-AS1 | 3.12053 | 1.852684 | 5.255998 | 1.89E-05 |
| AC008750.1 | 1.913163 | 1.428262 | 2.56269 | 1.36E-05 |
| AC127024.6 | 2.94689 | 2.037516 | 4.262132 | 9.46E-09 |
| AC024361.3 | 1.845361 | 1.320421 | 2.578992 | 0.000334 |
| LINC00426 | 1.659722 | 1.245244 | 2.212159 | 0.000548 |
| AC006435.2 | 1.369972 | 1.197525 | 1.567252 | 4.52E-06 |
| AL512652.1 | 4.094889 | 2.615081 | 6.412084 | 7.21E-10 |
| AC025171.4 | 1.150611 | 1.073209 | 1.233595 | 7.87E-05 |
| AC007743.1 | 0.573697 | 0.439315 | 0.749185 | 4.49E-05 |
| LINC01355 | 1.446259 | 1.295215 | 1.614918 | 5.51E-11 |
| AL135999.1 | 1.466411 | 1.258653 | 1.708461 | 9.05E-07 |
| AC010719.1 | 1.3513 | 1.202846 | 1.518074 | 3.97E-07 |
| LINC02019 | 2.703648 | 1.840051 | 3.972561 | 4.07E-07 |
| AC021078.1 | 1.127146 | 1.055648 | 1.203486 | 0.000344 |
| AC087239.1 | 1.557316 | 1.297014 | 1.869858 | 2.07E-06 |
| AC020907.4 | 1.402191 | 1.264002 | 1.555488 | 1.71E-10 |
| PSPC1-AS2 | 1.664631 | 1.373433 | 2.017569 | 2.06E-07 |
| AL354836.1 | 1.052289 | 1.031943 | 1.073037 | 3.11E-07 |
| AC093484.4 | 1.435629 | 1.17386 | 1.755772 | 0.00043 |
| AC025766.1 | 1.501316 | 1.186407 | 1.899811 | 0.000717 |
| AC027796.4 | 1.312271 | 1.198007 | 1.437433 | 5.01E-09 |
| AL161935.1 | 2.346346 | 1.500479 | 3.669054 | 0.000185 |
| AC005387.2 | 1.823652 | 1.42598 | 2.332224 | 1.69E-06 |
| AC099850.3 | 1.12215 | 1.079265 | 1.16674 | 6.76E-09 |
| ZNNT1 | 1.178043 | 1.105436 | 1.255418 | 4.46E-07 |
| AC004687.1 | 1.19588 | 1.080761 | 1.323261 | 0.000532 |
| AC127024.5 | 1.302443 | 1.152379 | 1.472048 | 2.33E-05 |
| AL139349.1 | 1.102843 | 1.062483 | 1.144736 | 2.66E-07 |
| AC017083.1 | 3.151426 | 2.093066 | 4.744945 | 3.85E-08 |
| AC004908.1 | 1.242745 | 1.163594 | 1.327279 | 9.64E-11 |
| AC108673.3 | 1.120992 | 1.075468 | 1.168444 | 6.68E-08 |
| LINC01138 | 1.567091 | 1.364032 | 1.800379 | 2.23E-10 |
| ARHGEF2-AS2 | 3.364434 | 2.503009 | 4.522323 | 8.99E-16 |
| AC097641.2 | 2.662357 | 1.923699 | 3.684643 | 3.51E-09 |
| TFAP2E-AS1 | 2.030622 | 1.464675 | 2.81525 | 2.14E-05 |
| AC012615.6 | 1.520211 | 1.262197 | 1.830966 | 1.02E-05 |
| AL731567.1 | 1.364045 | 1.228164 | 1.51496 | 6.68E-09 |
| AC011466.1 | 3.179441 | 1.638433 | 6.169824 | 0.000627 |
| PKD1P6-NPIPP1 | 3.284285 | 2.064592 | 5.224533 | 5.15E-07 |
| AC008870.2 | 2.293266 | 1.79404 | 2.931411 | 3.45E-11 |
| AC103706.1 | 1.542814 | 1.39352 | 1.708103 | 6.81E-17 |
| AL513218.1 | 1.73317 | 1.463792 | 2.05212 | 1.76E-10 |
| AC106782.5 | 1.441298 | 1.193222 | 1.740949 | 0.000149 |
| AL031670.1 | 1.737554 | 1.385447 | 2.179148 | 1.74E-06 |
| AC078906.1 | 1.640437 | 1.242435 | 2.165934 | 0.000481 |
| AC015813.1 | 1.205723 | 1.117903 | 1.300442 | 1.24E-06 |
| AL022238.2 | 1.908082 | 1.415229 | 2.572573 | 2.26E-05 |
| U91328.3 | 1.748118 | 1.426441 | 2.142337 | 7.32E-08 |
| AL158212.2 | 1.879309 | 1.501622 | 2.351991 | 3.56E-08 |
| U62317.1 | 1.061948 | 1.042175 | 1.082095 | 3.66E-10 |
| AL390728.5 | 1.070252 | 1.043313 | 1.097887 | 1.79E-07 |
| MIAT | 1.078754 | 1.035074 | 1.124277 | 0.000325 |
| INE1 | 1.328406 | 1.179354 | 1.496296 | 2.91E-06 |
| AC010326.3 | 1.142786 | 1.093222 | 1.194597 | 3.64E-09 |
| AC026333.4 | 3.264002 | 2.20651 | 4.828307 | 3.19E-09 |
| AC147067.1 | 1.275048 | 1.134088 | 1.433527 | 4.80E-05 |
| AC129510.1 | 1.440774 | 1.264747 | 1.6413 | 3.96E-08 |
| AC026471.4 | 1.050584 | 1.02395 | 1.077911 | 0.000166 |
| FOXD2-AS1 | 1.551883 | 1.37898 | 1.746464 | 3.06E-13 |
| SNHG3 | 1.142801 | 1.103044 | 1.183991 | 1.48E-13 |
| AC015819.2 | 1.066676 | 1.028052 | 1.106751 | 0.000603 |
| VPS9D1-AS1 | 1.536746 | 1.359255 | 1.737414 | 6.81E-12 |
| AL031186.1 | 1.643797 | 1.319127 | 2.048375 | 9.55E-06 |
| AC010761.1 | 1.277313 | 1.140544 | 1.430482 | 2.28E-05 |
| LENG8-AS1 | 1.133232 | 1.0765 | 1.192953 | 1.81E-06 |
| AL161669.3 | 1.094682 | 1.043747 | 1.148103 | 0.000198 |
| AC008105.2 | 1.32378 | 1.195438 | 1.4659 | 7.01E-08 |
| AC114730.3 | 1.754638 | 1.342005 | 2.294146 | 3.95E-05 |
| AC010809.2 | 2.374919 | 1.716232 | 3.286409 | 1.80E-07 |
| AL596223.2 | 1.69601 | 1.308131 | 2.198901 | 6.68E-05 |
| AC015660.3 | 1.870134 | 1.404416 | 2.490287 | 1.83E-05 |
| AC018638.7 | 1.426555 | 1.191355 | 1.708188 | 0.000111 |
| HM13-IT1 | 1.336197 | 1.198924 | 1.489187 | 1.60E-07 |
| AC104564.3 | 1.323634 | 1.151041 | 1.522105 | 8.38E-05 |
| SH3BP5-AS1 | 1.207352 | 1.086953 | 1.341087 | 0.000439 |
| AC005306.1 | 2.255958 | 1.647089 | 3.089904 | 4.00E-07 |
| LINC02804 | 1.624231 | 1.236324 | 2.133849 | 0.000495 |
| AL031714.1 | 1.487742 | 1.223018 | 1.809767 | 7.07E-05 |
| CEP250-AS1 | 2.148594 | 1.448769 | 3.186469 | 0.000143 |
| AC008735.2 | 1.094424 | 1.052983 | 1.137497 | 4.62E-06 |
| MIR155HG | 1.074023 | 1.042713 | 1.106274 | 2.24E-06 |
| AC068620.2 | 2.362925 | 1.865358 | 2.993214 | 1.02E-12 |
| AC027271.1 | 3.000738 | 2.286142 | 3.938701 | 2.41E-15 |
| RRN3P2 | 2.15673 | 1.580166 | 2.943669 | 1.28E-06 |
| PDXDC2P-NPIPB14P | 1.69061 | 1.407121 | 2.031212 | 2.06E-08 |
| AC007497.1 | 1.825533 | 1.324969 | 2.515207 | 0.000232 |
| AL096865.1 | 1.785812 | 1.468215 | 2.17211 | 6.48E-09 |
| AC020658.5 | 2.076514 | 1.585016 | 2.72042 | 1.14E-07 |
| AC012645.4 | 1.981594 | 1.519781 | 2.583738 | 4.38E-07 |
| AP006621.2 | 1.140664 | 1.06924 | 1.216859 | 6.63E-05 |
| AC018648.1 | 2.203201 | 1.764869 | 2.750399 | 2.97E-12 |
| MYG1-AS1 | 1.191226 | 1.119749 | 1.267265 | 2.98E-08 |
| LINC00342 | 1.172093 | 1.114652 | 1.232494 | 5.88E-10 |
| AC131009.3 | 1.229446 | 1.12223 | 1.346906 | 9.12E-06 |
| AC005253.1 | 1.469464 | 1.180355 | 1.829386 | 0.000574 |
| AC232271.1 | 1.390445 | 1.217479 | 1.587986 | 1.15E-06 |
| MUC12-AS1 | 1.162777 | 1.108323 | 1.219906 | 7.15E-10 |
| AC011462.4 | 1.201531 | 1.125159 | 1.283088 | 4.27E-08 |
| AL442125.1 | 4.367638 | 2.433952 | 7.837565 | 7.75E-07 |
| AC010883.1 | 1.235281 | 1.129589 | 1.350862 | 3.65E-06 |
| PVT1 | 1.165813 | 1.10362 | 1.231512 | 4.14E-08 |
| ITGB2-AS1 | 1.129258 | 1.070013 | 1.191783 | 9.82E-06 |
| AC005387.1 | 1.964827 | 1.542898 | 2.502139 | 4.35E-08 |
| AC067945.2 | 2.603646 | 1.682512 | 4.029079 | 1.74E-05 |
| AC069281.2 | 2.028219 | 1.645582 | 2.499828 | 3.37E-11 |
| AC092118.2 | 1.592532 | 1.303984 | 1.944931 | 5.06E-06 |
| AC005261.3 | 1.236422 | 1.168177 | 1.308653 | 2.37E-13 |
| AC108134.3 | 1.255353 | 1.126892 | 1.398459 | 3.65E-05 |
| AC243960.1 | 1.259039 | 1.132641 | 1.399542 | 1.98E-05 |
| AL139123.1 | 3.063931 | 2.255285 | 4.162522 | 7.96E-13 |
| AF117829.1 | 1.386206 | 1.164642 | 1.64992 | 0.000238 |
| MMP25-AS1 | 1.183549 | 1.087557 | 1.288013 | 9.43E-05 |
| AL359504.1 | 1.714184 | 1.393614 | 2.108494 | 3.36E-07 |
| AP002807.1 | 1.51103 | 1.344588 | 1.698075 | 4.13E-12 |
| AC145098.1 | 1.349685 | 1.137832 | 1.600983 | 0.000577 |
| PCED1B-AS1 | 1.116828 | 1.061455 | 1.175089 | 2.06E-05 |
| LINC02747 | 0.964176 | 0.949752 | 0.978819 | 2.10E-06 |
| AL159169.2 | 1.914673 | 1.457492 | 2.51526 | 3.07E-06 |
| AC005785.1 | 1.67996 | 1.437373 | 1.963489 | 7.04E-11 |
| AC027601.1 | 4.075361 | 2.565434 | 6.473978 | 2.69E-09 |
| AC124319.1 | 1.43062 | 1.22773 | 1.667038 | 4.45E-06 |
| AC005837.4 | 1.233239 | 1.092655 | 1.391912 | 0.000687 |
| AC020558.2 | 1.855123 | 1.476609 | 2.330665 | 1.11E-07 |
| LINC02604 | 1.164433 | 1.108415 | 1.223282 | 1.43E-09 |
| PARD3-AS1 | 1.170022 | 1.077337 | 1.27068 | 0.000192 |
| Z84485.1 | 1.385127 | 1.213994 | 1.580383 | 1.29E-06 |
| AL451050.2 | 4.406314 | 2.860984 | 6.786338 | 1.69E-11 |
| AC092171.4 | 1.419743 | 1.239614 | 1.626046 | 4.13E-07 |
| AL360181.2 | 1.172254 | 1.096125 | 1.253671 | 3.50E-06 |
| AL353801.3 | 1.637397 | 1.237502 | 2.166516 | 0.000557 |
| LASTR | 1.182921 | 1.119134 | 1.250343 | 2.85E-09 |
| AC010245.2 | 1.955503 | 1.532624 | 2.495062 | 6.87E-08 |
| AC004034.1 | 3.006498 | 2.116964 | 4.269808 | 7.73E-10 |
| MATN1-AS1 | 1.64461 | 1.269618 | 2.130358 | 0.000165 |
| IGBP1-AS1 | 2.968794 | 2.026502 | 4.349238 | 2.33E-08 |
| AC004908.3 | 1.59961 | 1.382504 | 1.85081 | 2.75E-10 |
| AC073575.2 | 2.7638 | 1.842082 | 4.146717 | 9.05E-07 |
| AL359921.1 | 1.857542 | 1.347581 | 2.560487 | 0.000156 |
| AC110285.2 | 1.111457 | 1.051938 | 1.174345 | 0.000168 |
| AC010973.2 | 1.486306 | 1.309765 | 1.686642 | 8.11E-10 |
| ZNF32-AS2 | 1.515104 | 1.245363 | 1.843271 | 3.27E-05 |
| UBE2Q1-AS1 | 2.401772 | 1.681555 | 3.43046 | 1.45E-06 |
| AL928654.2 | 1.139504 | 1.06518 | 1.219013 | 0.000148 |
| RAP2C-AS1 | 0.240019 | 0.113828 | 0.506107 | 0.000177 |
| AL021707.1 | 2.352105 | 1.582746 | 3.495442 | 2.32E-05 |
| AC090589.3 | 1.357307 | 1.182478 | 1.557985 | 1.41E-05 |
| ZNF436-AS1 | 1.373922 | 1.213327 | 1.555773 | 5.47E-07 |
| AC040162.3 | 2.642862 | 1.879286 | 3.716689 | 2.32E-08 |
| SCAT2 | 1.364636 | 1.244787 | 1.496024 | 3.39E-11 |
| HOXB-AS1 | 1.09313 | 1.039058 | 1.150016 | 0.000581 |
| AC016957.2 | 1.806039 | 1.430508 | 2.280153 | 6.69E-07 |
| AC087289.5 | 2.379927 | 1.609284 | 3.519609 | 1.40E-05 |
| AP000892.3 | 2.063622 | 1.38503 | 3.074689 | 0.000369 |
| LINC00528 | 2.187282 | 1.518553 | 3.150501 | 2.62E-05 |
| AL354760.1 | 2.75313 | 2.079188 | 3.645522 | 1.55E-12 |
| SNHG17 | 1.154702 | 1.105996 | 1.205554 | 6.08E-11 |
| AC003070.1 | 1.260626 | 1.120013 | 1.418892 | 0.000124 |
| AL023653.1 | 1.896088 | 1.507282 | 2.385186 | 4.65E-08 |
| AL122125.1 | 1.552565 | 1.219621 | 1.976399 | 0.000354 |
| AC008760.1 | 1.400946 | 1.18192 | 1.660559 | 0.000102 |
| MELTF-AS1 | 1.171638 | 1.128591 | 1.216328 | 1.10E-16 |
| LINC00893 | 1.4341 | 1.227569 | 1.675379 | 5.51E-06 |
| AC087289.2 | 4.613809 | 2.75123 | 7.737352 | 6.77E-09 |
| AL662797.1 | 3.921585 | 2.643162 | 5.818344 | 1.13E-11 |
| H1-10-AS1 | 1.963436 | 1.455371 | 2.648865 | 1.00E-05 |
| AC008764.8 | 2.138006 | 1.637195 | 2.792011 | 2.40E-08 |
| ZEB2-AS1 | 2.496552 | 1.602447 | 3.889533 | 5.25E-05 |
| MRPL20-DT | 1.704978 | 1.454907 | 1.998032 | 4.31E-11 |
| PTOV1-AS2 | 1.138968 | 1.084039 | 1.196679 | 2.47E-07 |
| AC007038.1 | 1.149354 | 1.06965 | 1.234998 | 0.000147 |
| AC004264.1 | 1.1193 | 1.068955 | 1.172016 | 1.59E-06 |
| AC021851.1 | 3.298453 | 1.818856 | 5.981667 | 8.51E-05 |
| AC008610.1 | 1.131615 | 1.077387 | 1.188572 | 8.02E-07 |
| AC104758.1 | 2.192079 | 1.650089 | 2.912091 | 6.09E-08 |
| FSIP2-AS1 | 2.699623 | 1.852213 | 3.934735 | 2.38E-07 |
| AC017104.1 | 1.922342 | 1.4529 | 2.543463 | 4.76E-06 |
