# Supplementary figures and images for: A Novel Pyroptosis-Related lncRNAs Signature for Predicting the Prognosis of Kidney Renal Clear Cell Carcinoma and Its Associations with Immunity
Source: J Oncol. 2021 Oct 18;2021:9997185. doi: 10.1155/2021/9997185 (PMC8577956; doi:10.1155/2021/9997185)

Volcano Plot

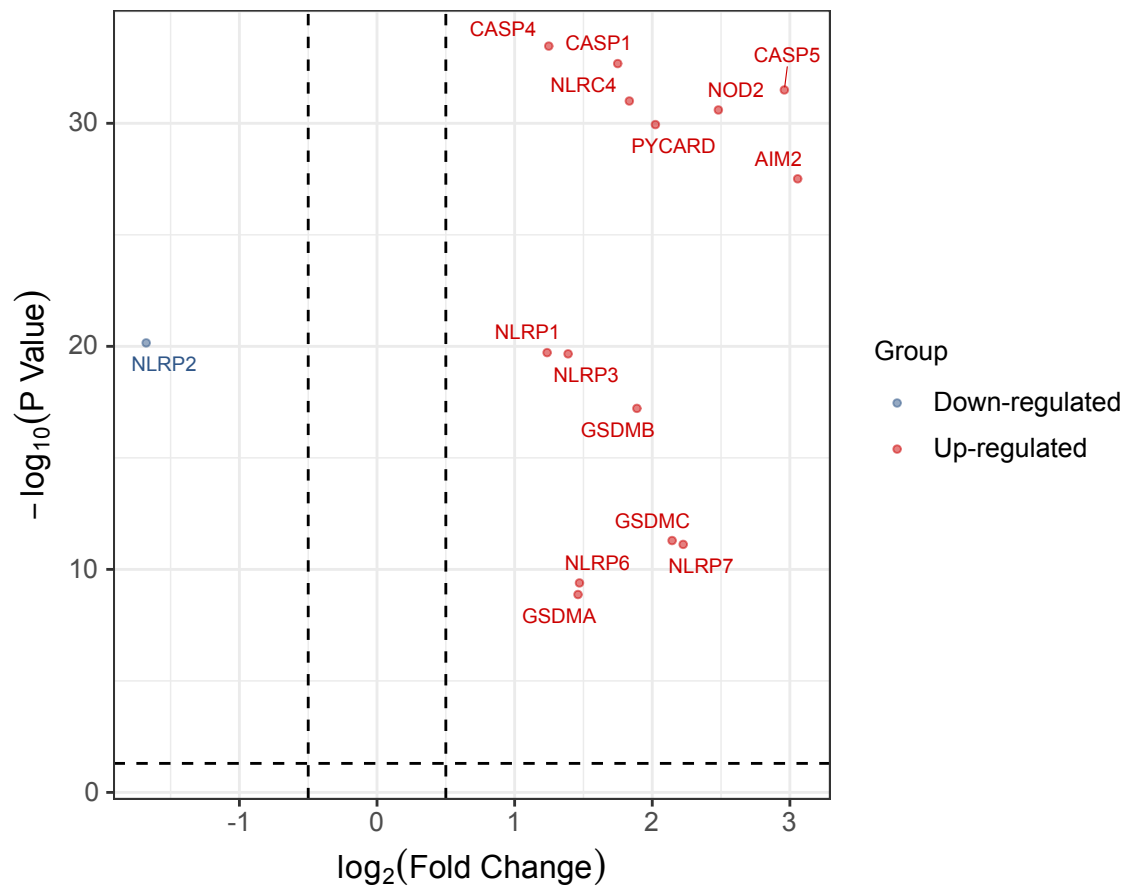

Supplement: Supplementary Materials — Supplementary File Table S1. Patients' clinical features from the TCGA dataset. Supplementary File Table S2. 33 pyroptosis-related genes. Supplementary File Table S3. The sequences of primers and siRNAs used in this study. Supplementary File Table S4. 14 pyroptosis-related DEGs from TCGA-KIRC. Supplementary File Table S5. 1042 pyroptosis-related lncRNAs. Supplementary File Table S6. 299 significant pyroptosis-related lncRNAs after univariate Cox analysis. Supplementary File Figure S1. 14 pyroptosis-related DEGs from TCGA-KIRC. [file 9997185.f1.zip › 9997185.f1/FigureS1.pdf]
